# Supplementary material for: Assessment of quality of antenatal care services in public sector facilities in India
Source: BMJ Open. 2022 Dec 1;12(12):e065200. doi: 10.1136/bmjopen-2022-065200 (PMC9716787; doi:10.1136/bmjopen-2022-065200)

Supplementary Table 1: Select characteristics of the pregnant women who participated in the exit interviews.

Background characteristics	All women				Women in Gaya district				Women in Supaul district			
	Total N=814	In 1st trimester N =103 (%)	In 2nd trimester N=405 (%)	In 3rd trimester N=306 (%)	Total N=549	In 1st trimester of pregnancy N=69 (%)	In 2nd trimester of pregnancy N=273 (%)	In 3rd trimester of pregnancy N=207 (%)	Total N=265	In 1st trimester N=34 (%)	In 2nd trimester N=132 (%)	In 3rd trimester N=99 (%)
**Maternal age**												
15-19 years	**118**	13 (12.6)	68 (16.8)	37 (12.1)	**74**	9 (13.0)	43 (15.7)	22 (10.7)	**44**	4 (11.8)	25 (18.9)	15 (15.1)
20-24 years	**488**	69 (67.0)	242 (59.7)	176 (57.7)	**332**	46 (66.7)	159 (58.2)	126 (61.2)	**156**	23 (67.6)	83 (62.9)	50 (50.5)
25-29 years	**174**	18 (17.5)	83 (20.5)	73 (23.9)	**119**	13 (18.8)	62 (22.7)	44 (21.4)	**55**	5 (14.7)	21 (15.9)	29 (29.3)
30-40 years	**34**	3 (2.9)	12 (3.0)	19 (6. 2)	**24**	1 (1.4)	9 (3.3)	14 (6.8)	**10**	2 (5.9)	3 (2.3)	5 (5.1)
**Maternal caste***												
Scheduled caste/tribe	**259**	27 (26.7)	129 (32.3)	103 (34.3)	**201**	25 (36.2)	98 (35.9)	78 (37.7)	**58**	2 (6.3)	31 (24.4)	25 (26.9)
Other backward class	**457**	64 (63.4)	232 (58.0)	161 (53.7)	**311**	39 (56.5)	158(57.9)	114(55.1)	**146**	25 (78.1)	74 (58.3)	47 (50.5)
General	**85**	10 (9.9)	39 (9.7)	36 (12.0)	**37**	5 (7.3)	17 (6.2)	15 (7.3)	**48**	5 (15.6)	22 (17.3)	21 (22.6)
**Number of ANC visit on the day of interview**												
1st visit	**259**	61 (59.2)	134(33.1)	64 (20.9)	**189**	44 (63.7)	101 (37.0)	44 (21.3)	**70**	17 (50.0)	33 (25.0)	20 (20.2)
2nd or 3rd visit	**273**	33 (32.0)	145(35.8)	95 (31.0)	**177**	19 (27.5)	90 (32.9)	68 (32.8)	**96**	14 (41.2)	55 (41.6)	27 (27.3)
4th visit or more	**282**	9 (8.7)	126 31.1)	147 (48.0)	**183**	6 (8.7)	82 (30.0)	95 (45.9)	**99**	3 (8.8)	44 (33.3)	52 (52.5)

**Data not available for 13 pregnant women*

Supplementary Table 2. Coverage of quality ANC services as reported in the exit interviews on the PMSMA day with maternal age and caste of women. CI denotes confidence interval.

Background	All women					Women in Gaya district					Women in Supaul district				
	**Total N=814**	**In 1st trimester with abdomen check-up N (%; (95% CI)**	**In 1st trimester without abdomen check-up N (%; (95% CI)**	**In 2nd trimester N (%; (95% CI)**	**In 3rd trimester N (%; (95% CI)**	**Total N=549**	**In 1st trimester with abdomen check-up N (%; (95% CI)**	**In 1st trimester without abdomen check-up N (%; (95% CI))**	**In 2nd trimester N (%; (95% CI)**	**In 3rd trimester N (%; (95% CI)**	**Total N=265**	**In 1st trimester with abdomen check-up N (%; (95% CI)**	**In 1st trimester without abdomen check-up N (%; (95% CI)**	**In 2nd trimester N (%; (95% CI)**	**In 3rd trimester N (%; (95% CI)**
**Maternal age**															
15-19 years	**118**	15.4 (3.6-47.1)	38.5 (16.1-67.0)	32.3 (22.2-44.4)	40.5 (25.9-57.1)	**74**	0	33.3 (10.1-69.1)	34.9 (22.1-50.3)	50.0 (29.7-70.3)	**44**	50.0 (8.7-91.3)	50.0 (8.7-91.3)	28.0 (13.7-48.9)	26.7 (9.9-54.7)
20-24 years	**488**	4.3 (1.4-12.9)	24.6 (15.7-36.3)	33.5 (27.8-39.7)	29.5 (23.2-36.7)	**332**	2.2 (0.3-14.6)	28.3 (16.9-43.3)	38.4 (31.1-46.2)	28.6 (21.3-37.1)	**156**	8.7 (2.0-30.7)	17.4 (6.3-39.8)	24.1 (16.0-34.6)	32.0 (20.4-46.3)
25-29 years	**174**	11.1 (2.6-36.6)	27.8 (11.6-53.0)	40.9 (30.8-51.9)	31.5 (21.8-43.1)	**119**	15.4 (3.6-47.3)	23.1 (7.1-54.1)	40.3 (28.8-53.0)	25.0 (14.3-40.0)	**55**	0	40.0 (7.7-84.2)	42.8 (23.5-64.7)	41.4 (24.8-60.2)
30-40 years	**34**	0	66.7 (9.3-97.5)	33.3 (12.4-63.7)	47.4 (26.2-69.5)	**24**	0	0	33.3 (10.2-68.6)	28.6 (10.7-57.3)	**10**	0	100.0	33.3 (2.5-90.7)	100.0
**Maternal caste***															
Scheduled caste/tribe	**259**	3.7 (0.5-23.2)	22.2 (10.1-42.1)	34.1 (26.4-42.8)	32.0 (23.7-41.7)	**201**	4.0 (0.5-25.0)	24.0 (10.8-45.0)	35.7 (26.8-45.7)	34.6 (24.8-45.9)	**58**	0	0	29.0 (15.6-47.6)	24.0 (10.9-44.9)

Other backward class	**457**	9.4 (4.2-19.6)	31.3 (21.0-43.8)	33.6 (27.8-40.0)	31.7 (24.9-39.3)	**311**	5.1 (1.2-19.0)	33.3 (20.1-49.8)	38.6 (31.3-46.5)	30.7 (22.9-39.8)	**146**	16.0 (5.8-37.2)	28.0 (13.3-49.6)	23.0 (14.7-34.1)	34.0 (21.8-48.9)
General	**85**	0	20.0 (4.6-56.6)	38.5 (24.5-54.6)	33.3 (19.8-50.3)	**37**	0	0	47.1 (24.9-70.4)	6.7 (0.9-37.1)	**48**	0	40.0 (7.7-84.2)	31.8 (15.6-54.1)	52.4 (31.1-72.8)

**Data not available for 13 pregnant women*

Supplementary Table 3: Availability of the staff on the PMSMA day to provide antenatal care services. NA denotes not applicable.

District, Facility	Number of staff							Total pregnant women registered for ANC services	Average number of pregnant women				Coverage of quality ANC services as reported in the exit interviews
	Doctor	Staff nurse	Lab technician	ANM	Counsellor	Pharmacist	Total		Per staff	Per doctor	Per ANM	Per lab technician	
**Gaya, CHC 1**													
Round 1	1	0	1	3	0	1	6	70	12	70	23.3	70	74.5
Round 2	2	0	0	4	0	1	7	69	10	34.5	17.3	NA	0
Round 3	2	0	1	5	0	1	9	74	8	37	14.8	74	28.3
**Gaya, CHC 2**													
Round 1	1	0	2	4	0	1	8	65	8	65	16.3	32.5	50.0
Round 2	1	0	2	4	0	1	8	41	5	41	10.3	20.5	90.3
Round 3	1	0	2	2	0	1	6	79	13	79	39.5	39.5	71.4
**Gaya, SDH**													
Round 1	2	2	2	2	1	1	10	89	9	44.5	44.5	44.5	4.4

District, Facility	Number of staff							Total pregnant women registered for ANC services	Average number of pregnant women				Coverage of quality ANC services as reported in the exit interviews
	Doctor	Staff nurse	Lab technician	ANM	Counsellor	Pharmacist	Total		Per staff	Per doctor	Per ANM	Per lab technician	
Round 2	2	0	1	2	1	1	7	71	10	35.5	35.5	71	0
Round 3	4	0	1	2	0	1	8	102	12	25.5	51	102	4.3
**Supaul, CHC 1**													
Round 1	3	0	1	12	0	0	16	122	8	40.7	10.2	122	88.9
**Supaul, CHC 2**													
Round 1	1	0	1	5	0	0	7	118	16	118	23.6	118	1.5
**Supaul, SDH**													
Round 1	2	1	2	5	1	1	12	124	10	62	24.8	62	5.1

Supplementary Figure 1: Coverage of ANC service components as reported by the pregnant women in exit survey on the PMSMA day. Bars denote 95% confidence interval, CHC Community Health Centre and SDH Sub-district hospital.

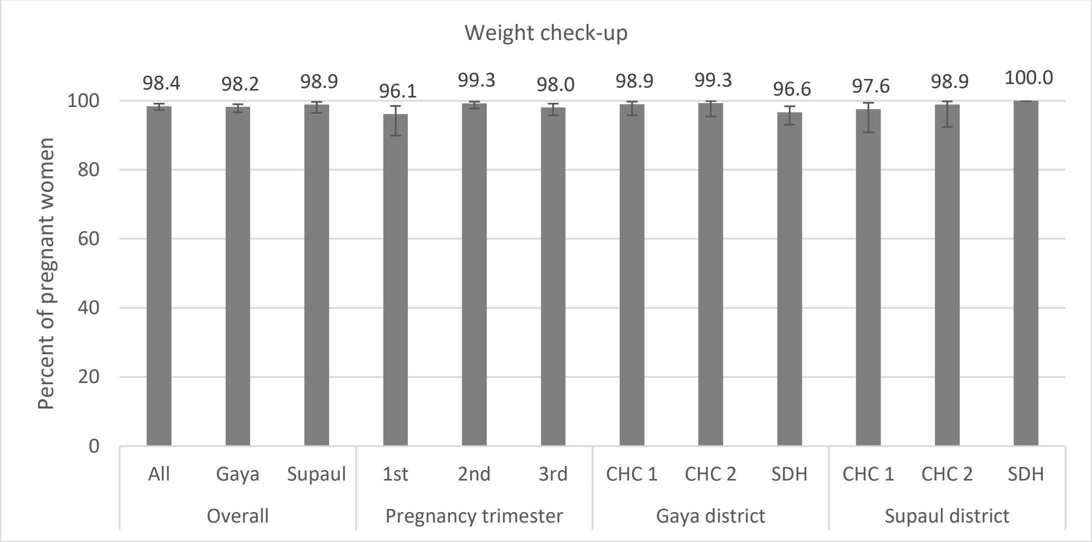

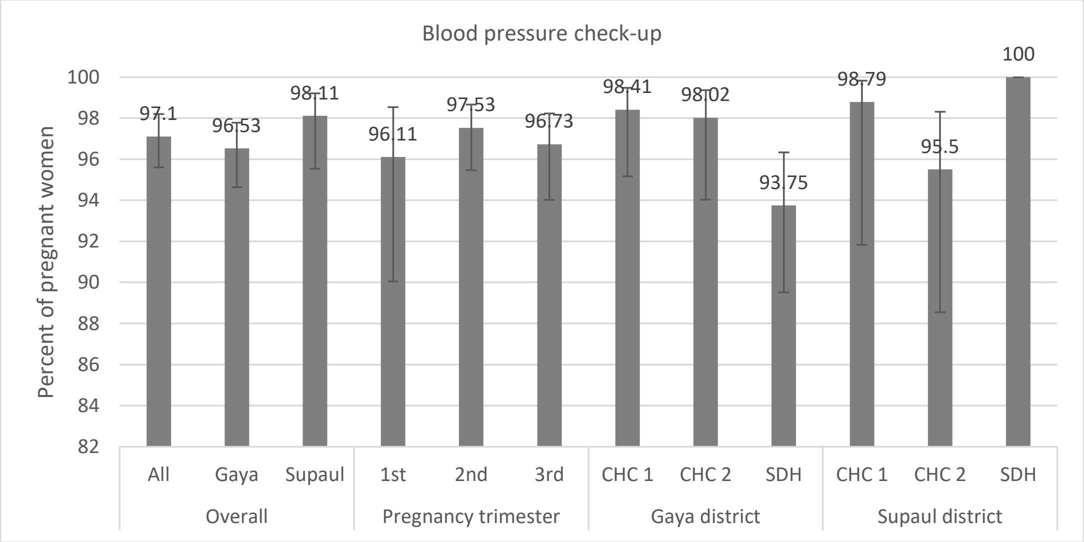

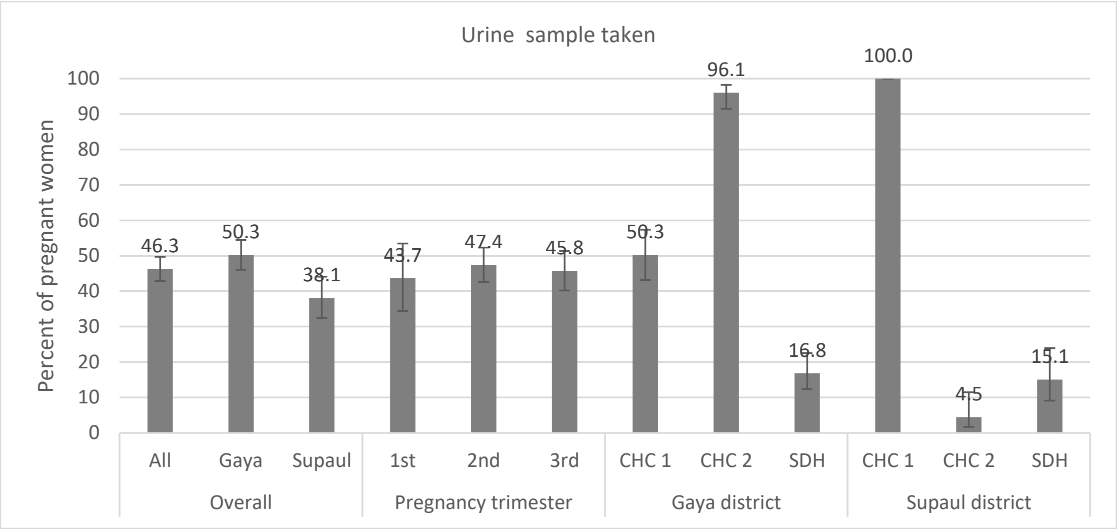
Urine sample taken
Percent of pregnant women
0
10
20
30
40
50
60
70
80
90
100
46.3
50.3
38.1
43.7
47.4
45.8
50.3
96.1
16.8
100.0
4.5
15.1
All
Gaya
Supaul
1st
2nd
3rd
CHC 1
CHC 2
SDH
CHC 1
CHC 2
SDH
Overall
Pregnancy trimester
Gaya district
Supaul district

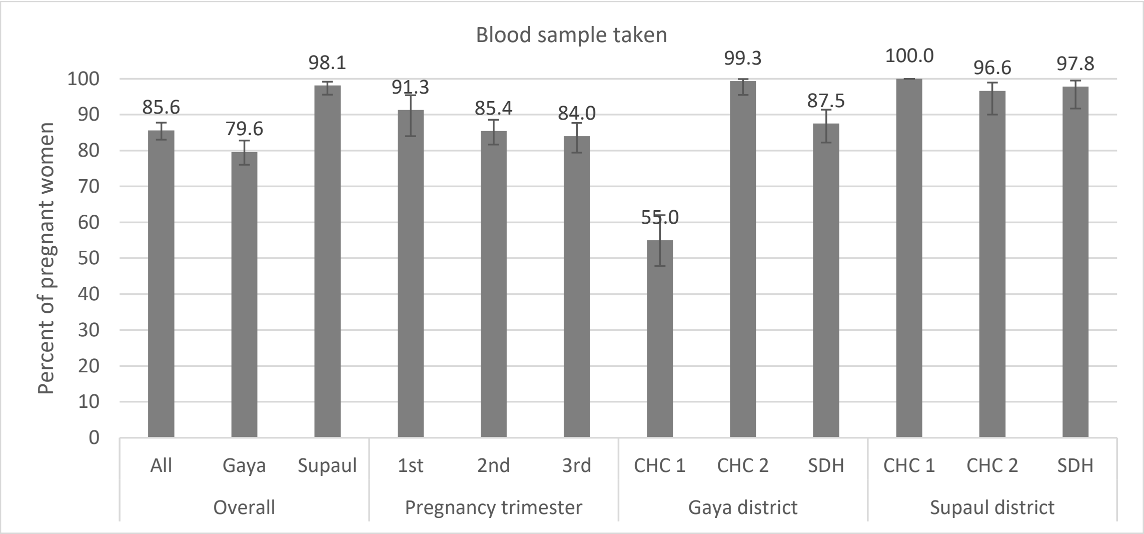
Blood sample taken
Percent of pregnant women
0
10
20
30
40
50
60
70
80
90
100
85.6
79.6
98.1
91.3
85.4
84.0
55.0
99.3
87.5
100.0
96.6
97.8
All
Gaya
Supaul
1st
2nd
3rd
CHC 1
CHC 2
SDH
CHC 1
CHC 2
SDH
Overall
Pregnancy trimester
Gaya district
Supaul district

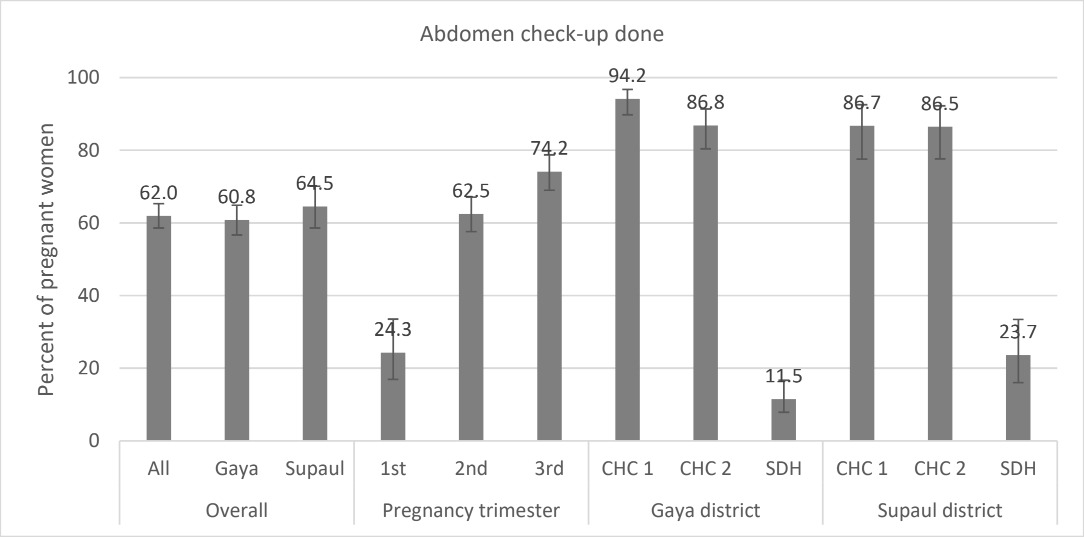
Abdomen check-up done
Percent of pregnant women
100
80
60
40
20
0
62.0
60.8
64.5
24.3
62.5
74.2
94.2
86.8
11.5
86.7
86.5
23.7
All
Gaya
Supaul
1st
2nd
3rd
CHC 1
CHC 2
SDH
CHC 1
CHC 2
SDH
Overall
Pregnancy trimester
Gaya district
Supaul district

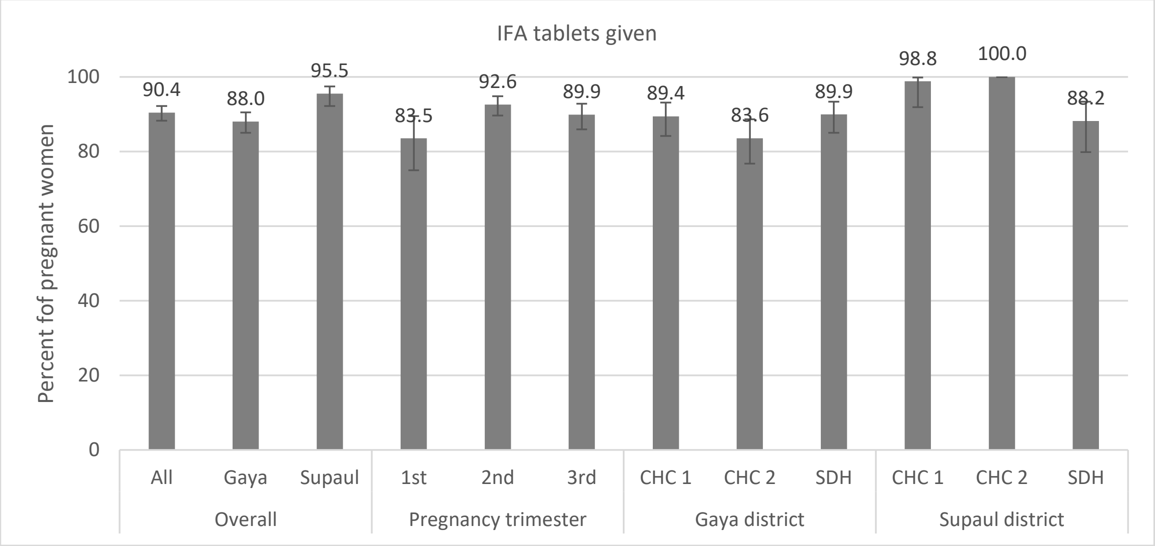
IFA tablets given
Percent fof pregnant women
100
80
60
40
20
0
90.4
88.0
95.5
83.5
92.6
89.9
89.4
83.6
89.9
98.8
100.0
88.2
All
Gaya
Supaul
1st
2nd
3rd
CHC 1
CHC 2
SDH
CHC 1
CHC 2
SDH
Overall
Pregnancy trimester
Gaya district
Supaul district

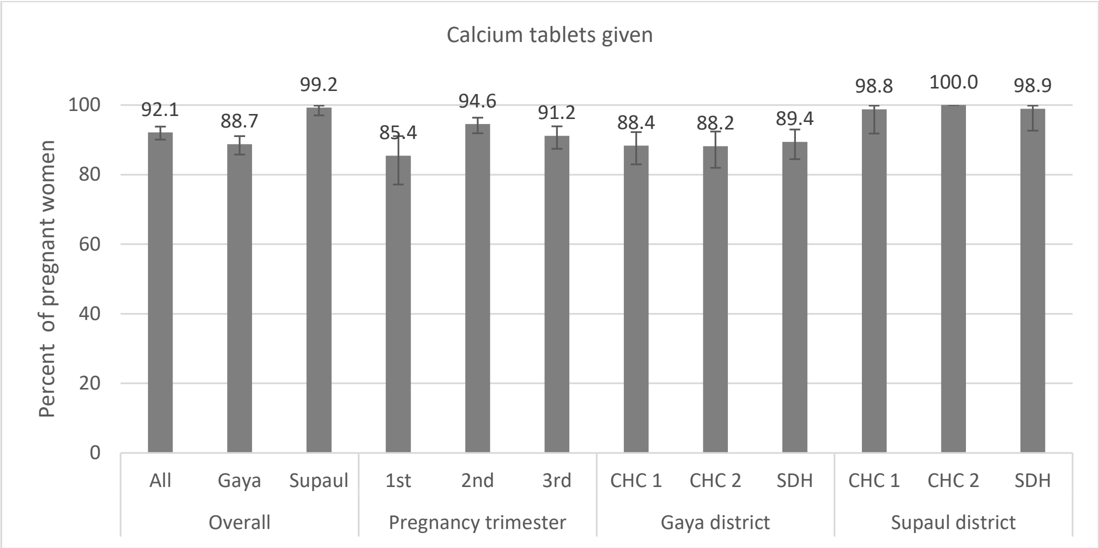
Calcium tablets given
Percent of pregnant women
0
20
40
60
80
100
92.1
88.7
99.2
85.4
94.6
91.2
88.4
88.2
89.4
98.8
100.0
98.9
All
Gaya
Supaul
1st
2nd
3rd
CHC 1
CHC 2
SDH
CHC 1
CHC 2
SDH
Overall
Pregnancy trimester
Gaya district
Supaul district

Supplementary Figure 2. Coverage of each component of antenatal care (ANC) and quality of ANC services among the 306 pregnant women in their 3rd trimester by the number of their ANC visit.

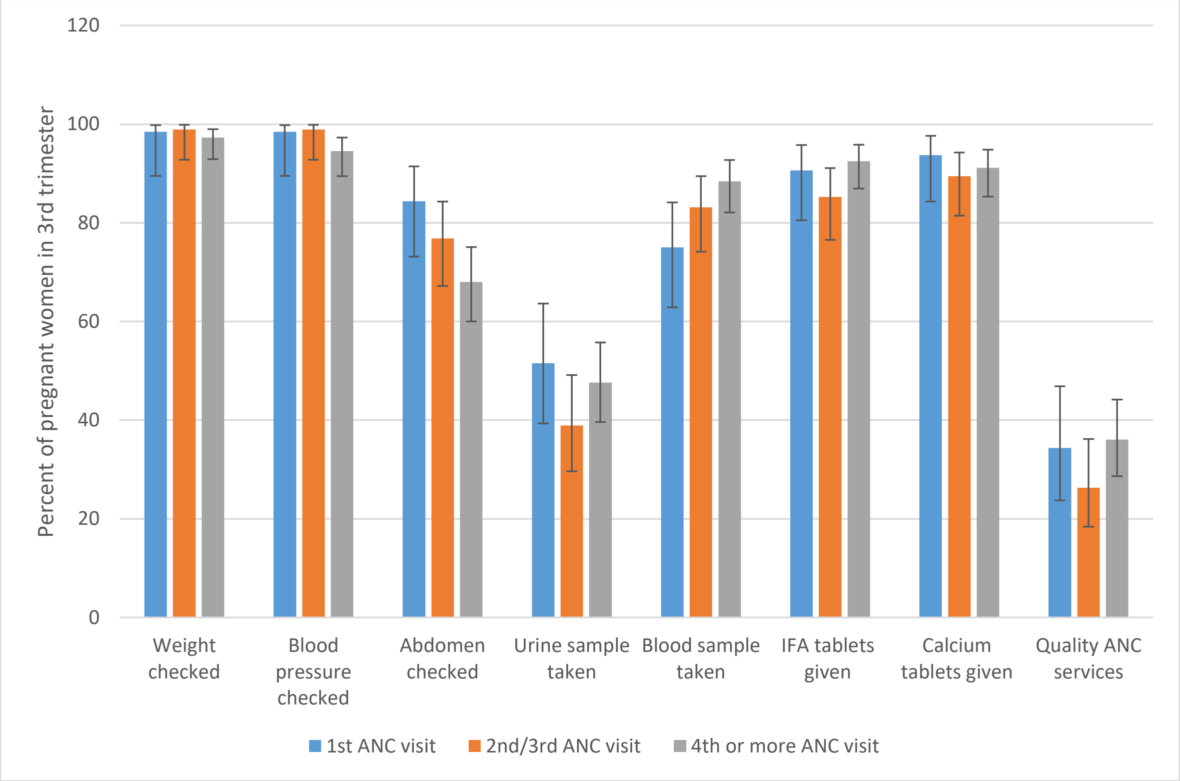

Supplmentary Figure 3: Make-shift toilet facility in one of the public sector health facilities for pregnant women to provide urine sample on PMSMA day.

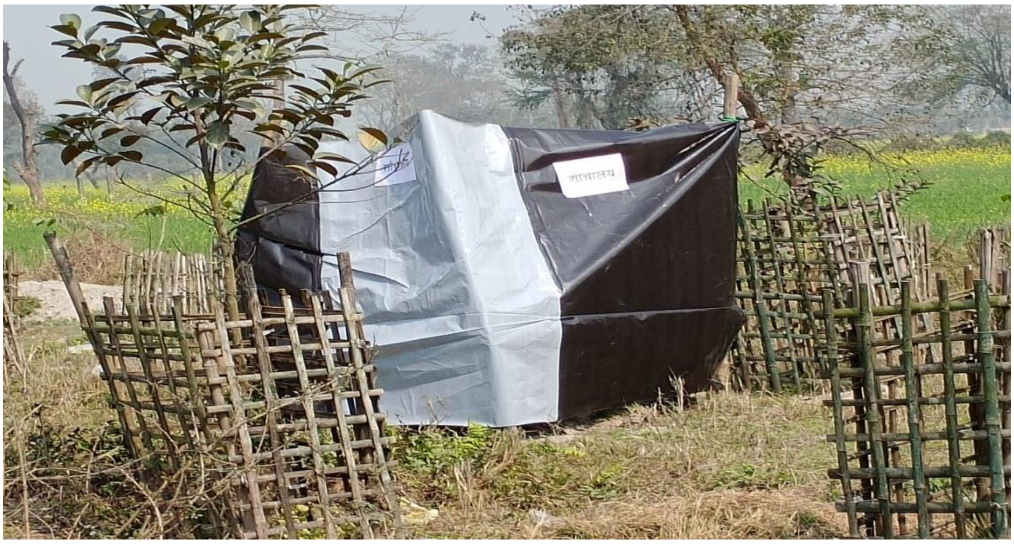

Supplement: Supplementary data [file bmjopen-2022-065200supp001.pdf]
