# Supplementary material for: Assessment of quality of antenatal care services in public sector facilities in India
Source: BMJ Open. 2022 Dec 1;12(12):e065200. doi: 10.1136/bmjopen-2022-065200 (PMC9716787; doi:10.1136/bmjopen-2022-065200)
Supplement: Author's manuscript [file bmjopen-2022-065200.draft_revisions.pdf]

BMJ Open

BMJ Open is committed to open peer review. As part of this commitment we make the peer review history of every article we publish publicly available.

When an article is published we post the peer reviewers' comments and the authors' responses online. We also post the versions of the paper that were used during peer review. These are the versions that the peer review comments apply to.

The versions of the paper that follow are the versions that were submitted during the peer review process. They are not the versions of record or the final published versions. They should not be cited or distributed as the published version of this manuscript.

BMJ Open is an open access journal and the full, final, typeset and author-corrected version of record of the manuscript is available on our site with no access controls, subscription charges or pay-per-view fees (http://bmjopen.bmj.com).

If you have any questions on BMJ Open's open peer review process please email info.bmjopen@bmj.com

Prospective assessment of quality of antenatal care services in public-sector facilities in India: recommendations for action

Journal:	*BMJ Open*
Manuscript ID	bmjopen-2022-065200
Article Type:	Original research
Date Submitted by the Author:	29-May-2022

Complete List of Authors:	Dandona, Rakhi; Public Health Foundation of India; University of Washington<br>Majumder, Moutushi; Public Health Foundation of India<br>Akbar, Md.; Public Health Foundation of India<br>Bhattacharya, Debarshi; Bill & Melinda Gates Foundation India<br>Nanda, Priya; Bill and Melinda Gates Foundation India<br>KUMAR, ANIL; Public Health Foundation of India<br>Dandona, Lalit; Public Health Foundation of India

Keywords:	Maternal medicine < OBSTETRICS, Community child health < PAEDIATRICS, PUBLIC HEALTH

SCHOLARONE™ Manuscripts

I, the Submitting Author has the right to grant and does grant on behalf of all authors of the Work (as defined in the below author licence), an exclusive licence and/or a non-exclusive licence for contributions from authors who are: i) UK Crown employees; ii) where BMJ has agreed a CC-BY licence shall apply, and/or iii) in accordance with the terms applicable for US Federal Government officers or employees acting as part of their official duties; on a worldwide, perpetual, irrevocable, royalty-free basis to BMJ Publishing Group Ltd ("BMJ") its licensees and where the relevant Journal is co-owned by BMJ to the co-owners of the Journal, to publish the Work in this journal and any other BMJ products and to exploit all rights, as set out in our licence.

The Submitting Author accepts and understands that any supply made under these terms is made by BMJ to the Submitting Author unless you are acting as an employee on behalf of your employer or a postgraduate student of an affiliated institution which is paying any applicable article publishing charge ("APC") for Open Access articles. Where the Submitting Author wishes to make the Work available on an Open Access basis (and intends to pay the relevant APC), the terms of reuse of such Open Access shall be governed by a Creative Commons licence – details of these licences and which Creative Commons licence will apply to this Work are set out in our licence referred to above.

Other than as permitted in any relevant BMJ Author's Self Archiving Policies, I confirm this Work has not been accepted for publication elsewhere, is not being considered for publication elsewhere and does not duplicate material already published. I confirm all authors consent to publication of this Work and authorise the granting of this licence.

For peer review only

Prospective assessment of quality of antenatal care services in public-sector facilities in India: recommendations for action

Rakhi Dandona,[1,2] Moutushi Majumder,[1] Md. Akbar,[1] Debarshi Bhattacharya,[3] Priya Nanda,[3] G. Anil Kumar,[1] Lalit Dandona[1,2]

[1]Public Health Foundation of India, Gurugram, National Capital Region, India

[2]Institute for Health Metrics and Evaluation, University of Washington, Seattle, USA

[3]Bill & Melinda Gates Foundation, India Country Office, New Delhi, India

Corresponding author: Prof. Rakhi Dandona, Public Health Foundation of India, Plot 47, Sector 44, Gurugram, Haryana – 122 002, India; rakhi.dandona@phfi.org

Word count: 3,934

For peer review only - http://bmjopen.bmj.com/site/about/guidelines.xhtml

ABSTRACT

Objectives: Inadequate quality of antenatal care (ANC) services is mainly reported from population surveys in developing settings. We undertook a prospective assessment of quality of ANC services in public sector facilities in the Indian state of Bihar state delivered under the national ANC program (PMSMA).

Setting: Three community health centres and one sub-district hospital each in two randomly selected districts of Bihar.

Participants: Pregnant women who sought ANC services under PMSMA irrespective of the pregnancy trimester.

Primary and secondary measures: Quality of ANC services was defined as women receiving all of these services in that visit - weight, blood pressure and abdomen check, urine and blood sample taken, and were given iron and folic acid and calcium tablets. The process of ANC service provision was documented.

Results: Eight hundred fourteen (94.5% participation) women participated. Coverage of quality of ANC services was 30.4% (95% CI 27.3-33.7), similar in both districts but significantly lower for women in the 1st trimester of pregnancy (6.8%, 95% CI 3.3-13.6). This coverage ranged 3% to 83.1% across the facilities. Individually, the coverage of weight and blood pressure check-up, receipt of IFA and calcium tablets, and blood sample collection was >85%. The coverage of urine sample collection was 46.3% (95% CI 42.9-49.7) and of abdomen check-up was 62% (95% CI 58.6-65.3). Poor information sharing post check-up was done with the pregnant women. Varied implementation of ANC service provision was seen in the facilities as compared with the PMSMA guidelines, in particular with laboratory diagnostics and doctor consultation. Task shifting from doctors to ANMs was observed in all facilities.

Conclusions: Grossly inadequate quality ANC services under the PMSMA has implications on achieving the desired maternal and neonatal health outcomes.

For peer review only - http://bmjopen.bmj.com/site/about/guidelines.xhtml

Keywords: Antenatal care, coverage, pregnancy, quality, Bihar, India

What is already known on this topic?

The inadequacy of and inequity in quality of antenatal care (ANC) services in developing countries is mainly documented from the population surveys. The ANC quality in these reports is based on the receipt of a combination of essential services at any point during the pregnancy among women who had at least one visit with a skilled ANC provider, which does not offer nuanced understanding of what happens in the health facility for a given ANC visit, thereby, limiting the actions needed to address the coverage-quality gap in ANC services.

What this study adds?

This study reports on quality of ANC services from prospective assessment undertaken at public sector health facilities. The exit survey with pregnant women immediately after they received the ANC services addresses the issue of recall bias in population surveys. In addition to documenting the significantly inadequate quality of ANC services, the study documents the poor information sharing with the pregnant women based on the check-up, and deviations from the prescribed process of ANC service provision.

How this study might affect research, practice or policy?

The documentation of inadequate quality of ANC services prospectively in a facility setting has major implications for practice and policy. The process assessment undertaken has documented task shifting from doctors to ANMs, and inadequate coverage and quality of laboratory diagnostics, both of which have implications for program to achieve its desired goal of providing quality ANC services. This study highlights the need to undertake implementation research at the health facilities to give practical recommendations to improve the gap between policy and practice.

For peer review only - http://bmjopen.bmj.com/site/about/guidelines.xhtml

BACKGOUND

The inadequacy of and inequity in quality of antenatal care (ANC) services is increasingly receiving attention.[1-7] The key elements of ANC services captured under the facility surveys also indicate inadequate quality of care provision.[8] Concerns about the quality of ANC services have also been reported from India, primarily based on the retrospective data collected under the National Family Health Survey.[1 9-11] The ANC quality in these reports was based on the receipt of a combination of essential services at any point during the pregnancy among women who had at least one visit with a skilled ANC provider, which does not offer nuanced understanding of what happens in the health facility for a given ANC visit, thereby, limiting the actions needed to address the coverage-quality gap in ANC services.

The Government of India increased its focus on improving coverage and quality of ANC services under the *Pradhan Mantri Surakshit Matritva Abhiyan* (PMSMA; Prime Minister's Safe Motherhood Program).[12] PMSMA is based on the premise that if every pregnant woman in India is examined by a physician and appropriately investigated at least once during the PMSMA and then appropriately followed up, the process can result in reduction in the number of maternal and neonatal deaths in the country. With over 18,000 facilities providing PMSMA services, nearly 28.1 million pregnant women have been examined in India under this program since its inception in 2016.[12] However, no comprehensive assessment of ANC services under the PMSMA is available in the public domain. In this context , we undertook a prospective health facility based assessment of ANC services offered on the PMSMA day in the Indian state of Bihar, one of the most populous Indian states.[13] The coverage of at least one ANC visit in the state is at 82% and that for 4 ANC visits at 25.2%,[14] with the neonatal mortality rate in 2017 at 23.4 per 1,000 livebirths and maternal mortality ratio of 230.[15 16] The primary aim of this assessment was to identify areas that needed attention for the PMSMA to achieve its stated goal.

For peer review only - http://bmjopen.bmj.com/site/about/guidelines.xhtml

METHODS

Study design

A cross-sectional facility based prospective study in two districts of Bihar state was undertaken which was approved by the Institutional review board of Public Health Foundation of India. All participants provided written informed consent, and for those who could not read or write, the participant information sheet and consent form were explained by the trained interviewer and a thumb impression obtained. A multi-stage sampling process was followed to select the public sector health facilities for this study. First, two districts were sampled based on the latest available 3+ visits ANC coverage for Bihar for 2016 at the time of planning of this study.[17] All the 38 districts of the state were grouped into two strata as same/above and below the state median coverage for 3+ visits ANC visits (29.5%). We then randomly selected one district each from these two groups for this study – Gaya and Supaul. In each of the two sampled districts, three block-level community health centres (CHC) and one sub-district hospital (SDH) were purposively sampled from the public sector health facilities where detailed assessments were already being undertaken. All pregnant women who reported for ANC services, irrespective of the trimester of pregnancy, on the 9th day of the study month under the PMSMA scheme were considered with the only exclusion criterion being non-availability of a phone number for contact later for follow-up assessment. We aimed to recruit 200 pregnant women in each of the sampled facilities giving a total sample of 1,200 women across the six facilities.

Data collection

Data collection began in September 2019 in Gaya district but was then withheld in October and November 2019 due to floods and socio-political issues in the state, and was next two rounds were completed in December 2019 and January 2020. Data collection in Supaul district was started in February 2020 and could not be continued due to the Covid-19 pandemic lockdown. Hence, the data available for analysis was for three rounds in Gaya district and for one round of data collection in Supaul district.

For peer review only - http://bmjopen.bmj.com/site/about/guidelines.xhtml

The permission to conduct the study was sought from the facility-in-charge. In each data collection round, exit interviews were conducted with the pregnant women. Interviewers trained in the study procedures contacted pregnant women after they registered at the out-patient department (OPD) registration for ANC services. Those who were able to provide a phone number were explained in brief about the purpose of the study and requested to wait after they had availed ANC services on that day. The team posted at the health facility kept track of these women through the various steps of ANC services, and re-contacted them after they had availed the services. They were provided refreshments and explained the purpose of the study. Those who consented were interviewed in a semi-private place in the facility premises. The exit interviews documented socio-demographic information of the pregnant woman, details of the ANC services provided on the given PMSMA day, women's knowledge of the services received and what they were informed post the check-up. In addition, the field staff placed in each facility observed the flow of ANC service provision being followed for the pregnant women on the given PMSMA day. The entire process was documented in a format specifically developed to track the process from the beginning till the end.

The study tools were developed in English and then translated into Hindi (local language), after which back translated into English to ensure accurate and relevant meaning and intent of the questions. Pilot testing of the questionnaire was carried out and modifications made as necessary. The interviews were captured using the CAPI software in handheld tablets. Data entered was scrutinised using the internal checks, and some portion of the 30% of all interviews were also attended by a supervisor for quality control purpose with prior permission taken from the respondent to do so.

Data analysis

We present the coverage of each ANC component by the pregnancy trimester, facility and district. Whether pregnant women have received some or all components of a set of interventions as part of ANC at least once during pregnancy has been used to indicate quality of care.[18-20] For this analysis, we defined quality of ANC services if the pregnant women received all of the following ANC service

For peer review only - http://bmjopen.bmj.com/site/about/guidelines.xhtml

components - weight measurement, blood pressure check-up, abdomen check-up, urine sample taken, blood sample taken, iron and folic acid and calcium tablets given - on that PMSMA day. Though the PMSMA is primarily designed to improve the coverage of quality of ANC services for women in 2nd and 3rd trimester of pregnancy, in reality, women in the 1st trimester also come to PMSMA day for ANC services. Therefore, we include these women in our analysis and present findings separately by pregnancy trimester; and report quality of ANC services for the women in 1st trimester of pregnancy with and without considering abdomen check-up. We report the coverage of quality of ANC services by select socio-demographic and pregnancy trimester, district and type of health facility. We did not include tetanus toxoid in this assessment as it's administration in pregnant women is dependent on certain factors.[21] The tetanus toxoid coverage for last pregnancy in Bihar was reported at 89.5% in the most recent state-wide assessment.[14] We report on whether the pregnant women were informed about the check-up provided to them and of the clinical findings by the health providers. We compared the steps followed in the ANC service provision in each facility against what is recommended in the PMSMA guidelines to highlight the issues in process that could have implications on the quality of service provision.[22] STATA 13.1 version was used for all analysis.

Patient and Public Involvement statement: Patients were not involved in planning of this research study.

For peer review only - http://bmjopen.bmj.com/site/about/guidelines.xhtml

RESULTS

A total of 961 pregnant women were identified of whom 861 (89.6%) were eligible for the study, and of whom 814 (94.5%) participated. Supplementary Table 1 in Supplementary file documents the distribution of pregnant women based on the pregnancy trimester. Many of the pregnant women were in 20-24 years' age group (59.5%), 711 (87.3%) women were in their 2nd or 3rd trimester of pregnancy, and women belonging to other than forward caste (88%) accounted for most of the sample across the facilities (Supplementary table 1 in Supplementary file).

Coverage of each ANC component

The component-wise coverage of ANC services provided to the pregnant women on the PMSMA day is shown in Figure 1 and Supplementary Table 2 in Supplementary file. Overall, weight check-up was reported by 98.4% (95% CI 97.3-99.1) and blood pressure check-up by 97.1% (95% CI 95.7-98.2) of the pregnant women (Figure 1). There was no significant difference in provision of these two components either by the pregnancy trimester or by the health facility (Figure 1). Though 80% of the women for whom weight was checked were also informed of the weight reading, less than one-third of them were informed about appropriateness of the weight gain as per the pregnancy trimester (Table 1). Of the 790 women for whom blood pressure was checked, only 463 (58.7%) were informed the blood pressure reading and of them only 223 (28.2%) were informed of the appropriateness of the reading (Table 1). There was considerable variation in women being informed about blood pressure by the pregnancy trimester (Table 1).

For peer review only

Table 1: Distribution of pregnant women based on the component-wise antenatal care service provided on the given day and information provided to pregnant women regarding each of those components. CI denotes confidence interval and NA denotes not applicable.

Service provided and women informed	All women N =814 (%)	Women in 1st trimester of pregnancy N=103 % (95% CI)	Women in 2nd trimester of pregnancy N=405 % (95% CI)	Women in 3rd trimester of pregnancy N=306 % (95% CI)
Weight checked	801 (98.4)	99<br>96.1 (89.9-98.5)	402<br>99.3 (97.7-99.7)	300<br>98.0 (95.7-99.1)
Informed the weight reading for whom weight was checked	708 (88.4)	82<br>82. 8 (74.0-89.1)	363<br>90.3 (86.9-92.8)	263<br>87.7 (83.4-90.9)
Informed about appropriateness	246 (30.7)	32	125	89

For peer review only - http://bmjopen.bmj.com/site/about/guidelines.xhtml

of weight gain by the stage of pregnancy for whom weight was checked		32.3 (23.8-42.2)	31.1 (26.7-35.8)	29.7 (24.7-35.1)
Blood pressure checked	790 (97.0)	99<br>96.1 (90.1-98.5)	395<br>97.5 (95.5-98.7)	296<br>96.7 (94.0-98.2)
Informed the blood pressure reading for whom blood pressure was checked	463 (58.7)	56<br>56.6 (46.6-66.0)	237<br>60.1 (55.2-64.9)	170<br>57.6 (51.9-63.2)
Informed about the appropriateness of blood pressure reading for whom blood pressure was checked	223 (28.2)	28<br>28.3 (20.2-38.0)	114<br>28.9 (24.6-33.5)	81<br>27.4 (22.6-32.7)
Blood sample taken	697 (85.6)	94<br>91.3 (83.9-95.4)	346<br>85.4 (81.6-88.5)	257<br>83.9 (79.4-87.7)
Informed that anaemia test will be done for whom blood sample was taken	87 (12.5)	13<br>13.8 (8.2-22.4)	43<br>12.4 (9.3-16.3)	31<br>12.1 (8.6-16.7)
Informed about the haemoglobin level for whom blood sample was taken	235 (33.7)	41<br>43.6 (33.9-53.8)	123<br>35.5 (30.7-40.7)	71<br>27.6 (22.5-33.4)
Informed about blood sugar level for whom blood sample was taken	19 (2.7)	1<br>1.1 (0.1-7.3)	12<br>3. 5 (2.0-6.0)	6<br>2.3 (1.0-5.1)
Abdomen checked	505 (62.0)	25<br>24.3 (16.9-33.5)	253<br>62.5 (57.6-67.1)	227<br>74.2 (69.0-78.8)
Explained the process to monitor baby movements for whom abdomen was checked	33 (7.7)	NA	19<br>9.4 (6.0-14.2)	15<br>6.6 (4.0-10.7)
IFA tablets given	736 (90.4)	86<br>83.5 (74.9-89.5)	375<br>92.6 (89.6-94.8)	275<br>89.8 (85.9-92.8)
Informed about the benefits and side-effects to whom IFA tablets were given	193 (26.2)	19<br>23.5 (15.4-34.0)	91<br>26.2 (21.8-31.1)	83<br>31.6 (26.2-37.4)
Calcium tablets given	750 (92.1)	88<br>85.4 (77.2-91.1)	383<br>94.6 (91.9-96.4)	279<br>91.2 (87.8-94.1)
Informed about benefits to whom calcium tablets were given	114 (15.6)	8<br>9.3 (4.7-17.6)	61<br>16.4 (13.0-20.6)	45<br>16.6 (12.6-21.5)

For peer review only

The urine sample collection coverage was 46.3% (95% CI 42.9-49.7) with significant variations seen by district, pregnancy trimester and facility (Figure 1). The blood sample collection coverage was 85.6% (95% CI 83.0-87.8), with no significant variation by pregnancy trimester (Figure 1). Of the 697 women for whom blood sample was taken, only 235 (33.7%) were informed about the haemoglobin value, and almost none were informed about the blood sugar value (Table 1).

The coverage of abdomen check-up was 62% (95% CI 58.6-65.3) and significant variations were seen by the pregnancy trimester and health facility (Figure 1). Only 7.7% women of the women in 2nd or 3rd trimester were explained how to monitor baby's movements (Table 1). Overall 90.4% (95% CI 88.2-92.2) of the pregnant women reported receiving IFA tablets and 92.1% (95% CI 90.1-93.8) receiving calcium tablets (Figure 1). No significant variations were observed by the pregnancy trimester or health facility for both receipt of the IFA and calcium tablets (Figure 1). Of the 736 women who had received IFA tablets, only 193 (26.2%) were informed about the benefits and side effects of it. Only 114 (15.6%) women of the 750 women who had received calcium tablets were informed about the benefits of it (Table 1).

Quality of ANC services

The coverage of quality of ANC services on considering abdomen check-up for all women irrespective of the pregnancy trimester was 30.4% (95% CI 27.3-33.7) and was similar in both the districts (Figure 2A). This coverage was significantly lower for women in the 1st trimester of pregnancy (6.8%, 95% CI 3.3-13.6) as compared with those in the 2nd or 3rd trimester of pregnancy (Figure 2A). The coverage varied significantly between the facilities, and ranged from 3-66% in district 1 and 4.5-83.1% in district 2 (Figure 2A). The coverage of quality of ANC services was similar by maternal age and caste (Supplementary table 2 in Supplementary file).

On not considering the abdomen check-up for women in the 1st trimester of pregnancy (Figure 2B), the coverage of quality of ANC services for women in 1st trimester of pregnancy increased to 28.1% (95% CI 20.3- 37.6), and the overall coverage of quality of ANC services increased only slightly to 33.1% (Figure 2B). The results of coverage of quality of ANC services by maternal age

For peer review only - http://bmjopen.bmj.com/site/about/guidelines.xhtml

and caste when abdomen check-up was not considered for the women in 1st trimester of pregnancy were similar to those with consideration (Supplementary table 2 in Supplementary file).

Among the 306 pregnant women who were in their 3rd trimester of pregnancy (Supplementary table 1 in Supplementary file), for 64 (20.9%) women the study visit was their 1st ANC visit, for 95 (31.0%) women it was their 2nd or 3rd ANC visit, and for 147 (48%) women it was their 4th ANC visit or more. Not much variation was seen in the coverage of individual ANC component based on the number of ANC visit that they were in for (Supplementary Figure 1 in Supplementary file). The coverage of quality of ANC services was 34.4%, 26.3%, and 36.1% for women who had come for their 1st, 2nd or 3rd, 4th visit or more ANC visit (Supplementary Figure 1 in Supplementary file).

Process of ANC services

The number and cadre of staff available for ANC services on the PMSMA day ranged from 6 to 13 across the various rounds of data collection in the two districts (Supplementary table 3 in Supplementary file). The average number of pregnant women examined per doctor (range 25.5 to 118), ANM (range 10.3 to 44.5) and lab technician (range 20.5 to 122) varied significantly across the facilities and rounds of data collection. The process as per the PMSMA guidelines and what was observed in the facilities is shown in Table 2. In all the facilities, pregnant women registered for ANC check-up at the registration counter and were given “Out-patient department (OPD) slip” with a number. This OPD number was relevant only for that day and no facility had a system of tracking if a woman had visited for ANC services earlier. As per the PMSMA guidelines, step 2 is medical check-up done by ANMs followed by laboratory investigations (step 3), collection of laboratory reports (step 4), and with this the pregnant women are seen by the doctor (step 5). However, such a process was not observed in any facility (Table 2).

For peer review only - http://bmjopen.bmj.com/site/about/guidelines.xhtml

Table 2: Process of antenatal care services observed in the study facilities. CHC denotes Community Health Centre and SDH denotes Sub-district Hospital.

Steps suggested in the PMSMA guidelines		Steps followed at the sampled facilities					
**Step**	**Detail**	**Gaya district CHC 1**	**Gaya district CHC 2**	**Gaya district SDH**	**Supaul district CHC 1**	**Supaul district CHC 2**	**Supaul district SDH**
1	Registration	Registration	Registration	Registration	Registration	Registration	Registration
2	ANC OPD by ANM/Nurse	Doctor's consultation	Doctor's consultation	Doctor's consultation	Receipt of medicines	Laboratory investigations	ANC OPD by ANM/Nurse
3	Laboratory investigations	Laboratory investigations	Laboratory investigations	Laboratory investigations	Laboratory investigations	Collection of laboratory reports	Doctor's consultation
4	Collection of laboratory reports	Collection of laboratory reports	Collection of laboratory reports	Collection of laboratory reports	Collection of laboratory reports	ANC OPD by ANM/Nurse	Laboratory investigations
5	Doctor's consultation	ANC OPD by ANM/Nurse	ANC OPD by ANM/Nurse	ANC OPD by ANM/Nurse	Doctor's consultation	Doctor's consultation	Collection of laboratory reports
6	Counselling	Receipt of medicines	Receipt of medicines	Receipt of medicines	ANC OPD by ANM/Nurse	Receipt of medicines	Counselling
7	Receipt of medicines				Counselling		Receipt of medicines
8	Feedback and grievance redressal				Feedback and grievance redressal		Feedback and grievance redressal

For peer review only - http://bmjopen.bmj.com/site/about/guidelines.xhtml

Doctor consultation (step 5) was done before the steps 2-4 in 4 of the 6 facilities. Of the 22 doctors who provided ANC services in these facilities across all the rounds, 14 (63.6%) were males; 11 (50%) were MBBS, 2 (9.1 %) were MD, 4 (18.2 %) were dental specialist, 5 (22.7%) were alternate medicine doctors. A total of 349 (65.9%) and 151 (58.9%) women in Gaya and Supaul districts were seen by a doctor, respectively. The doctors were observed mainly handing over prescription indicating blood and urine tests to be done, and the list of medicines to be provided to the pregnant women in all facilities except one where the doctor performed medical check-up.

Urine sample was taken only for 276 (50.3%) and 101 (38.1%) women in Gaya and Supaul districts, respectively. In some facilities, women were given new containers for urine sample collection and in some old containers, and all tests were done only using dipstick. No facility had storage option available for urine samples. The laboratory technician noted name and serial number for each pregnant woman in the laboratory register, and indicated the same number on the container. Women were asked to go to the toilet with the container and dipstick, to dip the dipstick one inch in the urine, and bring it back to the technician. No laboratory technician was seen handling the dipstick; women were asked to hold it up and the technician recorded the reading from a distance. The field team noticed make-shift toilet (Supplementary Figure 2 in Supplementary file) in one facility and the toilet in one facility was on another floor than the ANC OPD. Pregnant women were seen hesitating in using both of these toilets, and some did not take the urine test even after being given the container. In the remaining facilities, pregnant women had to use a toilet elsewhere which was not close to the laboratory. Some were noticed being embarrassed at carrying the container and dipstick as the other people present in the facility could see them. Blood sample was taken only for 437 (79.6%) and 260 (98.1%) women in Gaya and Supaul districts, respectively. Venous blood was drawn for 250 (35.9%) and the rest was finger prick method. The laboratory technicians were not seen wearing gloves every time they took a blood sample. Blood samples were discarded immediately after recording of the haemoglobin value in all facilities.

For peer review only

For peer review only - http://bmjopen.bmj.com/site/about/guidelines.xhtml

Almost all the check-up that the pregnant women were offered was done by the ANMs in all the facilities. None of the clinical findings by the ANMs were checked by the doctor as the women were made to see the doctor before any check-up was done. In all facilities, the ANMs entered the clinical data in the PMSMA register next day and not immediately after examining the women. The counselling services (step 6) and feedback redressal (step 7) were seen only in 2 of the 6 facilities. Only 38 (18.3%) and 11 (11.8%) of the women in SDH of Gaya and Supaul districts reported meeting a counsellor, respectively.

For peer review only

For peer review only - http://bmjopen.bmj.com/site/about/guidelines.xhtml

DISCUSSION

In our understanding, this is the first prospective study undertaken in health facility in a developing country setting that provides a systematic documentation of quality of ANC services considering all the ANC components through patient exit survey, along with documentation of the process of ANC service provision. Only one-third of the pregnant women had received quality ANC services under the PMSMA, and even less were informed about the services that they had received. The wide variations in the implementation of PMSMA at the facilities, and the task shifting for service provision will have to be addressed urgently to improve the quality of ANC service provision.

Weight and blood pressure measurements, and provision of IFA and calcium tablets showed consistently high coverage across all the facilities irrespective of the pregnancy trimester. Most variation was seen in the coverage of urine examination followed by abdomen check-up. The laboratory infrastructure and related processes, and the laboratory technicians as human resources for health have not received the necessary attention in provision of health services, including in maternal and child health services.[23-25] This study has highlighted that good clinical laboratory practice guidelines were not followed for urine examination in any of the facilities.[26] Notably, though the coverage of blood sample examination was relatively higher than that for urine, however, only blood sugar and anaemia levels were recorded and none of the other tests recommended in pregnancy were performed. These findings suggest that provision of high-quality ANC care is unlikely without improving the laboratory readiness.[27] Laboratory services have recently received unprecedented attention in India due to Covid-19, and it would be important to sustain this and expand it to routine laboratory tests as well.[28 29]

The Indian ANC guidelines recommend abdomen check-up from second pregnancy trimester onwards to monitor pregnancy progression, foetal well-being and its position.[27] We have previously reported from this population that breech position of the baby is not known to most women before delivery as abdomen check-up is either not done or they are not informed, and breech position is a significant risk factor for early neonatal deaths and stillbirths in this population.[30 31] Abnormal foetal

For peer review only - http://bmjopen.bmj.com/site/about/guidelines.xhtml

movements can be used to predict adverse neonatal outcomes,[32-34] however, only 1 in 12 women who had received abdomen check-up was informed about monitoring of baby movements in this study.

The wide range of staff available for PMSMA service delivery between and within the facilities over rounds of data collection, and the substantial variations found in the steps of PMSMA implementation at the facilities are of concern with regards to the provision of quality services. Almost all the check-up was provided by ANMs in all the facilities despite the premise of PMSMA day being that every pregnant woman in India is examined by a physician and appropriately investigated at least once during the PMSMA .[12] Notably, the doctor's consultation did not involve any examination of the pregnant women in all but one facility even though the availability of doctors was not an issue. These findings indicate task shifting which is neither in line with the PMSMA guidelines[12] nor with the task shifting recommendations to maintain the quality of services.[35] Importantly, the program implementation needs to address and account for such task shifting to address the poor quality of ANC services. The wide variation in coverage of quality indicates the scope for improvement. Raising the performance of all facilities to the level of best performance should be feasible with more in-depth understanding of implementation at the facility level, which would lead to significant overall gains in quality and ensure that pregnant women receive quality services irrespective of the facility they access for these services.[7]

Even though the weight and blood pressure measurements, blood sample collection, and provision of IFA and calcium tablets showed high coverage, less than one-third of the pregnant women were informed about the findings or implications of these for pregnancy. This coupled with almost non-existence of counselling is a major concern. Low birthweight is reported to be the largest contributor to child malnutrition disability-adjusted-life years in India,[36] and birthweight is an intergenerational issue dependent on an interplay of various factors, including maternal undernutrition and intrauterine growth. Also, anaemia increases the risk of adverse birth outcomes, and the high burden of anaemia in Indian women has not declined since 1990.[36] With very poor

For peer review only - http://bmjopen.bmj.com/site/about/guidelines.xhtml

communication with the women and almost no counselling, pregnant women are not empowered to take correct steps for a positive pregnancy experience and to prevent adverse birth outcomes.[37]

Substantial inequities in coverage of quality of ANC services by socio-economic status have been highlighted in the previous assessments.[1 38] As this study was carried out only in the public sector facilities, we are unable to comment on inequities in general. However, it is important to note that the coverage of quality of ANC services in our study was similar based on caste of women, which is a surrogate measure of socio-economic status in rural India.

This study was conducted in six facilities only, which could be considered as a limitation to generalise these findings. It is important to note that concerns have also been raised with the quality of maternal services in private sector, and it would be important to assess quality of ANC services for these facilities as well.[39-42] Furthermore, we were able to conduct data collection over only one instead of three rounds in one district. However, the study findings corroborate with previous findings of poor coverage of quality of ANC services in household surveys in the state despite the less number of rounds. Only 10% of women of the women were excluded because of non-availability of a phone for contact later for follow-up assessment. There are several strengths of this study. There is almost no recall bias in the information reported by the respondents as this was an exit survey. The prospective nature of this study, assessment of ANC services components beyond what is previously reported, documentation of what women are informed and the process of ANC services are strengths of this undertaking.

The new World Health Organization ANC model recommends that women attend a minimum of eight ANC visits.[37] However, with the significantly poor levels of quality of ANC services as documented in this study and previously, focussing only on increasing the number of visits is unlikely to produce the desired maternal and neonatal health outcomes.[43] With coverage of quality of ANC services at 30% on PMSMA day, the effort to improve quality of ANC can go only thus far unless the quality of ANC is explicitly tracked and monitored through standard indicators both at the health system and community levels.[43-45] The India Newborn Action Plan recommends

For peer review only - http://bmjopen.bmj.com/site/about/guidelines.xhtml

monitoring of percentage of pregnant women who received full ANC but the definition of full ANC is not provided.[46] In conclusion, the findings suggest that in order to provide services as envisaged under the PMSMA, there is a need to cultivate quality of ANC services at the public health facilities; train doctors, ANMs and laboratory technicians to communicate with the pregnant women; and build clinical and technical capacity and supervision to train health providers to follow the standard guidelines for provision of quality ANC services.

For peer review only

For peer review only - http://bmjopen.bmj.com/site/about/guidelines.xhtml

Ethics approval: This study was approved by the Ethics Committee of the Public Health Foundation of India (TRC-IEC 410.1/19).

Authors' contributions: RD and GAK had full access to data in the study, take full responsibility for the integrity of data and accuracy of the data analysis, and had final responsibility for the decision to submit for publication; RD, GAK and LD conceptualized the study; RD guided the data analysis and drafted the manuscript; MM and GAK performed data analysis; MA guided data collection; MA, DB, and PN contributed to data analysis and interpretation; LD contributed to drafting of the manuscript and interpretation; all authors approved the final manuscript.

Funding: Bill and Melinda Gates Foundation; grant number INV-007989.

Declaration of interests: DB and PN are employees of the Bill & Melinda Gates Foundation, India Country Office, New Delhi. The other authors have no conflict of interest.

Data availability statement sharing: All data generated or analysed during this study are included in this published article and its supplementary information files.

Acknowledgements: The authors acknowledge the contributions of Sibin George and S Siva Prasad from Public Health Foundation of India, and Asif Iqbal and Vipul Singhal from the Oxford Policy Management, India for data collection and data management.

For peer review

REFERENCES

1. Arsenault C, Jordan K, Lee D, et al. Equity in antenatal care quality: an analysis of 91 national household surveys. *Lancet Glob Health* 2018;6(11):e1186-e95. doi: 10.1016/s2214-109x(18)30389-9 [published Online First: 2018/10/17]
2. Chukwuma A, Wosu AC, Mbachu C, et al. Quality of antenatal care predicts retention in skilled birth attendance: a multilevel analysis of 28 African countries. *BMC Pregnancy Childbirth* 2017;17(1):152. doi: 10.1186/s12884-017-1337-1 [published Online First: 2017/05/27]
3. Yeoh PL, Hornetz K, Shauki NIA, et al. Evaluating the quality of antenatal care and pregnancy outcomes using content and utilization assessment. *Int J Qual Health Care* 2018;30(6):466-71. doi: 10.1093/intqhc/mzy041 [published Online First: 2018/03/29]
4. Sommer Albert J, Younas A, Victor G. Quality of Antenatal Care Services in a Developing Country: A Cross-Sectional Survey. *Creat Nurs* 2020;26(1):e25-e34. doi: 10.1891/1078-4535.26.1.e25 [published Online First: 2020/02/07]
5. Wynne SJ, Duarte R, de Wildt G, et al. The timing and quality of antenatal care received by women attending a primary care centre in Iquitos, Peru: A facility exit survey. *PLoS One* 2020;15(3):e0229852. doi: 10.1371/journal.pone.0229852 [published Online First: 2020/03/07]
6. Gwako GN, Were F, Obimbo MM, et al. Association between utilization and quality of antenatal care with stillbirths in four tertiary hospitals in a low-income urban setting. *Acta Obstet Gynecol Scand* 2021;100(4):676-83. doi: 10.1111/aogs.13956 [published Online First: 2020/07/11]
7. Phommachanh S, Essink DR, Jansen M, et al. Improvement of Quality of Antenatal Care (ANC) Service Provision at the Public Health Facilities in Lao PDR: Perspective and Experiences of Supply and Demand Sides. *BMC Pregnancy Childbirth* 2019;19(1):255. doi: 10.1186/s12884-019-2345-0 [published Online First: 2019/07/25]
8. Macarayan EK, Gage AD, Doubova SV, et al. Assessment of quality of primary care with facility surveys: a descriptive analysis in ten low-income and middle-income countries. *Lancet Glob Health* 2018;6(11):e1176-e85. doi: 10.1016/s2214-109x(18)30440-6 [published Online First: 2018/10/17]
9. Singh L, Dubey R, Singh S, et al. Measuring quality of antenatal care: a secondary analysis of national survey data from India. *BJOG* 2019;126 Suppl 4:7-13. doi: 10.1111/1471-0528.15825 [published Online First: 2019/05/28]
10. Munuswamy S, Nakamura K, Seino K, et al. Inequalities in Use of Antenatal Care and Its ServiceComponents in India. *J Rural Med* 2014;9(1):10-9. doi: 10.2185/jrm.2877 [published Online First: 2015/02/05]
11. Bucher S, Marete I, Tenge C, et al. A prospective observational description of frequency and timing of antenatal care attendance and coverage of selected interventions from sites in Argentina, Guatemala, India, Kenya, Pakistan and Zambia. *Reprod Health* 2015;12 Suppl 2(Suppl 2):S12. doi: 10.1186/1742-4755-12-s2-s12 [published Online First: 2015/06/13]
12. Ministry of Health and Family Welfare GoI. Pradhan Mantri Surakshit Matritva Abhiyan [updated 15 March 2021. Available from: https://pmsma.nhp.gov.in/about-scheme/#about accessed 26 May 2021.
13. Registrar General of India. C.D. Blockwise Primary Census Abstract Data (PCA) - Bihar New Delhi, India2011 [Available from: http://censusindia.gov.in/pca/cdb_pca_census/Houselisting-housing-BR.html accessed 21 May 2018.
14. International Institute for Population Sciences. National Family Healty Survey (NFHS-5): Bihar. Mumbai: IIPS, 2020.
15. India State-Level Disease Burden Initiative Child Mortality Collaborators. Subnational mapping of under-5 and neonatal mortality trends in India: the Global Burden of Disease Study 2000-17. *Lancet* 2020;395(10237):1640-58. doi: 10.1016/s0140-6736(20)30471-2 [published Online First: 2020/05/16]

For peer review only - http://bmjopen.bmj.com/site/about/guidelines.xhtml

16. Institute for Health Metrics and Evaluation (IHME). Maternal Health Atlas Seattle, WA: IHME, University of Washington; 2018 [Available from: https://maternalhealthatlas.org/ accessed 28 May 2021.

17. International Institute for Population Sciences. National Family Healty Survey (NFHS-4) India 2015-16: Bihar Mumbai: IIPS; 2017 [Available from: http://rchiips.org/nfhs/NFHS-4Reports/Bihar.pdf accessed 18 June 2021.

18. Abou Zahr CWTMWHOU. Antenatal care in developing countries : promises, achievements, and missed opportunities : an analysis of trends, levels, and differentials, 1990-2001. Geneva; New York: World Health Organization ; UNICEF 2003.

19. Carvajal-Aguirre L, Amouzou A, Mehra V, et al. Gap between contact and content in maternal and newborn care: An analysis of data from 20 countries in sub-Saharan Africa. *Journal of global health* 2017;7(2):020501. doi: 10.7189/jogh.07.020501 [published Online First: 2018/02/10]

20. Kyei NNA, Chansa C, Gabrysch S. Quality of antenatal care in Zambia: a national assessment. *BMC Pregnancy Childbirth* 2012;12:151-51. doi: 10.1186/1471-2393-12-151

21. World Health Organization. Standards for Maternal and Neonatal Care : Maternal immunization against tetanus. In: Safe DoMP, ed. Geneva: WHO.

22. Ministry of Health and Family Welfare GoI, . Pradhan Mantri Surakshit Matritva Abhiyan New Delhi, 2016.

23. Sayed S, Cherniak W, Lawler M, et al. Improving pathology and laboratory medicine in low-income and middle-income countries: roadmap to solutions. *Lancet* 2018;391(10133):1939-52. doi: 10.1016/s0140-6736(18)30459-8 [published Online First: 2018/03/20]

24. Horton S, Sullivan R, Flanigan J, et al. Delivering modern, high-quality, affordable pathology and laboratory medicine to low-income and middle-income countries: a call to action. *Lancet* 2018;391(10133):1953-64. doi: 10.1016/s0140-6736(18)30460-4 [published Online First: 2018/03/20]

25. Karan A, Negandhi H, Hussain S, et al. Size, composition and distribution of health workforce in India: why, and where to invest? *Hum Resour Health* 2021;19(1):39. doi: 10.1186/s12960-021-00575-2 [published Online First: 2021/03/24]

26. Indian Council of Medical Research. ICMR guidelines for good clinical laboratory practices (GCLP) 2021. New Delhi: ICMR, 2021.

27. Ministry of Health and Family Welfare GoI, . Guidelines for antenatal care and skilled attendance at birth by ANMs/LHVs/SNs. In: Division MH, ed. New Delhi: GoI, 2010.

28. Indian Council of Medical Research. Information for testing laboratories: ICMR; 2021 [Available from: https://www.icmr.gov.in/ctestlab.html accessed 2 June 2021.

29. The Federation of Obstetric and Gynaecological Societies of India. FOGSI Good Clinical Practice recommendation on pregnancy with Covid-19. In: Gandhi A GA, Tank P., ed.: FOGSI, 2020.

30. Dandona R, Kumar GA, Akbar M, et al. Deferred and referred deliveries contribute to stillbirths in the Indian state of Bihar: results from a population-based survey of all births. *BMC Med* 2019;17(1):28. doi: 10.1186/s12916-019-1265-1 [published Online First: 2019/02/08]

31. Dandona R, Kumar GA, Bhattacharya D, et al. Distinct mortality patterns at 0-2 days versus the remaining neonatal period: results from population-based assessment in the Indian state of Bihar. *BMC Med* 2019;17(1):140. doi: 10.1186/s12916-019-1372-z [published Online First: 2019/07/20]

32. Scala C, Bhide A, Familiari A, et al. Number of episodes of reduced fetal movement at term: association with adverse perinatal outcome. *Am J Obstet Gynecol* 2015;213(5):678 e1-6. doi: 10.1016/j.ajog.2015.07.015 [published Online First: 2015/07/25]

33. Dutton PJ, Warrander LK, Roberts SA, et al. Predictors of poor perinatal outcome following maternal perception of reduced fetal movements--a prospective cohort study. *PLoS One* 2012;7(7):e39784. doi: 10.1371/journal.pone.0039784 [published Online First: 2012/07/19]

For peer review only

For peer review only - http://bmjopen.bmj.com/site/about/guidelines.xhtml

34. Huang C, Han W, Fan Y. Correlation study between increased fetal movement during the third trimester and neonatal outcome. *BMC Pregnancy Childbirth* 2019;19(1):467. doi: 10.1186/s12884-019-2637-4 [published Online First: 2019/12/06]
35. World Health Organization P, and UNAIDS.,. Task shifting : rational redistribution of tasks among health workforce teams : global recommendations and guidelines. Geneva: WHO, PEPFAR, and UNAIDS, 2008.
36. India State-Level Disease Burden Initiative Malnutrition Collaborators. The burden of child and maternal malnutrition and trends in its indicators in the states of India: the Global Burden of Disease Study 1990-2017. *Lancet Child Adolesc Health* 2019;3(12):855-70. doi: 10.1016/s2352-4642(19)30273-1 [published Online First: 2019/09/23]
37. World Health Organization. WHO recommendations on antenatal care for a positive pregnancy experience. Geneva: WHO, 2017.
38. Sharma J, Leslie HH, Kundu F, et al. Poor Quality for Poor Women? Inequities in the Quality of Antenatal and Delivery Care in Kenya. *PLoS One* 2017;12(1):e0171236. doi: 10.1371/journal.pone.0171236 [published Online First: 2017/02/01]
39. Gautham M, Bruxvoort K, Iles R, et al. Investigating the nature of competition facing private healthcare facilities: the case of maternity care in Uttar Pradesh, India. *Health Policy Plan* 2019;34(6):450-60. doi: 10.1093/heapol/czz056 [published Online First: 2019/07/16]
40. Karvande S, Sonawane D, Chavan S, et al. What does quality of care mean for maternal health providers from two vulnerable states of India? Case study of Bihar and Jharkhand. *J Health Popul Nutr* 2016;35:6. doi: 10.1186/s41043-016-0043-3 [published Online First: 2016/02/22]
41. Tripathi S, Srivastava A, Memon P, et al. Quality of maternity care provided by private sector healthcare facilities in three states of India: a situational analysis. *BMC Health Serv Res* 2019;19(1):971. doi: 10.1186/s12913-019-4782-x [published Online First: 2019/12/18]
42. Zamboni K, Singh S, Tyagi M, et al. Effect of collaborative quality improvement on stillbirths, neonatal mortality and newborn care practices in hospitals of Telangana and Andhra Pradesh, India: evidence from a quasi-experimental mixed-methods study. *Implement Sci* 2021;16(1):4. doi: 10.1186/s13012-020-01058-z [published Online First: 2021/01/09]
43. Hodgins S, D'Agostino A. The quality-coverage gap in antenatal care: toward better measurement of effective coverage. *Glob Health Sci Pract* 2014;2(2):173-81. doi: 10.9745/ghsp-d-13-00176 [published Online First: 2014/10/03]
44. Rios-Zertuche D, Zúñiga-Brenes P, Palmisano E, et al. Methods to measure quality of care and quality indicators through health facility surveys in low- and middle-income countries. *Int J Qual Health Care* 2019;31(3):183-90. doi: 10.1093/intqhc/mzy136 [published Online First: 2018/06/20]
45. Morón-Duarte LS, Ramirez Varela A, Segura O, et al. Quality assessment indicators in antenatal care worldwide: a systematic review. *Int J Qual Health Care* 2019;31(7):497-505. doi: 10.1093/intqhc/mzy206 [published Online First: 2018/10/09]
46. Ministry of Health and Family Welfare, Government of India. INAP: India Newborn Action Plan. New Delhi: Government of India, 2014.

For peer review only - http://bmjopen.bmj.com/site/about/guidelines.xhtml

520 **FIGURE LEGENDS**

521 **Figure 1.** Coverage of ANC service components among the pregnant women on the PMSMA day.
522 Bars denote 95% confidence interval, CHC Community Health Centre and SDH Sub-district hospital.

523

524 **Figure 2.** Coverage of quality of ANC services among the pregnant women on the PMSMA day. Bars
525 denote 95% confidence interval, CHC Community Health Centre and SDH Sub-district hospital.

526

For peer review only

For peer review only - http://bmjopen.bmj.com/site/about/guidelines.xhtml

Figure 1: Coverage of ANC service components among the pregnant women on the PMSMA day. Bars denote 95% confidence interval, CHC Community Health Centre and SDH Sub-district hospital.

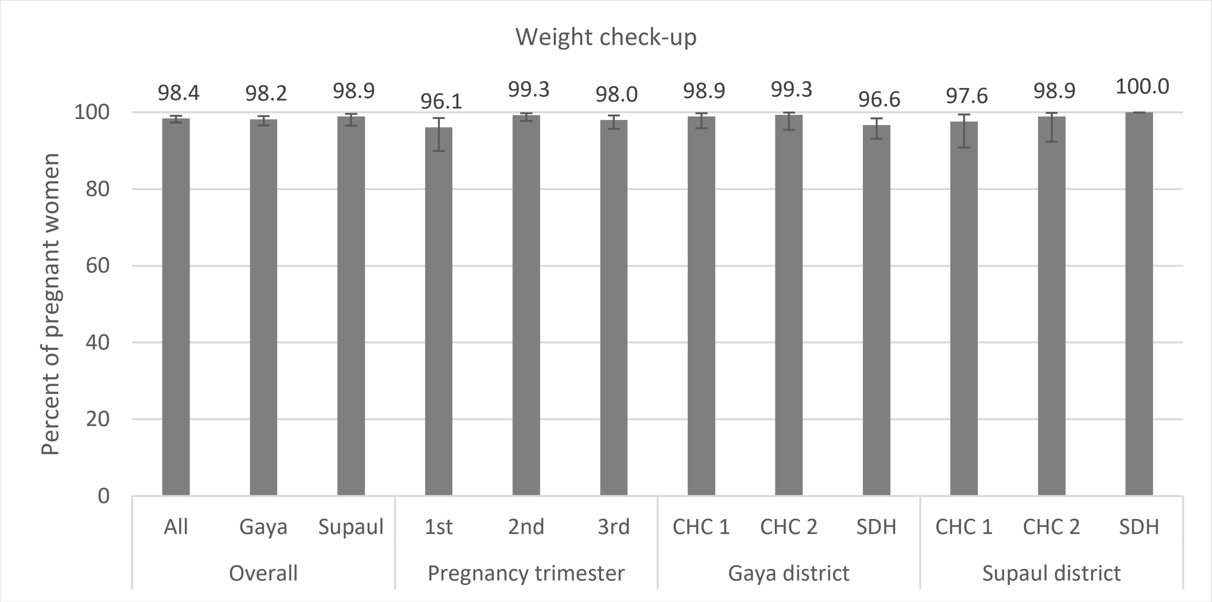

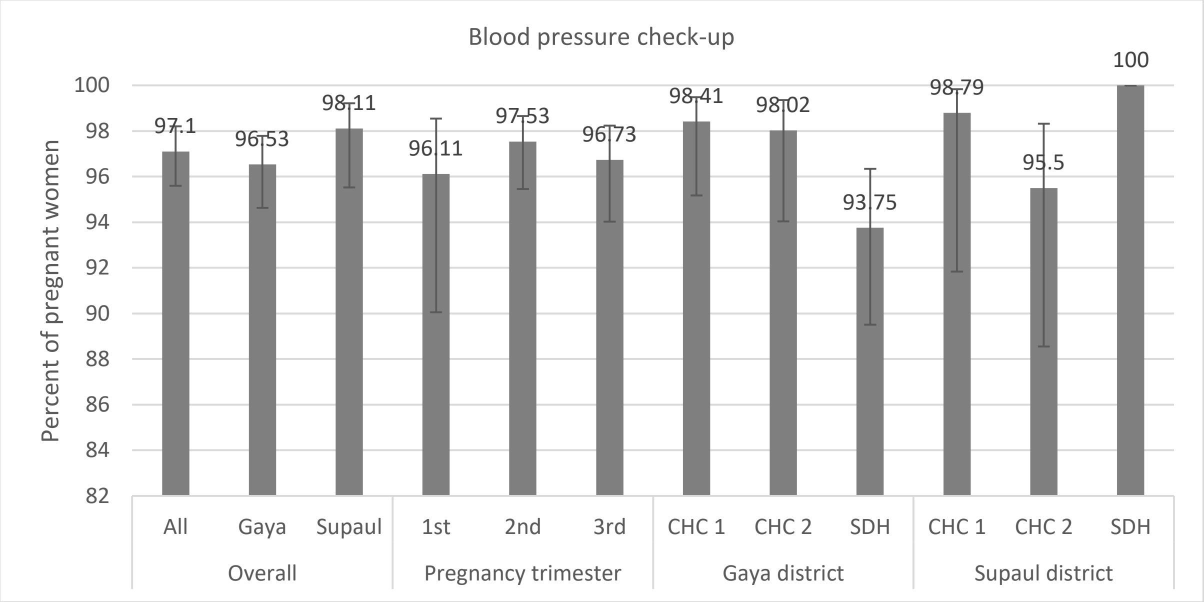

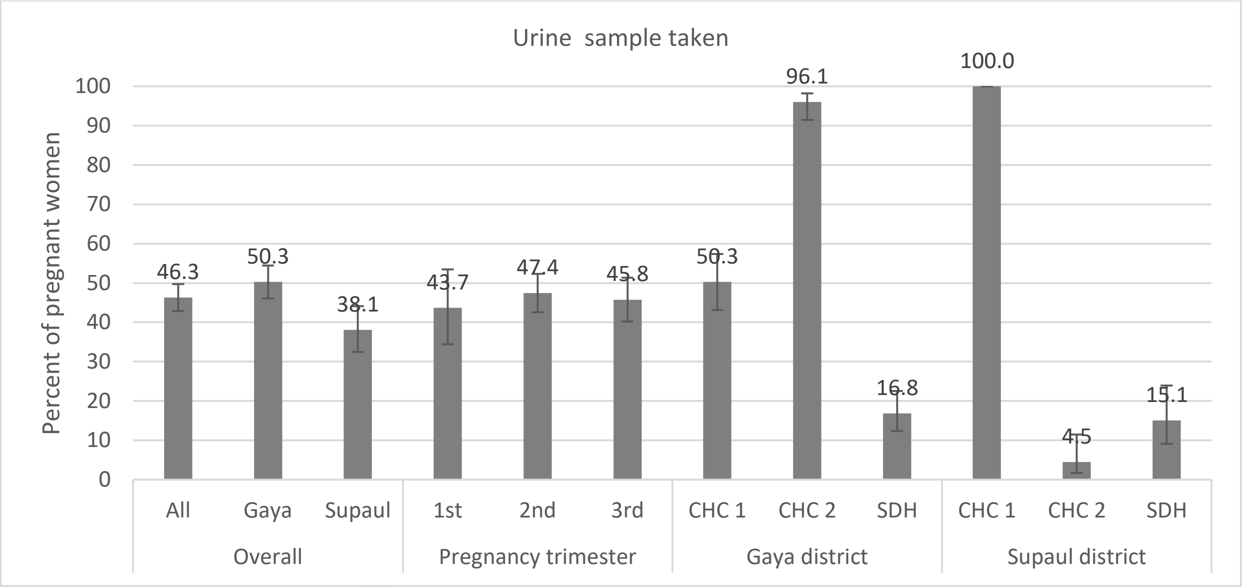
Urine sample taken
Percent of pregnant women
100
90
80
70
60
50
40
30
20
10
0
46.3
50.3
38.1
43.7
47.4
45.8
50.3
96.1
16.8
100.0
4.5
15.1
All
Gaya
Supaul
1st
2nd
3rd
CHC 1
CHC 2
SDH
CHC 1
CHC 2
SDH
Overall
Pregnancy trimester
Gaya district
Supaul district

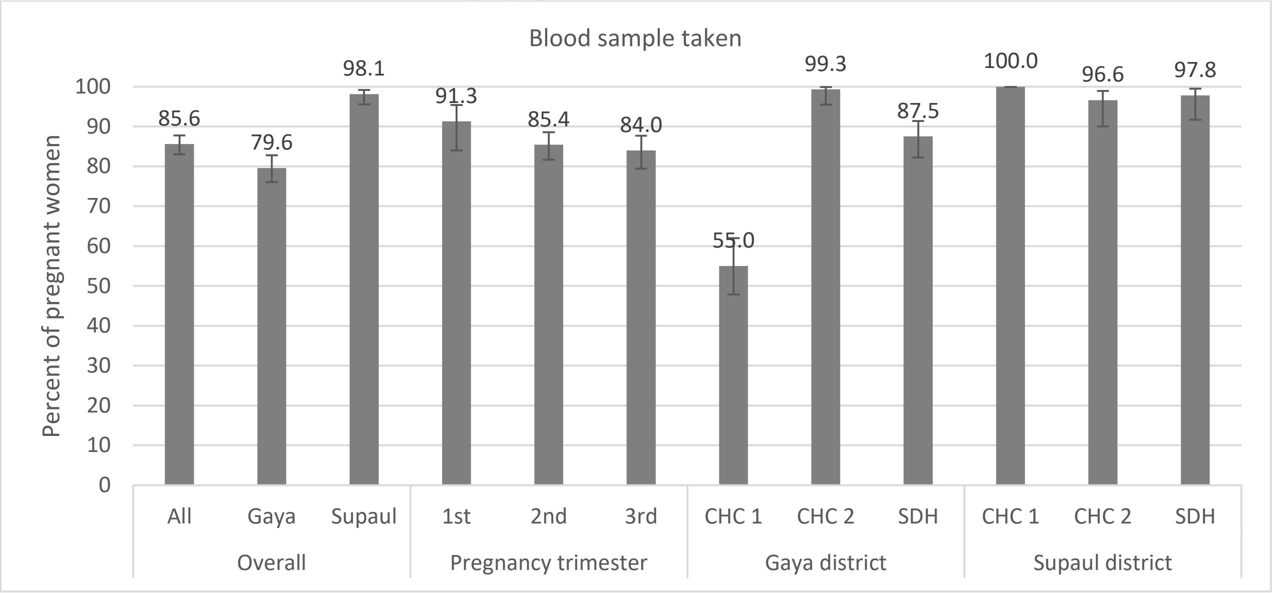
Blood sample taken
Percent of pregnant women
100
90
80
70
60
50
40
30
20
10
0
85.6
79.6
98.1
91.3
85.4
84.0
55.0
99.3
87.5
100.0
96.6
97.8
All
Gaya
Supaul
1st
2nd
3rd
CHC 1
CHC 2
SDH
CHC 1
CHC 2
SDH
Overall
Pregnancy trimester
Gaya district
Supaul district

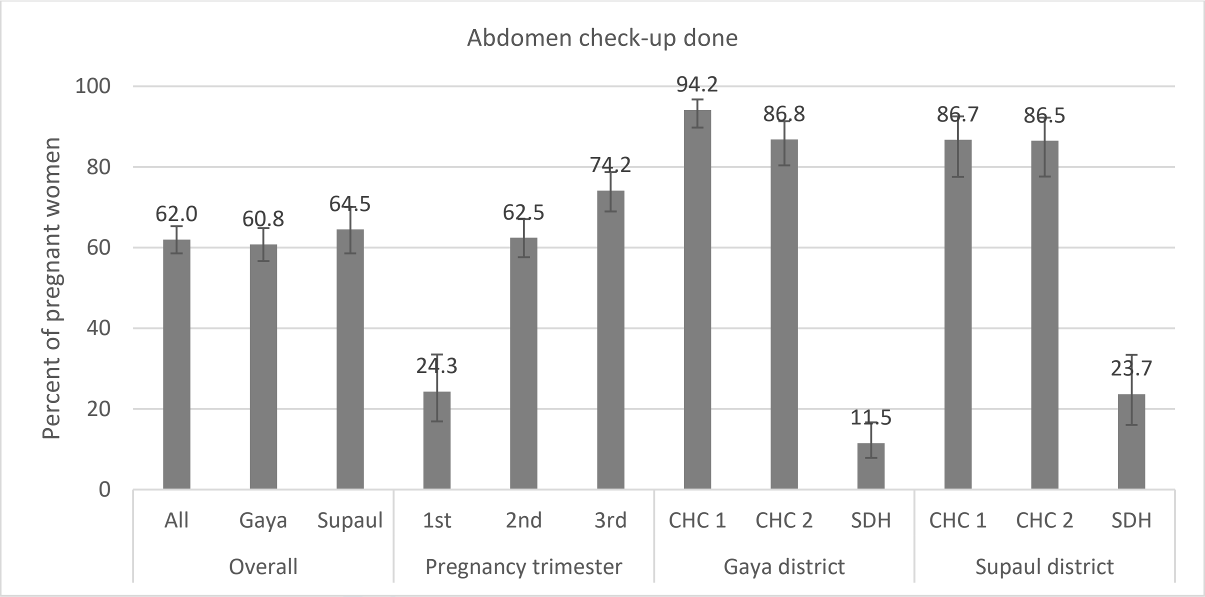
Abdomen check-up done
Percent of pregnant women
100
80
60
40
20
0
62.0
60.8
64.5
24.3
62.5
74.2
94.2
86.8
11.5
86.7
86.5
23.7
All
Gaya
Supaul
1st
2nd
3rd
CHC 1
CHC 2
SDH
CHC 1
CHC 2
SDH
Overall
Pregnancy trimester
Gaya district
Supaul district

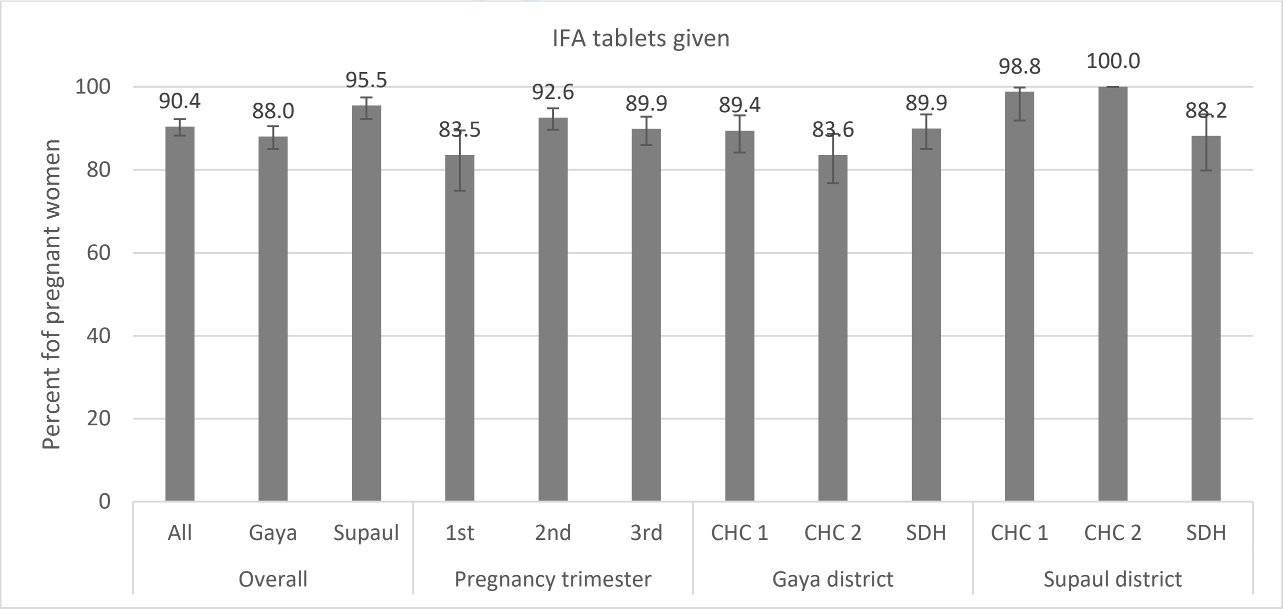
IFA tablets given
Percent fof pregnant women
100
80
60
40
20
0
90.4
88.0
95.5
83.5
92.6
89.9
89.4
83.6
89.9
98.8
100.0
88.2
All
Gaya
Supaul
1st
2nd
3rd
CHC 1
CHC 2
SDH
CHC 1
CHC 2
SDH
Overall
Pregnancy trimester
Gaya district
Supaul district

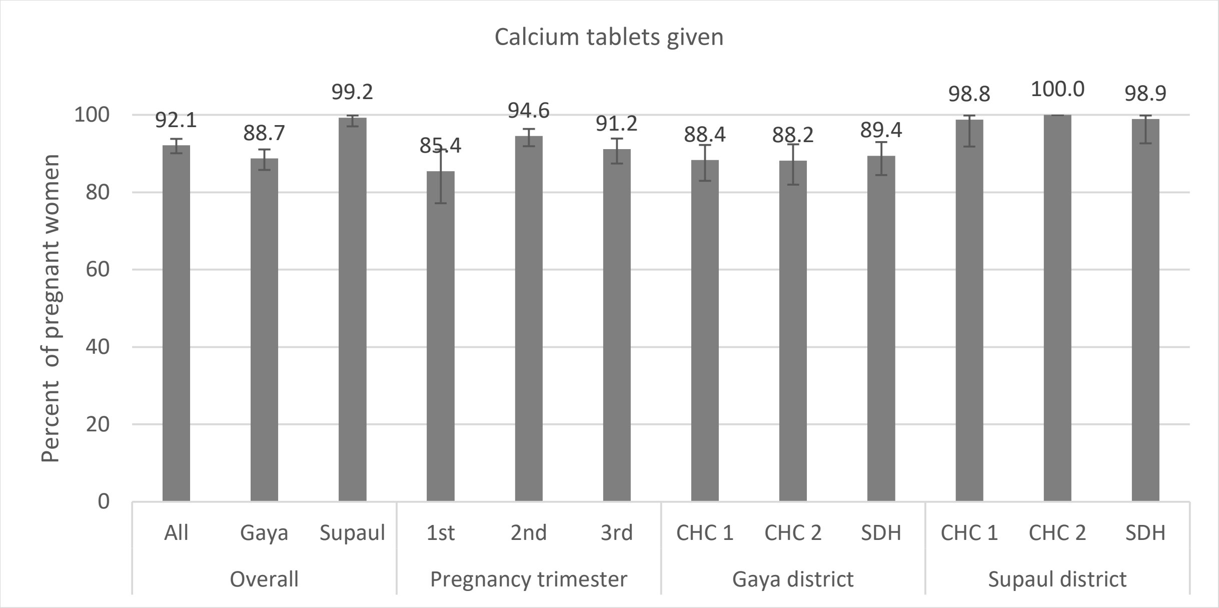

For peer review only

Figure 2: Coverage of quality of ANC services among the pregnant women on the PMSMA day. Bars denote 95% confidence interval, CHC Community Health Centre and SDH Sub-district hospital.

A. Considering abdomen check-up for women in 1st trimester of pregnancy

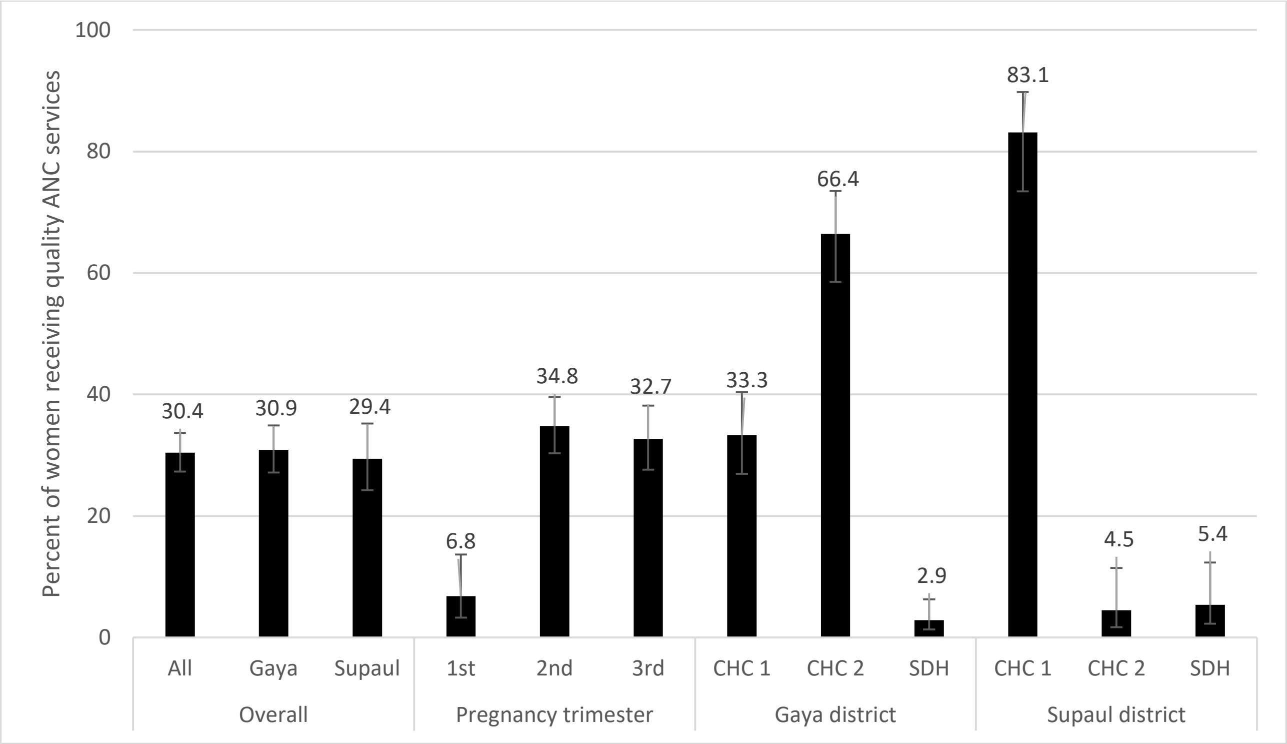

B. Not considering abdomen check-up for women in 1st trimester of pregnancy

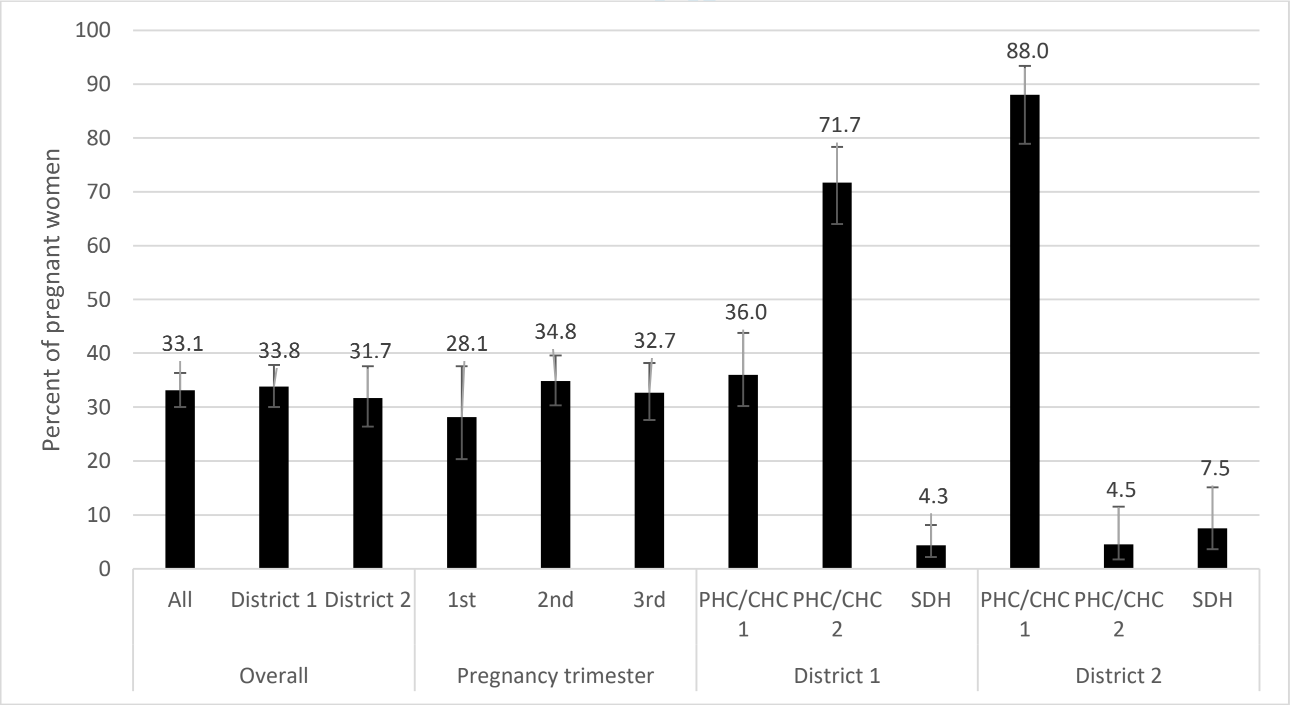

Supplementary Table 1: Select characteristics of the pregnant women who participated in the exit survey.

Background characteristics	All women				Women in Gaya district				Women in Supaul district			
	Total N=814	In 1st trimester N =103 (%)	In 2nd trimester N=405 (%)	In 3rd trimester N=306 (%)	Total N=549	In 1st trimester of pregnancy N=69 (%)	In 2nd trimester of pregnancy N=273 (%)	In 3rd trimester of pregnancy N=207 (%)	Total N=265	In 1st trimester N=34 (%)	In 2nd trimester N=132 (%)	In 3rd trimester N=99 (%)
**Maternal age**												
15-19 years	**118**	13 (12.6)	68 (16.8)	37 (12.1)	**74**	9 (13.0)	43 (15.7)	22 (10.7)	**44**	4 (11.8)	25 (18.9)	15 (15.1)
20-24 years	**488**	69 (67.0)	242 (59.7)	176 (57.7)	**332**	46 (66.7)	159 (58.2)	126 (61.2)	**156**	23 (67.6)	83 (62.9)	50 (50.5)
25-29 years	**174**	18 (17.5)	83 (20.5)	73 (23.9)	**119**	13 (18.8)	62 (22.7)	44 (21.4)	**55**	5 (14.7)	21 (15.9)	29 (29.3)
30-40 years	**34**	3 (2.9)	12 (3.0)	19 (6. 2)	**24**	1 (1.4)	9 (3.3)	14 (6.8)	**10**	2 (5.9)	3 (2.3)	5 (5.1)
**Maternal caste***												
Scheduled caste/tribe	**259**	27 (26.7)	129 (32.3)	103 (34.3)	**201**	25 (36.2)	98 (35.9)	78 (37.7)	**58**	2 (6.3)	31 (24.4)	25 (26.9)
Other backward class	**457**	64 (63.4)	232 (58.0)	161 (53.7)	**311**	39 (56.5)	158(57.9)	114(55.1)	**146**	25 (78.1)	74 (58.3)	47 (50.5)
General	**85**	10 (9.9)	39 (9.7)	36 (12.0)	**37**	5 (7.3)	17 (6.2)	15 (7.3)	**48**	5 (15.6)	22 (17.3)	21 (22.6)
**Number of ANC visit on the day of interview**												
1st visit	**259**	61 (59.2)	134(33.1)	64 (20.9)	**189**	44 (63.7)	101 (37.0)	44 (21.3)	**70**	17 (50.0)	33 (25.0)	20 (20.2)
2nd or 3rd visit	**273**	33 (32.0)	145(35.8)	95 (31.0)	**177**	19 (27.5)	90 (32.9)	68 (32.8)	**96**	14 (41.2)	55 (41.6)	27 (27.3)
4th visit or more	**282**	9 (8.7)	126 31.1)	147 (48.0)	**183**	6 (8.7)	82 (30.0)	95 (45.9)	**99**	3 (8.8)	44 (33.3)	52 (52.5)

**Data not available for 13 pregnant women*

For peer review only

Supplementary Table 2. Coverage of quality of ANC services on the PMSMA day with maternal age and caste of women who participated in the exit survey. CI denotes confidence interval.

Background	All women					Women in Gaya district					Women in Supaul district				
	Total N=814	In 1st trimester with abdomen check-up N (%; (95% CI)	In 1st trimester without abdomen check-up N (%; (95% CI)	In 2nd trimester N (%; (95% CI)	In 3rd trimester N (%; (95% CI)	Total N=549	In 1st trimester with abdomen check-up N (%; (95% CI)	In 1st trimester without abdomen check-up N (%; (95% CI))	In 2nd trimester N (%; (95% CI)	In 3rd trimester N (%; (95% CI)	Total N=265	In 1st trimester with abdomen check-up N (%; (95% CI)	In 1st trimester without abdomen check-up N (%; (95% CI)	In 2nd trimester N (%; (95% CI)	In 3rd trimester N (%; (95% CI)
**Maternal age**															
15-19 years	**118**	15.4 (3.6-47.1)	38.5 (16.1-67.0)	32.3 (22.2-44.4)	40.5 (25.9-57.1)	**74**	0	33.3 (10.1-69.1)	34.9 (22.1-50.3)	50.0 (29.7-70.3)	**44**	50.0 (8.7-91.3)	50.0 (8.7-91.3)	28.0 (13.7-48.9)	26.7 (9.9-54.7)
20-24 years	**488**	4.3 (1.4-12.9)	24.6 (15.7-36.3)	33.5 (27.8-39.7)	29.5 (23.2-36.7)	**332**	2.2 (0.3-14.6)	28.3 (16.9-43.3)	38.4 (31.1-46.2)	28.6 (21.3-37.1)	**156**	8.7 (2.0-30.7)	17.4 (6.3-39.8)	24.1 (16.0-34.6)	32.0 (20.4-46.3)
25-29 years	**174**	11.1 (2.6-36.6)	27.8 (11.6-53.0)	40.9 (30.8-51.9)	31.5 (21.8-43.1)	**119**	15.4 (3.6-47.3)	23.1 (7.1-54.1)	40.3 (28.8-53.0)	25.0 (14.3-40.0)	**55**	0	40.0 (7.7-84.2)	42.8 (23.5-64.7)	41.4 (24.8-60.2)
30-40 years	**34**	0	66.7 (9.3-97.5)	33.3 (12.4-63.7)	47.4 (26.2-69.5)	**24**	0	0	33.3 (10.2-68.6)	28.6 (10.7-57.3)	**10**	0	100.0	33.3 (2.5-90.7)	100.0
**Maternal caste***															
Scheduled caste/tribe	**259**	3.7 (0.5-23.2)	22.2 (10.1-42.1)	34.1 (26.4-42.8)	32.0 (23.7-41.7)	**201**	4.0 (0.5-25.0)	24.0 (10.8-45.0)	35.7 (26.8-45.7)	34.6 (24.8-45.9)	**58**	0	0	29.0 (15.6-47.6)	24.0 (10.9-44.9)

For peer review only

Other backward class	**457**	9.4 (4.2-19.6)	31.3 (21.0-43.8)	33.6 (27.8-40.0)	31.7 (24.9-39.3)	**311**	5.1 (1.2-19.0)	33.3 (20.1-49.8)	38.6 (31.3-46.5)	30.7 (22.9-39.8)	**146**	16.0 (5.8-37.2)	28.0 (13.3-49.6)	23.0 (14.7-34.1)	34.0 (21.8-48.9)
General	**85**	0	20.0 (4.6-56.6)	38.5 (24.5-54.6)	33.3 (19.8-50.3)	**37**	0	0	47.1 (24.9-70.4)	6.7 (0.9-37.1)	**48**	0	40.0 (7.7-84.2)	31.8 (15.6-54.1)	52.4 (31.1-72.8)

**Data not available for 13 pregnant women*

For peer review only

Supplementary Table 3: Availability of the staff on the PMSMA day to provide antenatal care services. NA denotes not applicable.

District, Facility	Number of staff							Total pregnant women registered for ANC services	Average number of pregnant women			
	Doctor	Staff nurse	Lab technician	ANM	Counsellor	Pharmacist	Total		Per staff	Per doctor	Per ANM	Per lab technician
**Gaya, CHC 1**												
Round 1	1	0	1	3	0	1	6	70	12	70	23.3	70
Round 2	2	0	0	4	0	1	7	69	10	34.5	17.3	NA
Round 3	2	0	1	5	0	1	9	74	8	37	14.8	74
**Gaya, CHC 2**												
Round 1	1	0	2	4	0	1	8	65	8	65	16.3	32.5
Round 2	1	0	2	4	0	1	8	41	5	41	10.3	20.5
Round 3	1	0	2	2	0	1	6	79	13	79	39.5	39.5
**Gaya, SDH**												
Round 1	2	2	2	2	1	1	10	89	9	44.5	44.5	44.5
Round 2	2	0	1	2	1	1	7	71	10	35.5	35.5	71
Round 3	4	0	1	2	0	1	8	102	12	25.5	51	102

For peer review only

**Supaul, CHC 1**												
Round 1	3	0	1	12	0	0	16	122	8	40.7	10.2	122
**Supaul, CHC 2**												
Round 1	1	0	1	5	0	0	7	118	16	118	23.6	118
**Supaul, SDH**												
Round 1	2	1	2	5	1	1	12	124	10	62	24.8	62

For peer review only

Supplementary Figure 1. Coverage of each component of antenatal care (ANC) and quality of ANC services among the 306 pregnant women in their 3rd trimester by the number of their ANC visit.

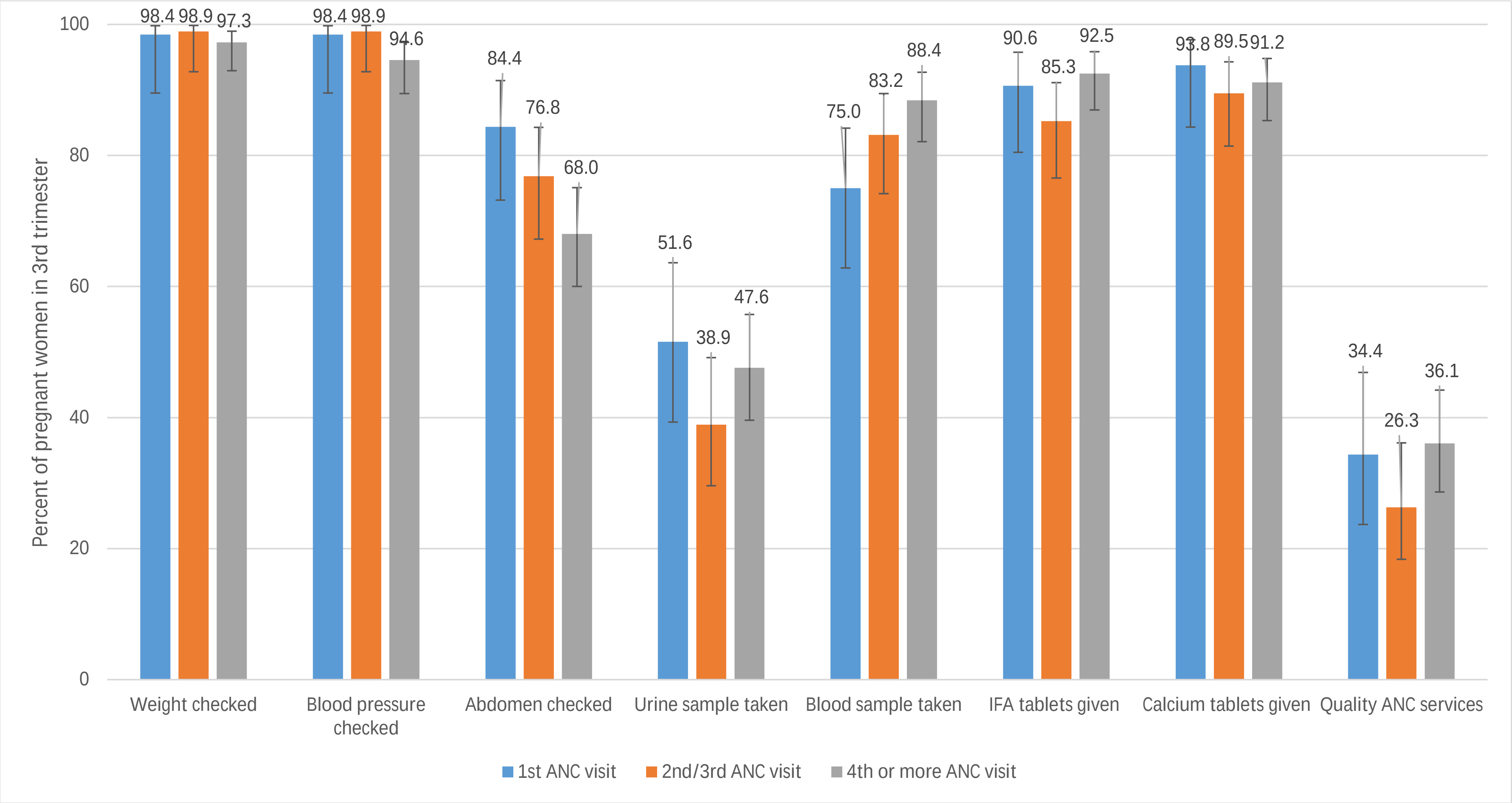

Supplmentary Figure 2: Make-shift toilet facility in one of the public sector health facilities for pregnant women to provide urine sample on PMSMA day.

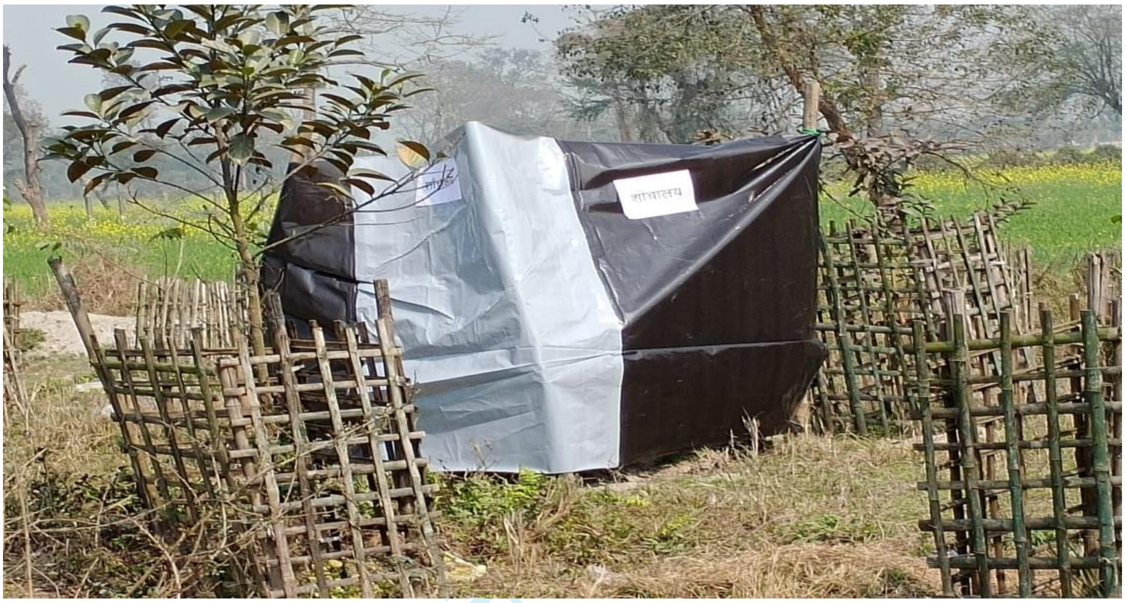

For peer review only - http://bmjopen.bmj.com/site/about/guidelines.xhtml

STROBE Statement—Checklist of items that should be included in reports of ***cross-sectional studies***

	Item No	Recommendation	MANUSCRIPT
**Title and abstract**	1	(*a*) Indicate the study's design with a commonly used term in the title or the abstract	Prospective term used
		(*b*) Provide in the abstract an informative and balanced summary of what was done and what was found	Provided
**Introduction**			
Background/rationale	2	Explain the scientific background and rationale for the investigation being reported	Page 5, lines 74-82
Objectives	3	State specific objectives, including any pre-specified hypotheses	Page 5, lines 83-96
**Methods**			
Study design	4	Present key elements of study design early in the paper	Done
Setting	5	Describe the setting, locations, and relevant dates, including periods of recruitment, exposure, follow-up, and data collection	Page 6, lines 99-110<br>Page 6, lines 117-122
Participants	6	(*a*) Give the eligibility criteria, and the sources and methods of selection of participants	Page 6, lines 110-115
Variables	7	Clearly define all outcomes, exposures, predictors, potential confounders, and effect modifiers. Give diagnostic criteria, if applicable	Pages 7-8
Data sources/ measurement	8*	For each variable of interest, give sources of data and details of methods of assessment (measurement). Describe comparability of assessment methods if there is more than one group	
Bias	9	Describe any efforts to address potential sources of bias	
Study size	10	Explain how the study size was arrived at	
Quantitative variables	11	Explain how quantitative variables were handled in the analyses. If applicable, describe which groupings were chosen and why	
Statistical methods	12	(*a*) Describe all statistical methods, including those used to control for confounding	
		(*b*) Describe any methods used to examine subgroups and interactions	
		(*c*) Explain how missing data were addressed	
		(*d*) If applicable, describe analytical methods taking account of sampling strategy	
		(*e*) Describe any sensitivity analyses	
**Results**			
Participants	13*	(a) Report numbers of individuals at each stage of study—eg numbers potentially eligible, examined for eligibility, confirmed eligible, included in the study, completing follow-up, and analysed	Page 9, lines 167-172
		(b) Give reasons for non-participation at each stage	
		(c) Consider use of a flow diagram	

For peer review only

	Item No	Recommendation	MANUSCRIPT
Descriptive data	14*	(a) Give characteristics of study participants (eg demographic, clinical, social) and information on exposures and potential confounders	
		(b) Indicate number of participants with missing data for each variable of interest	
Outcome data	15*	Report numbers of outcome events or summary measures	Table 1, Figure 1
Main results	16	(*a*) Give unadjusted estimates and, if applicable, confounder-adjusted estimates and their precision (eg, 95% confidence interval). Make clear which confounders were adjusted for and why they were included	Pages 7-9
		(*b*) Report category boundaries when continuous variables were categorized	
		(*c*) If relevant, consider translating estimates of relative risk into absolute risk for a meaningful time period	
Other analyses	17	Report other analyses done—eg analyses of subgroups and interactions, and sensitivity analyses	
**Discussion**			
Key results	18	Summarise key results with reference to study objectives	Page 16, lines 27-280
Limitations	19	Discuss limitations of the study, taking into account sources of potential bias or imprecision. Discuss both direction and magnitude of any potential bias	Page 18, lines 332-343
Interpretation	20	Give a cautious overall interpretation of results considering objectives, limitations, multiplicity of analyses, results from similar studies, and other relevant evidence	Pages 16-19
Generalisability	21	Discuss the generalisability (external validity) of the study results	
**Other information**			
Funding	22	Give the source of funding and the role of the funders for the present study and, if applicable, for the original study on which the present article is based	Page 20

*Give information separately for exposed and unexposed groups.

Note: An Explanation and Elaboration article discusses each checklist item and gives methodological background and published examples of transparent reporting. The STROBE checklist is best used in conjunction with this article (freely available on the Web sites of PLoS Medicine at http://www.plosmedicine.org/, Annals of Internal Medicine at http://www.annals.org/, and Epidemiology at http://www.epidem.com/). Information on the STROBE Initiative is available at www.strobe-statement.org.

For peer review only

For peer review only - http://bmjopen.bmj.com/site/about/guidelines.xhtml

BMJ Open

Prospective assessment of quality of antenatal care services in public-sector facilities in India

Journal:	*BMJ Open*
Manuscript ID	bmjopen-2022-065200.R1
Article Type:	Original research
Date Submitted by the Author:	30-Sep-2022
Complete List of Authors:	Dandona, Rakhi; Public Health Foundation of India; University of Washington<br>Majumder, Moutushi; Public Health Foundation of India<br>Akbar, Md.; Public Health Foundation of India<br>Bhattacharya, Debarshi; Bill & Melinda Gates Foundation India<br>Nanda, Priya; Bill and Melinda Gates Foundation India<br>KUMAR, ANIL; Public Health Foundation of India<br>Dandona, Lalit; Public Health Foundation of India
**Primary Subject Heading**:	Public health
Secondary Subject Heading:	Health services research, Patient-centred medicine
Keywords:	Maternal medicine < OBSTETRICS, Community child health < PAEDIATRICS, PUBLIC HEALTH

SCHOLARONE™
Manuscripts

For peer review only - http://bmjopen.bmj.com/site/about/guidelines.xhtml

I, the Submitting Author has the right to grant and does grant on behalf of all authors of the Work (as defined in the below author licence), an exclusive licence and/or a non-exclusive licence for contributions from authors who are: i) UK Crown employees; ii) where BMJ has agreed a CC-BY licence shall apply, and/or iii) in accordance with the terms applicable for US Federal Government officers or employees acting as part of their official duties; on a worldwide, perpetual, irrevocable, royalty-free basis to BMJ Publishing Group Ltd ("BMJ") its licensees and where the relevant Journal is co-owned by BMJ to the co-owners of the Journal, to publish the Work in this journal and any other BMJ products and to exploit all rights, as set out in our licence.

The Submitting Author accepts and understands that any supply made under these terms is made by BMJ to the Submitting Author unless you are acting as an employee on behalf of your employer or a postgraduate student of an affiliated institution which is paying any applicable article publishing charge ("APC") for Open Access articles. Where the Submitting Author wishes to make the Work available on an Open Access basis (and intends to pay the relevant APC), the terms of reuse of such Open Access shall be governed by a Creative Commons licence – details of these licences and which Creative Commons licence will apply to this Work are set out in our licence referred to above.

Other than as permitted in any relevant BMJ Author's Self Archiving Policies, I confirm this Work has not been accepted for publication elsewhere, is not being considered for publication elsewhere and does not duplicate material already published. I confirm all authors consent to publication of this Work and authorise the granting of this licence.

For peer review only

30 September 2022

Prospective assessment of quality of antenatal care services in public-sector facilities in India

Rakhi Dandona,[1,2] Moutushi Majumder,[1] Md. Akbar,[1] Debarshi Bhattacharya,[3] Priya Nanda,[3] G. Anil Kumar,[1] Lalit Dandona[1,2]

[1]Public Health Foundation of India, Gurugram, National Capital Region, India

[2]Institute for Health Metrics and Evaluation, University of Washington, Seattle, USA

[3]Bill & Melinda Gates Foundation, India Country Office, New Delhi, India

Corresponding author: Prof. Rakhi Dandona, Public Health Foundation of India, Plot 47, Sector 44, Gurugram, Haryana – 122 002, India; rakhi.dandona@phfi.org

Word count: 3,934

For peer review only

For peer review only - http://bmjopen.bmj.com/site/about/guidelines.xhtml

ABSTRACT

Objectives: We undertook a prospective assessment of quality of ANC services in public sector facilities in the Indian state of Bihar state delivered under the national ANC program (PMSMA).

Setting: Three community health centres and one sub-district hospital each in two randomly selected districts of Bihar.

Participants: Pregnant women who sought ANC services under PMSMA irrespective of the pregnancy trimester.

Primary and secondary measures: Quality ANC services was considered if a woman received all of these services in that visit - weight, blood pressure and abdomen check, urine and blood sample taken, and were given iron and folic acid and calcium tablets. The process of ANC service provision was documented.

Results: Eight hundred fourteen (94.5% participation) women participated. Coverage of quality ANC services was 30.4% (95% CI 27.3-33.7) irrespective of pregnancy trimester, and was similar in both districts and ranged 3% to 83.1% across the facilities. Quality ANC service coverage was significantly lower for women in the 1st trimester of pregnancy (6.8%, 95% CI 3.3-13.6) as compared with those in the 2nd (34.4%, 95% CI 29.9-39.1) and 3rd (32.9%, 95% CI 27.9-38.3) trimester of pregnancy. Individually, the coverage of weight and blood pressure check-up, receipt of IFA and calcium tablets, and blood sample collection was >85%. The coverage of urine sample collection was 46.3% (95% CI 42.9-49.7) and of abdomen check-up was 62% (95% CI 58.6-65.3). Poor information sharing post check-up was done with the pregnant women. Varied implementation of ANC service provision was seen in the facilities as compared with the PMSMA guidelines, in particular with laboratory diagnostics and doctor consultation. Task shifting from doctors to ANMs was observed in all facilities.

Conclusions: Grossly inadequate quality ANC services under the PMSMA needs urgent attention to improve maternal and neonatal health outcomes.

For peer review only

For peer review only - http://bmjopen.bmj.com/site/about/guidelines.xhtml

Keywords: Antenatal care, coverage, pregnancy, quality, Bihar, India

STRENGTHS AND LIMITATIONS OF THE STUDY

- Data on the quality of antenatal care services received by pregnant women documented prospectively in public sector health facilities using exit survey
- Information provided to the pregnant women after the antenatal care check-up documented in exit survey
- Direct observations made for the process of antenatal care services offered in the public sector health facilities

For peer review only

For peer review only - http://bmjopen.bmj.com/site/about/guidelines.xhtml

BACKGOUND

The inadequacy of and inequity in quality of antenatal care (ANC) services is increasingly receiving attention.[1-7] The key elements of ANC services captured under the facility surveys also indicate inadequate quality of care provision.[8] Concerns about the quality of ANC services have also been reported from India, primarily based on the retrospective data collected under the National Family Health Survey.[1 9-11] The ANC quality in these reports is captured retrospectively and does not offer nuanced understanding of what happens in a particular ANC visit with a health provider , thereby, limiting the actions needed to address the coverage-quality gap in ANC services.

To increase the coverage and quality of ANC services, the Government of India started the *Pradhan Mantri Surakshit Matritva Abhiyan* (PMSMA; Prime Minister's Safe Motherhood Program).[12] PMSMA is based on the premise that if every pregnant woman in India is examined by a physician and appropriately investigated at least once during the PMSMA and then appropriately followed up, the process can result in reduction in the number of maternal and neonatal deaths in the country. With over 18,000 facilities providing PMSMA services, nearly 28.1 million pregnant women have been examined in India under this program since its inception in 2016.[12] However, no comprehensive assessment of ANC services under the PMSMA is available in the public domain. In this context , we undertook a prospective health facility based assessment of ANC services offered on the PMSMA day in the Indian state of Bihar, one of the most populous Indian states.[13] The coverage of at least one ANC visit in the state is at 82% and that for 4 ANC visits at 25.2%,[14] with the neonatal mortality rate in 2017 at 23.4 per 1,000 livebirths and maternal mortality ratio of 230.[15 16] We report on the quality of ANC services, information sharing with the pregnant women based on the ANC check-up, and deviations from the prescribed process of ANC service provision under PMSMA with the aim to identify the areas that needed attention for the PMSMA to achieve its stated goal.

For peer review only - http://bmjopen.bmj.com/site/about/guidelines.xhtml

METHODS

Study design

A facility based prospective study in two districts of Bihar state was undertaken which was approved by the Institutional review board of Public Health Foundation of India. All participants provided written informed consent, and for those who could not read or write, the participant information sheet and consent form were explained by the trained interviewer and a thumb impression obtained.

Selection of health facilities

A multi-stage sampling process was followed to select the public sector health facilities for this study. First, two districts from the state were sampled based on the latest available 3+ visits ANC coverage for Bihar for 2016 at the time of planning of this study.[17] All the 38 districts of the state were grouped into two strata as same/above and below the state median coverage for 3+ visits ANC visits (29.5%). We then randomly selected one district each from these two groups for this study – Gaya and Supaul. In each of the two sampled districts, three block-level community health centres (CHC) and one sub-district hospital (SDH) were purposively sampled from the public sector health facilities where detailed assessments were already being undertaken.

Selection of pregnant women

All pregnant women who reported for ANC services to the sampled health facilities, irrespective of the trimester of pregnancy, on the 9th day of the study month under the PMSMA scheme were considered. The only exclusion criterion was non-availability of a phone number for contact later for follow-up assessment. We aimed to recruit 200 pregnant women in each of the sampled facilities giving a total sample of 1,200 women across the six facilities.

Data collection

Data collection began in September 2019 in Gaya district but was then withheld in October and November 2019 due to floods and socio-political issues in the state, and was next two rounds were completed in December 2019 and January 2020. Data collection in Supaul district was started in

February 2020 and could not be continued due to the Covid-19 pandemic lockdown. Hence, the data available for analysis was for three rounds in Gaya district and for one round of data collection in Supaul district. The permission to conduct the study was sought from the health facility-in-charge.

Exit interviews

In each data collection round, exit interviews were conducted with the pregnant women. Interviewers trained in the study procedures contacted pregnant women after they registered at the out-patient department (OPD) registration for ANC services. Those who were able to provide a phone number were explained in brief about the purpose of the study and requested to wait after they had availed ANC services on that day. The team posted at the health facility kept track of these women through the various steps of ANC services, and re-contacted them after they had availed the services. They were provided refreshments and explained the purpose of the study in detail. Those who consented were interviewed in a semi-private place in the facility premises. The exit interviews documented socio-demographic information of the pregnant woman, details of the ANC services provided on the given PMSMA day, women’s knowledge of the services received and what they were informed post the check-up.

The exit interview tool was developed in English and then translated into Hindi (local language), after which back translated into English to ensure accurate and relevant meaning and intent of the questions. Pilot testing of the tool was carried out and modifications made as necessary. The interviews were captured using the CAPI software in handheld tablets. Data entered was scrutinised using the internal checks, and some portion of the 30% of all interviews were also attended by a supervisor for quality control purpose with prior permission taken from the respondent to do so.

Observation of the flow of ANC services

The interviewers placed in each facility for the exit interviews also observed the step-wise flow of ANC service provision followed for the pregnant women. The entire process followed on a given day was documented in a format specifically developed to track the process from the beginning till the

For peer review only

end. The number and type of staff providing ANC services and the number of pregnant women registered for ANC services were documented.

Data analysis

We present the coverage of each ANC component by the pregnancy trimester, facility and district from the exit interviews. Whether pregnant women have received some or all components of a set of interventions as part of ANC at least once during pregnancy has been used to indicate quality of care.[18-20] For this analysis, we defined quality ANC service when a pregnant woman reported receiving all of the following ANC service components in the exit interview - weight measurement, blood pressure check-up, abdomen check-up, urine sample taken, blood sample taken, iron and folic acid and calcium tablets given - on that PMSMA day. We report coverage of quality ANC services by select socio-demographic and pregnancy trimester, district and type of health facility. We did not include tetanus toxoid in this assessment as it's administration in pregnant women is dependent on certain factors.[21] The tetanus toxoid coverage for last pregnancy in Bihar was reported at 89.5% in the most recent state-wide assessment.[14] Though the PMSMA is primarily designed for women in 2nd and 3rd trimester of pregnancy, in reality, women in the 1st trimester also avail ANC services on PMSMA day. Therefore, we include these women in our analysis and present findings separately by pregnancy trimester; and report coverage of quality ANC services for the women in 1st trimester of pregnancy with and without considering abdomen check-up. We also report on whether the pregnant women were informed about the check-up provided to them and of the clinical findings by the health providers.

Using the facility observations of the ANC services flow, we compared the steps followed in the ANC service provision in each facility against what is recommended in the PMSMA guidelines to highlight the issues in process that could have implications on the quality of service provision.[22] STATA 13.1 version was used for all analysis.

For peer review only - http://bmjopen.bmj.com/site/about/guidelines.xhtml

156 **Patient and Public Involvement statement:** Patients were not involved in planning of this research
157 study.

For peer review only

For peer review only - http://bmjopen.bmj.com/site/about/guidelines.xhtml

RESULTS

A total of 961 pregnant women were identified for exit interviews of whom 861 (89.6%) were eligible for the study, and of whom 814 (94.5%) participated. Supplementary Table 1 in Supplementary file documents the distribution of pregnant women based on the pregnancy trimester. Many of the pregnant women were in 20-24 years' age group (59.5%), 711 (87.3%) women were in their 2nd or 3rd trimester of pregnancy, and women belonging to other than forward caste (88%) accounted for most of the sample across the facilities (Supplementary Table 1 in Supplementary file).

Coverage of each ANC component

The component-wise coverage of ANC services as reported in the exit interviews by the pregnant women is shown in Supplementary Figure 1 and Supplementary Table 2 in Supplementary file. Overall, weight check-up was reported by 98.4% (95% CI 97.3-99.1) and blood pressure check-up by 97.1% (95% CI 95.7-98.2) of the pregnant women (Supplementary Figure 1 in Supplementary file). There was no significant difference in provision of these two components either by the pregnancy trimester or by the health facility (Supplementary Figure 1 in Supplementary file). Though 80% of the women for whom weight was checked were also informed of the weight reading, less than one-third of them were informed about appropriateness of the weight gain as per the pregnancy trimester (Table 1). Of the 790 women for whom blood pressure was checked, only 463 (58.7%) were informed the blood pressure reading and of them only 223 (28.2%) were informed of the appropriateness of the reading (Table 1). There was considerable variation in women being informed about blood pressure by the pregnancy trimester (Table 1).

Table 1: Distribution of pregnant women who participated in the exit interviews based on the component-wise antenatal care service provided on the given day and information provided to pregnant women regarding each of those components. CI denotes confidence interval and NA denotes not applicable.

Service provided and women informed	All women N =814 (%)	Women in 1st trimester of pregnancy N=103 % (95% CI)	Women in 2nd trimester of pregnancy N=405 % (95% CI)	Women in 3rd trimester of pregnancy N=306 % (95% CI)

For peer review only - http://bmjopen.bmj.com/site/about/guidelines.xhtml

Weight checked	801 (98.4)	99<br>96.1 (89.9-98.5)	402<br>99.3 (97.7-99.7)	300<br>98.0 (95.7-99.1)
Informed the weight reading for whom weight was checked	708 (88.4)	82<br>82. 8 (74.0-89.1)	363<br>90.3 (86.9-92.8)	263<br>87.7 (83.4-90.9)
Informed about appropriateness of weight gain by the stage of pregnancy for whom weight was checked	246 (30.7)	32<br>32.3 (23.8-42.2)	125<br>31.1 (26.7-35.8)	89<br>29.7 (24.7-35.1)
Blood pressure checked	790 (97.0)	99<br>96.1 (90.1-98.5)	395<br>97.5 (95.5-98.7)	296<br>96.7 (94.0-98.2)
Informed the blood pressure reading for whom blood pressure was checked	463 (58.7)	56<br>56.6 (46.6-66.0)	237<br>60.1 (55.2-64.9)	170<br>57.6 (51.9-63.2)
Informed about the appropriateness of blood pressure reading for whom blood pressure was checked	223 (28.2)	28<br>28.3 (20.2-38.0)	114<br>28.9 (24.6-33.5)	81<br>27.4 (22.6-32.7)
Blood sample taken	697 (85.6)	94<br>91.3 (83.9-95.4)	346<br>85.4 (81.6-88.5)	257<br>83.9 (79.4-87.7)
Informed that anaemia test will be done for whom blood sample was taken	87 (12.5)	13<br>13.8 (8.2-22.4)	43<br>12.4 (9.3-16.3)	31<br>12.1 (8.6-16.7)
Informed about the haemoglobin level for whom blood sample was taken	235 (33.7)	41<br>43.6 (33.9-53.8)	123<br>35.5 (30.7-40.7)	71<br>27.6 (22.5-33.4)
Informed about blood sugar level for whom blood sample was taken	19 (2.7)	1<br>1.1 (0.1-7.3)	12<br>3. 5 (2.0-6.0)	6<br>2.3 (1.0-5.1)
Abdomen checked	505 (62.0)	25<br>24.3 (16.9-33.5)	253<br>62.5 (57.6-67.1)	227<br>74.2 (69.0-78.8)
Explained the process to monitor baby movements for whom abdomen was checked	33 (7.7)	NA	19<br>9.4 (6.0-14.2)	15<br>6.6 (4.0-10.7)
IFA tablets given	736 (90.4)	86<br>83.5 (74.9-89.5)	375<br>92.6 (89.6-94.8)	275<br>89.8 (85.9-92.8)
Informed about the benefits and side-effects to whom IFA tablets were given	193 (26.2)	19<br>23.5 (15.4-34.0)	91<br>26.2 (21.8-31.1)	83<br>31.6 (26.2-37.4)
Calcium tablets given	750 (92.1)	88<br>85.4 (77.2-91.1)	383<br>94.6 (91.9-96.4)	279<br>91.2 (87.8-94.1)
Informed about benefits to whom calcium tablets were given	114 (15.6)	8<br>9.3 (4.7-17.6)	61<br>16.4 (13.0-20.6)	45<br>16.6 (12.6-21.5)

For peer review only - http://bmjopen.bmj.com/site/about/guidelines.xhtml

The urine sample collection coverage was 46.3% (95% CI 42.9-49.7) with significant variations seen by district, pregnancy trimester and facility (Supplementary Figure 1 in Supplementary file). The blood sample collection coverage was 85.6% (95% CI 83.0-87.8), with no significant variation by pregnancy trimester (Supplementary Figure 1 in Supplementary file). Of the 697 women for whom blood sample was taken, only 235 (33.7%) were informed about the haemoglobin value, and almost none were informed about the blood sugar value (Table 1).

The coverage of abdomen check-up was 62% (95% CI 58.6-65.3) and significant variations were seen by the pregnancy trimester and health facility (Supplementary Figure 1 in Supplementary file). Only 7.7% women of the women in 2nd or 3rd trimester were explained how to monitor baby's movements (Table 1). Overall 90.4% (95% CI 88.2-92.2) of the pregnant women reported receiving IFA tablets and 92.1% (95% CI 90.1-93.8) receiving calcium tablets, with no significant variations by pregnancy trimester or health facility (Supplementary Figure 1 in Supplementary file). Of the 736 women who had received IFA tablets, only 193 (26.2%) were informed about the benefits and side effects of it. Only 114 (15.6%) women of the 750 women who had received calcium tablets were informed about the benefits of it (Table 1).

Quality ANC services

The coverage of quality ANC services irrespective of the pregnancy trimester was 30.4% (95% CI 27.3-33.7), and was similar in both the districts (Figure 1A) and by maternal age and caste (Supplementary table 2 in Supplementary file). This coverage was significantly lower for women in the 1st trimester of pregnancy (6.8%, 95% CI 3.3-13.6) as compared with those in the 2nd or 3rd trimester of pregnancy (Figure 1A). This coverage varied significantly between the facilities, and ranged from 3-66% in district 1 and 4.5-83.1% in district 2 (Figure 1A).

On not considering the abdomen check-up for women in the 1st trimester of pregnancy (Figure 1B), quality ANC service coverage increased for them to 28.1% (95% CI 20.3- 37.6), and the pattern distribution by maternal age and caste was similar to that with inclusion of abdomen check-up (Supplementary table 2 in Supplementary file).

For peer review only - http://bmjopen.bmj.com/site/about/guidelines.xhtml

Among the 306 pregnant women in their 3rd trimester of pregnancy (Supplementary Table 1 in Supplementary file), 64 (20.9%) women had come for their 1st ANC visit, 95 (31.0%) for their 2nd or 3rd ANC visit, and 147 (48%) for their 4th ANC visit or more. Not much variation was seen in the coverage of individual ANC components based on the number of ANC visit that they were in for (Supplementary Figure 2 in Supplementary file). The coverage of quality ANC services was 34.4%, 26.3%, and 36.1% for women who had come for their 1st, 2nd or 3rd, 4th visit or more ANC visit (Supplementary Figure 2 in Supplementary file).

Process of ANC services

The number and cadre of staff available for ANC services on the PMSMA day ranged from 6 to 13 across the various rounds of data collection in the two districts (Supplementary Table 3 in Supplementary file). The average number of pregnant women examined per doctor (range 25.5 to 118), ANM (range 10.3 to 44.5) and lab technician (range 20.5 to 122) varied significantly between and within the facilities. The proportion of coverage of quality ANC services varied from 1.5% to 90.3%; a decrease in the proportion of quality ANC services was seen with increasing number of pregnant women seen per staff (p=0.230) and per ANM (p=0.121) but this was not statistically significant.

The process as per the PMSMA guidelines and what was observed in the facilities is shown in Table 2. In all the facilities, pregnant women registered for ANC check-up at the registration counter and were given “Out-patient department (OPD) slip” with a number. This OPD number was relevant only for that day and no facility had a system of tracking if a woman had visited for ANC services earlier. As per the PMSMA guidelines, step 2 is medical check-up done by ANMs followed by laboratory investigations (step 3), collection of laboratory reports (step 4), and with this the pregnant women are seen by the doctor (step 5). However, such a process was not observed in any facility (Table 2).

For peer review only - http://bmjopen.bmj.com/site/about/guidelines.xhtml

Table 2: Process of antenatal care services as observed in the study facilities. CHC denotes Community Health Centre and SDH denotes Sub-district Hospital.

Steps suggested in the PMSMA guidelines		Steps followed at the sampled facilities					
**Step**	**Detail**	**Gaya district CHC 1**	**Gaya district CHC 2**	**Gaya district SDH**	**Supaul district CHC 1**	**Supaul district CHC 2**	**Supaul district SDH**
1	Registration	Registration	Registration	Registration	Registration	Registration	Registration
2	ANC OPD by ANM/Nurse	Doctor's consultation	Doctor's consultation	Doctor's consultation	Receipt of medicines	Laboratory investigations	ANC OPD by ANM/Nurse
3	Laboratory investigations	Laboratory investigations	Laboratory investigations	Laboratory investigations	Laboratory investigations	Collection of laboratory reports	Doctor's consultation
4	Collection of laboratory reports	Collection of laboratory reports	Collection of laboratory reports	Collection of laboratory reports	Collection of laboratory reports	ANC OPD by ANM/Nurse	Laboratory investigations
5	Doctor's consultation	ANC OPD by ANM/Nurse	ANC OPD by ANM/Nurse	ANC OPD by ANM/Nurse	Doctor's consultation	Doctor's consultation	Collection of laboratory reports
6	Counselling	Receipt of medicines	Receipt of medicines	Receipt of medicines	ANC OPD by ANM/Nurse	Receipt of medicines	Counselling
7	Receipt of medicines				Counselling		Receipt of medicines
8	Feedback and grievance redressal				Feedback and grievance redressal		Feedback and grievance redressal

Doctor consultation (step 5) was done before the steps 2-4 in 4 of the 6 facilities. Of the 22 doctors who provided ANC services in these facilities across all the rounds, 14 (63.6%) were males; 11 (50%) were MBBS, 2 (9.1 %) were MD, 4 (18.2 %) were dental specialist, 5 (22.7%) were alternate medicine doctors. A total of 349 (65.9%) and 151 (58.9%) women in Gaya and Supaul districts were seen by a doctor, respectively. The doctors were observed mainly handing over prescription indicating blood and urine tests to be done, and the list of medicines to be provided to the pregnant women in all facilities except one where the doctor performed medical check-up.

Urine sample was taken only for 276 (50.3%) and 101 (38.1%) women in Gaya and Supaul districts, respectively. In some facilities, women were given new containers for urine sample collection and in some old containers, and all tests were done only using dipstick. No facility had storage option available for urine samples. The laboratory technician noted name and serial number for each pregnant woman in the laboratory register, and indicated the same number on the container. Women were asked to go to the toilet with the container and dipstick, to dip the dipstick one inch in the urine, and bring it back to the technician. No laboratory technician was seen handling the dipstick; women were asked to hold it up and the technician recorded the reading from a distance. The field team noticed make-shift toilet (Supplementary Figure 3 in Supplementary file) in one facility and the toilet in one facility was on another floor than the ANC OPD. Pregnant women were seen hesitating in using both of these toilets, and some did not take the urine test even after being given the container. In the remaining facilities, pregnant women had to use a toilet elsewhere which was not close to the laboratory. Some were noticed being embarrassed at carrying the container and dipstick as the other people present in the facility could see them. Blood sample was taken only for 437 (79.6%) and 260 (98.1%) women in Gaya and Supaul districts, respectively. Venous blood was drawn for 250 (35.9%) and the rest was finger prick method. The laboratory technicians were not seen wearing gloves every time they took a blood sample. Blood samples were discarded immediately after recording of the haemoglobin value in all facilities.

For peer review only - http://bmjopen.bmj.com/site/about/guidelines.xhtml

Almost all the check-up that the pregnant women were offered was done by the ANMs in all the facilities. None of the clinical findings by the ANMs were checked by the doctor as the women were made to see the doctor before any check-up was done. In all facilities, the ANMs entered the clinical data in the PMSMA register next day and not immediately after examining the women. The counselling services (step 6) and feedback redressal (step 7) were seen only in 2 of the 6 facilities. Only 38 (18.3%) and 11 (11.8%) of the women in SDH of Gaya and Supaul districts reported meeting a counsellor, respectively.

For peer review only

For peer review only - http://bmjopen.bmj.com/site/about/guidelines.xhtml

DISCUSSION

In our understanding, this is the first prospective study undertaken in health facility in a developing country setting that provides a systematic documentation of quality of ANC services considering all the ANC components through exit survey, along with documentation of the process of ANC service provision. Only one-third of the pregnant women had received quality ANC services under the PMSMA, and even less were informed about the services that they had received. The wide variations in the implementation of PMSMA at the facilities, and the task shifting for service provision will have to be addressed urgently to improve the quality of ANC service provision.

Two components that resulted most in poor quality of ANC services were urine examination and abdomen check-up. This study has highlighted that good clinical laboratory practice guidelines were not followed for urine examination in any of the facilities.[23] Notably, though the coverage of blood sample examination was relatively higher than that for urine, however, only blood sugar and anaemia levels were recorded and none of the other tests recommended in pregnancy were performed. These findings suggest improving the laboratory readiness is imperative to improve quality of ANC services.[24] The laboratory infrastructure and related processes, and the laboratory technicians as human resources for health have not received the necessary attention in provision of health services until recently in India.[25-27] Laboratory services have recently received unprecedented attention in India due to Covid-19, and it would be important to sustain this and expand it to routine pregnancy laboratory tests as well.[28 29]

The Indian ANC guidelines recommend abdomen check-up from 2nd pregnancy trimester onwards to monitor pregnancy progression, foetal well-being and its position.[24] We have previously reported from this population that breech position is a significant risk factor for early neonatal deaths and stillbirths, however, breech position of the baby is not known to most women before delivery as abdomen check-up is either not done or they are not informed.[30 31] Abnormal foetal movements can be used to predict adverse neonatal outcomes,[32-34] however, only 1 in 12

For peer review only - http://bmjopen.bmj.com/site/about/guidelines.xhtml

women who had received abdomen check-up was informed about monitoring of baby movements in this study.

Weight and blood pressure measurements, and provision of IFA and calcium tablets showed consistently high coverage across all the facilities irrespective of the pregnancy trimester. However, less than one-third of the pregnant women were informed about the findings or implications of these for pregnancy. This coupled with almost non-existence of counselling is a major concern. Low birthweight is reported to be the largest contributor to child malnutrition disability-adjusted-life years in India,[35] and birthweight is an intergenerational issue dependent on an interplay of various factors, including maternal undernutrition and intrauterine growth. Also, anaemia increases the risk of adverse birth outcomes, and the high burden of anaemia in Indian women has not declined since 1990.[35] With very poor communication with the women and almost no counselling, pregnant women are not empowered to take correct steps for a positive pregnancy experience and to prevent adverse birth outcomes.[36]

Substantial inequities in coverage of quality ANC services by socio-economic status have been highlighted in some previous assessments.[1 37] As this study was carried out only in the public sector facilities, we are unable to comment on inequities in general. However, it is important to note that the coverage of quality ANC services in our study was similar irrespective of the caste of women, which is a surrogate measure of socio-economic status in rural India. The new World Health Organization ANC model recommends that women attend a minimum of eight ANC visits.[36] However, with the significantly poor levels of quality ANC services as documented in this study and other assessments, focussing only on increasing the number of visits is unlikely to produce the desired maternal and neonatal health outcomes.[38] With coverage of quality ANC services at 30% on PMSMA day, the effort to improve quality of ANC can go only thus far unless the quality of ANC is explicitly tracked and monitored through standard indicators both at the health system and community levels.[38-40] Thought the India Newborn Action Plan recommends monitoring of

For peer review only

For peer review only - http://bmjopen.bmj.com/site/about/guidelines.xhtml

percentage of pregnant women who received full ANC but the definition of full ANC is not provided.[41]

The varied availability of staff on PMSMA day between and within the facilities over the various rounds of data collection in this study, and the substantial variations found in the steps of PMSMA implementation at the facilities are of concern with regards to the provision of quality services. Despite the premise of PMSMA day being that every pregnant woman in India is examined by a physician and appropriately investigated at least once during the PMSMA,[12] almost all the check-up in our study was provided by ANMs in all the facilities. Notably, the doctor's consultation did not involve any examination of the pregnant women in all but one facility even though the availability of doctors was not an issue in any facility. These findings indicate task shifting which is neither in line with the PMSMA guidelines[12] nor with the task shifting recommendations to maintain the quality of services.[42] Importantly, the program implementation needs to address and account for such task shifting to address the poor quality of ANC services. The wide variation in coverage of quality also indicates the scope for improvement. Raising the performance of all health facilities to the level of best performance should be feasible with more in-depth understanding of implementation issues at the facility level, which would lead to significant overall gains in quality and ensure that pregnant women receive quality services irrespective of the facility they access for these services.[7]

There are some limitations of the study. It was conducted in six facilities only, which could be considered as a limitation to generalise these findings. It is important to note that concerns have also been raised with the quality of maternal services in the private sector as well, and it would be important to assess quality of ANC services for these facilities.[43-46] Furthermore, we were able to conduct data collection for only one round instead of three in one district. However, it is important to note that the study findings corroborate with the previous findings of poor coverage of quality ANC services in household surveys in the state. We excluded 10% of women because of non-

For peer review only

availability of a phone for contacting her later for follow-up assessment. However, this proportion is small to have any significant impact on the study findings.

There are several strengths of this study. There is almost no recall bias in the information reported by the respondents on the ANC services received as this was an exit survey. The prospective nature of this study, documentation of ANC services components beyond what is previously reported, documentation of what women are informed, and the process of ANC services are strengths of this undertaking.

For peer review only - http://bmjopen.bmj.com/site/about/guidelines.xhtml

CONCLUSION

This study highlights grossly inadequate quality of ANC services in public sector health facilities. The findings suggest that in order to provide ANC services as envisaged under the PMSMA, there is an urgent need to cultivate quality in ANC service provision at the public health facilities; to train doctors, ANMs and laboratory technicians to communicate with the pregnant women for a positive pregnancy experience and to address complications; and to build clinical and technical capacity and supervision for health providers to follow the standard guidelines for provision of quality ANC services.

For peer review only

For peer review only - http://bmjopen.bmj.com/site/about/guidelines.xhtml

Ethics approval: This study was approved by the Ethics Committee of the Public Health Foundation of India (TRC-IEC 410.1/19).

Authors' contributions: RD and GAK had full access to data in the study, take full responsibility for the integrity of data and accuracy of the data analysis, and had final responsibility for the decision to submit for publication; RD, GAK and LD conceptualized the study; RD guided the data analysis and drafted the manuscript; MM and GAK performed data analysis; MA guided data collection; MA, DB, and PN contributed to data analysis and interpretation; LD contributed to drafting of the manuscript and interpretation; all authors approved the final manuscript.

Funding: Bill and Melinda Gates Foundation; grant number INV-007989.

Declaration of interests: DB and PN are employees of the Bill & Melinda Gates Foundation, India Country Office, New Delhi. The other authors have no conflict of interest.

Data availability statement sharing: All data generated or analysed during this study are included in this published article and its supplementary information files.

Acknowledgements: The authors acknowledge the contributions of Sibin George and S Siva Prasad from Public Health Foundation of India, and Asif Iqbal and Vipul Singhal from the Oxford Policy Management, India for data collection and data management.

For peer review

REFERENCES

1. Arsenault C, Jordan K, Lee D, et al. Equity in antenatal care quality: an analysis of 91 national household surveys. Lancet Glob Health 2018;**6**(11):e1186-e95 doi: 10.1016/s2214-109x(18)30389-9[published Online First: Epub Date]|.
2. Chukwuma A, Wosu AC, Mbachu C, et al. Quality of antenatal care predicts retention in skilled birth attendance: a multilevel analysis of 28 African countries. BMC Pregnancy Childbirth 2017;**17**(1):152 doi: 10.1186/s12884-017-1337-1[published Online First: Epub Date]|.
3. Yeoh PL, Hornetz K, Shauki NIA, et al. Evaluating the quality of antenatal care and pregnancy outcomes using content and utilization assessment. Int J Qual Health Care 2018;**30**(6):466-71 doi: 10.1093/intqhc/mzy041[published Online First: Epub Date]|.
4. Sommer Albert J, Younas A, Victor G. Quality of Antenatal Care Services in a Developing Country: A Cross-Sectional Survey. Creat Nurs 2020;**26**(1):e25-e34 doi: 10.1891/1078-4535.26.1.e25[published Online First: Epub Date]|.
5. Wynne SJ, Duarte R, de Wildt G, et al. The timing and quality of antenatal care received by women attending a primary care centre in Iquitos, Peru: A facility exit survey. PLoS One 2020;**15**(3):e0229852 doi: 10.1371/journal.pone.0229852[published Online First: Epub Date]|.
6. Gwako GN, Were F, Obimbo MM, et al. Association between utilization and quality of antenatal care with stillbirths in four tertiary hospitals in a low-income urban setting. Acta Obstet Gynecol Scand 2021;**100**(4):676-83 doi: 10.1111/aogs.13956[published Online First: Epub Date]|.
7. Phommachanh S, Essink DR, Jansen M, et al. Improvement of Quality of Antenatal Care (ANC) Service Provision at the Public Health Facilities in Lao PDR: Perspective and Experiences of Supply and Demand Sides. BMC Pregnancy Childbirth 2019;**19**(1):255 doi: 10.1186/s12884-019-2345-0[published Online First: Epub Date]|.
8. Macarayan EK, Gage AD, Doubova SV, et al. Assessment of quality of primary care with facility surveys: a descriptive analysis in ten low-income and middle-income countries. Lancet Glob Health 2018;**6**(11):e1176-e85 doi: 10.1016/s2214-109x(18)30440-6[published Online First: Epub Date]|.
9. Singh L, Dubey R, Singh S, et al. Measuring quality of antenatal care: a secondary analysis of national survey data from India. BJOG 2019;**126 Suppl 4**:7-13 doi: 10.1111/1471-0528.15825[published Online First: Epub Date]|.
10. Munuswamy S, Nakamura K, Seino K, et al. Inequalities in Use of Antenatal Care and Its ServiceComponents in India. J Rural Med 2014;**9**(1):10-9 doi: 10.2185/jrm.2877[published Online First: Epub Date]|.
11. Bucher S, Marete I, Tenge C, et al. A prospective observational description of frequency and timing of antenatal care attendance and coverage of selected interventions from sites in Argentina, Guatemala, India, Kenya, Pakistan and Zambia. Reprod Health 2015;**12 Suppl 2**(Suppl 2):S12 doi: 10.1186/1742-4755-12-s2-s12[published Online First: Epub Date]|.
12. Ministry of Health and Family Welfare GoI. Pradhan Mantri Surakshit Matritva Abhiyan. Secondary Pradhan Mantri Surakshit Matritva Abhiyan 15 March 2021. https://pmsma.nhp.gov.in/about-scheme/#about.
13. Registrar General of India. C.D. Blockwise Primary Census Abstract Data (PCA) - Bihar. Secondary C.D. Blockwise Primary Census Abstract Data (PCA) - Bihar 2011. http://censusindia.gov.in/pca/cdb_pca_census/Houselisting-housing-BR.html.
14. International Institute for Population Sciences. National Family Healty Survey (NFHS-5): Bihar. Mumbai: IIPS, 2020.
15. India State-Level Disease Burden Initiative Child Mortality Collaborators. Subnational mapping of under-5 and neonatal mortality trends in India: the Global Burden of Disease Study 2000-17. Lancet 2020;**395**(10237):1640-58 doi: 10.1016/s0140-6736(20)30471-2[published Online First: Epub Date]|.

For peer review only - http://bmjopen.bmj.com/site/about/guidelines.xhtml

16. Institute for Health Metrics and Evaluation (IHME). Maternal Health Atlas. Secondary Maternal Health Atlas 2018. https://maternalhealthatlas.org/.
17. International Institute for Population Sciences. National Family Healty Survey (NFHS-4) India 2015-16: Bihar. Secondary National Family Healty Survey (NFHS-4) India 2015-16: Bihar 2017. http://rchiips.org/nfhs/NFHS-4Reports/Bihar.pdf.
18. Abou Zahr CWTMWHOU. *Antenatal care in developing countries : promises, achievements, and missed opportunities : an analysis of trends, levels, and differentials, 1990-2001*. Geneva; New York: World Health Organization ; UNICEF, 2003.
19. Carvajal-Aguirre L, Amouzou A, Mehra V, et al. Gap between contact and content in maternal and newborn care: An analysis of data from 20 countries in sub-Saharan Africa. Journal of global health 2017;**7**(2):020501 doi: 10.7189/jogh.07.020501[published Online First: Epub Date]|.
20. Kyei NNA, Chansa C, Gabrysch S. Quality of antenatal care in Zambia: a national assessment. BMC Pregnancy Childbirth 2012;**12**:151-51 doi: 10.1186/1471-2393-12-151[published Online First: Epub Date]|.
21. World Health Organization. Standards for Maternal and Neonatal Care : Maternal immunization against tetanus. In: Safe DoMP, ed. Geneva: WHO.
22. Ministry of Health and Family Welfare GoI, . Pradhan Mantri Surakshit Matritva Abhiyan New Delhi, 2016.
23. Indian Council of Medical Research. ICMR guidelines for good clinical laboratory practices (GCLP) 2021. New Delhi: ICMR, 2021.
24. Ministry of Health and Family Welfare GoI, . Guidelines for antenatal care and skilled attendance at birth by ANMs/LHVs/SNs. In: Division MH, ed. New Delhi: GoI, 2010.
25. Sayed S, Cherniak W, Lawler M, et al. Improving pathology and laboratory medicine in low-income and middle-income countries: roadmap to solutions. Lancet 2018;**391**(10133):1939-52 doi: 10.1016/s0140-6736(18)30459-8[published Online First: Epub Date]|.
26. Horton S, Sullivan R, Flanigan J, et al. Delivering modern, high-quality, affordable pathology and laboratory medicine to low-income and middle-income countries: a call to action. Lancet 2018;**391**(10133):1953-64 doi: 10.1016/s0140-6736(18)30460-4[published Online First: Epub Date]|.
27. Karan A, Negandhi H, Hussain S, et al. Size, composition and distribution of health workforce in India: why, and where to invest? Hum Resour Health 2021;**19**(1):39 doi: 10.1186/s12960-021-00575-2[published Online First: Epub Date]|.
28. Indian Council of Medical Research. Information for testing laboratories. Secondary Information for testing laboratories 2021. https://www.icmr.gov.in/ctestlab.html.
29. The Federation of Obstetric and Gynaecological Societies of India. FOGSI Good Clinical Practice recommendation on pregnancy with Covid-19. In: Gandhi A GA, Tank P., ed.: FOGSI, 2020.
30. Dandona R, Kumar GA, Akbar M, et al. Deferred and referred deliveries contribute to stillbirths in the Indian state of Bihar: results from a population-based survey of all births. BMC Med 2019;**17**(1):28 doi: 10.1186/s12916-019-1265-1[published Online First: Epub Date]|.
31. Dandona R, Kumar GA, Bhattacharya D, et al. Distinct mortality patterns at 0-2 days versus the remaining neonatal period: results from population-based assessment in the Indian state of Bihar. BMC Med 2019;**17**(1):140 doi: 10.1186/s12916-019-1372-z[published Online First: Epub Date]|.
32. Scala C, Bhide A, Familiari A, et al. Number of episodes of reduced fetal movement at term: association with adverse perinatal outcome. Am J Obstet Gynecol 2015;**213**(5):678 e1-6 doi: 10.1016/j.ajog.2015.07.015[published Online First: Epub Date]|.
33. Dutton PJ, Warrander LK, Roberts SA, et al. Predictors of poor perinatal outcome following maternal perception of reduced fetal movements--a prospective cohort study. PLoS One 2012;**7**(7):e39784 doi: 10.1371/journal.pone.0039784[published Online First: Epub Date]|.

For peer review only - http://bmjopen.bmj.com/site/about/guidelines.xhtml

34. Huang C, Han W, Fan Y. Correlation study between increased fetal movement during the third trimester and neonatal outcome. BMC Pregnancy Childbirth 2019;**19**(1):467 doi: 10.1186/s12884-019-2637-4[published Online First: Epub Date]|.

35. India State-Level Disease Burden Initiative Malnutrition Collaborators. The burden of child and maternal malnutrition and trends in its indicators in the states of India: the Global Burden of Disease Study 1990-2017. Lancet Child Adolesc Health 2019;**3**(12):855-70 doi: 10.1016/s2352-4642(19)30273-1[published Online First: Epub Date]|.

36. World Health Organization. WHO recommendations on antenatal care for a positive pregnancy experience. Geneva: WHO, 2017.

37. Sharma J, Leslie HH, Kundu F, et al. Poor Quality for Poor Women? Inequities in the Quality of Antenatal and Delivery Care in Kenya. PLoS One 2017;**12**(1):e0171236 doi: 10.1371/journal.pone.0171236[published Online First: Epub Date]|.

38. Hodgins S, D'Agostino A. The quality-coverage gap in antenatal care: toward better measurement of effective coverage. Glob Health Sci Pract 2014;**2**(2):173-81 doi: 10.9745/ghsp-d-13-00176[published Online First: Epub Date]|.

39. Rios-Zertuche D, Zúñiga-Brenes P, Palmisano E, et al. Methods to measure quality of care and quality indicators through health facility surveys in low- and middle-income countries. Int J Qual Health Care 2019;**31**(3):183-90 doi: 10.1093/intqhc/mzy136[published Online First: Epub Date]|.

40. Morón-Duarte LS, Ramirez Varela A, Segura O, et al. Quality assessment indicators in antenatal care worldwide: a systematic review. Int J Qual Health Care 2019;**31**(7):497-505 doi: 10.1093/intqhc/mzy206[published Online First: Epub Date]|.

41. Ministry of Health and Family Welfare, Government of India. INAP: India Newborn Action Plan. New Delhi: Government of India, 2014.

42. World Health Organization P, and UNAIDS.,. Task shifting : rational redistribution of tasks among health workforce teams : global recommendations and guidelines. Geneva: WHO, PEPFAR, and UNAIDS, 2008.

43. Gautham M, Bruxvoort K, Iles R, et al. Investigating the nature of competition facing private healthcare facilities: the case of maternity care in Uttar Pradesh, India. Health Policy Plan 2019;**34**(6):450-60 doi: 10.1093/heapol/czz056[published Online First: Epub Date]|.

44. Karvande S, Sonawane D, Chavan S, et al. What does quality of care mean for maternal health providers from two vulnerable states of India? Case study of Bihar and Jharkhand. J Health Popul Nutr 2016;**35**:6 doi: 10.1186/s41043-016-0043-3[published Online First: Epub Date]|.

45. Tripathi S, Srivastava A, Memon P, et al. Quality of maternity care provided by private sector healthcare facilities in three states of India: a situational analysis. BMC Health Serv Res 2019;**19**(1):971 doi: 10.1186/s12913-019-4782-x[published Online First: Epub Date]|.

46. Zamboni K, Singh S, Tyagi M, et al. Effect of collaborative quality improvement on stillbirths, neonatal mortality and newborn care practices in hospitals of Telangana and Andhra Pradesh, India: evidence from a quasi-experimental mixed-methods study. Implement Sci 2021;**16**(1):4 doi: 10.1186/s13012-020-01058-z[published Online First: Epub Date]|.

For peer review only - http://bmjopen.bmj.com/site/about/guidelines.xhtml

527 **FIGURE LEGENDS**

528

529 **Figure 1.** Coverage of quality ANC services as reported by the pregnant women in exit interviews on
530 the PMSMA day. Bars denote 95% confidence interval, CHC Community Health Centre and SDH Sub-
531 district hospital.

532

For peer review only

Figure 1: Coverage of quality ANC services as reported by the pregnant women in exit interviews on the PMSMA day. Bars denote 95% confidence interval, CHC Community Health Centre and SDH Sub-district hospital.

A. Considering abdomen check-up for women in 1st trimester of pregnancy

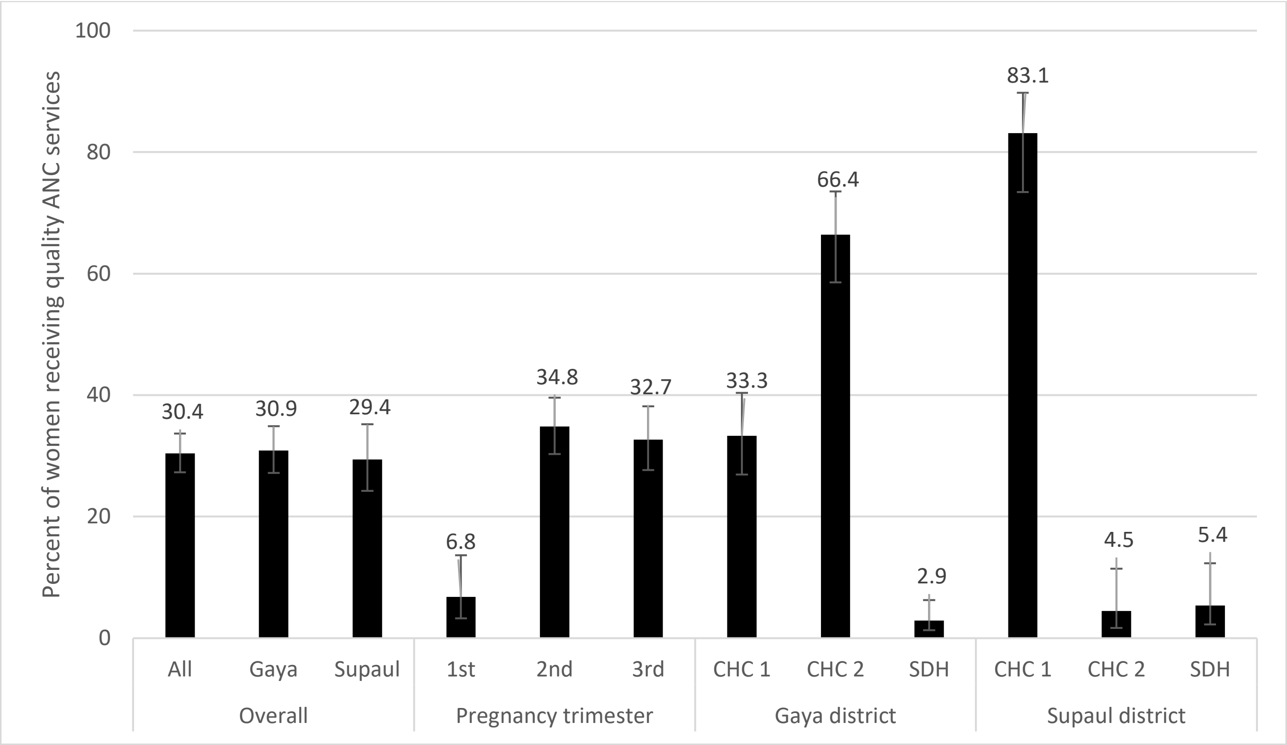

B. Not considering abdomen check-up for women in 1st trimester of pregnancy

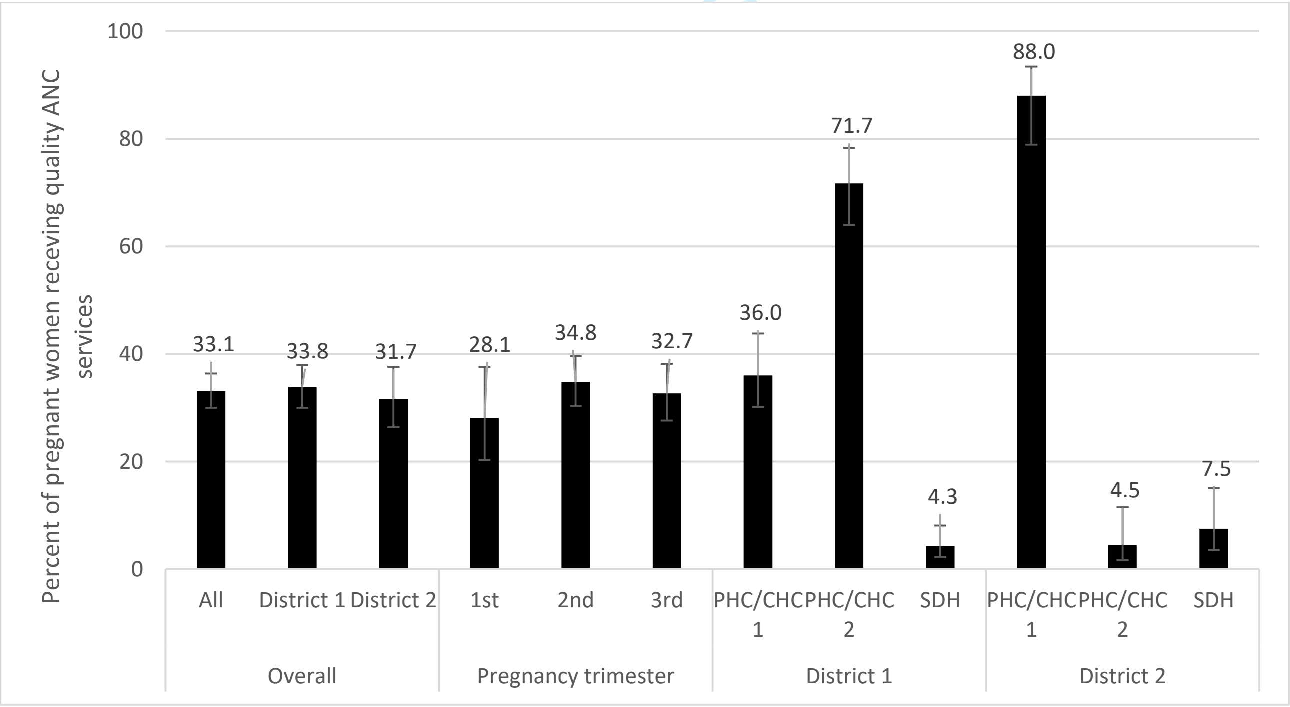

Supplementary Table 1: Select characteristics of the pregnant women who participated in the exit interviews.

Background characteristics	All women				Women in Gaya district				Women in Supaul district			
	Total N=814	In 1st trimester N =103 (%)	In 2nd trimester N=405 (%)	In 3rd trimester N=306 (%)	Total N=549	In 1st trimester of pregnancy N=69 (%)	In 2nd trimester of pregnancy N=273 (%)	In 3rd trimester of pregnancy N=207 (%)	Total N=265	In 1st trimester N=34 (%)	In 2nd trimester N=132 (%)	In 3rd trimester N=99 (%)
**Maternal age**												
15-19 years	**118**	13 (12.6)	68 (16.8)	37 (12.1)	**74**	9 (13.0)	43 (15.7)	22 (10.7)	**44**	4 (11.8)	25 (18.9)	15 (15.1)
20-24 years	**488**	69 (67.0)	242 (59.7)	176 (57.7)	**332**	46 (66.7)	159 (58.2)	126 (61.2)	**156**	23 (67.6)	83 (62.9)	50 (50.5)
25-29 years	**174**	18 (17.5)	83 (20.5)	73 (23.9)	**119**	13 (18.8)	62 (22.7)	44 (21.4)	**55**	5 (14.7)	21 (15.9)	29 (29.3)
30-40 years	**34**	3 (2.9)	12 (3.0)	19 (6. 2)	**24**	1 (1.4)	9 (3.3)	14 (6.8)	**10**	2 (5.9)	3 (2.3)	5 (5.1)
**Maternal caste***												
Scheduled caste/tribe	**259**	27 (26.7)	129 (32.3)	103 (34.3)	**201**	25 (36.2)	98 (35.9)	78 (37.7)	**58**	2 (6.3)	31 (24.4)	25 (26.9)
Other backward class	**457**	64 (63.4)	232 (58.0)	161 (53.7)	**311**	39 (56.5)	158(57.9)	114(55.1)	**146**	25 (78.1)	74 (58.3)	47 (50.5)
General	**85**	10 (9.9)	39 (9.7)	36 (12.0)	**37**	5 (7.3)	17 (6.2)	15 (7.3)	**48**	5 (15.6)	22 (17.3)	21 (22.6)
**Number of ANC visit on the day of interview**												
1st visit	**259**	61 (59.2)	134(33.1)	64 (20.9)	**189**	44 (63.7)	101 (37.0)	44 (21.3)	**70**	17 (50.0)	33 (25.0)	20 (20.2)
2nd or 3rd visit	**273**	33 (32.0)	145(35.8)	95 (31.0)	**177**	19 (27.5)	90 (32.9)	68 (32.8)	**96**	14 (41.2)	55 (41.6)	27 (27.3)
4th visit or more	**282**	9 (8.7)	126 31.1)	147 (48.0)	**183**	6 (8.7)	82 (30.0)	95 (45.9)	**99**	3 (8.8)	44 (33.3)	52 (52.5)

**Data not available for 13 pregnant women*

For peer review only

Supplementary Table 2. Coverage of quality ANC services as reported in the exit interviews on the PMSMA day with maternal age and caste of women. CI denotes confidence interval.

Background	All women					Women in Gaya district					Women in Supaul district				
	**Total N=814**	**In 1st trimester with abdomen check-up N (%; (95% CI)**	**In 1st trimester without abdomen check-up N (%; (95% CI)**	**In 2nd trimester N (%; (95% CI)**	**In 3rd trimester N (%; (95% CI)**	**Total N=549**	**In 1st trimester with abdomen check-up N (%; (95% CI)**	**In 1st trimester without abdomen check-up N (%; (95% CI))**	**In 2nd trimester N (%; (95% CI)**	**In 3rd trimester N (%; (95% CI)**	**Total N=265**	**In 1st trimester with abdomen check-up N (%; (95% CI)**	**In 1st trimester without abdomen check-up N (%; (95% CI)**	**In 2nd trimester N (%; (95% CI)**	**In 3rd trimester N (%; (95% CI)**
**Maternal age**															
15-19 years	**118**	15.4 (3.6-47.1)	38.5 (16.1-67.0)	32.3 (22.2-44.4)	40.5 (25.9-57.1)	**74**	0	33.3 (10.1-69.1)	34.9 (22.1-50.3)	50.0 (29.7-70.3)	**44**	50.0 (8.7-91.3)	50.0 (8.7-91.3)	28.0 (13.7-48.9)	26.7 (9.9-54.7)
20-24 years	**488**	4.3 (1.4-12.9)	24.6 (15.7-36.3)	33.5 (27.8-39.7)	29.5 (23.2-36.7)	**332**	2.2 (0.3-14.6)	28.3 (16.9-43.3)	38.4 (31.1-46.2)	28.6 (21.3-37.1)	**156**	8.7 (2.0-30.7)	17.4 (6.3-39.8)	24.1 (16.0-34.6)	32.0 (20.4-46.3)
25-29 years	**174**	11.1 (2.6-36.6)	27.8 (11.6-53.0)	40.9 (30.8-51.9)	31.5 (21.8-43.1)	**119**	15.4 (3.6-47.3)	23.1 (7.1-54.1)	40.3 (28.8-53.0)	25.0 (14.3-40.0)	**55**	0	40.0 (7.7-84.2)	42.8 (23.5-64.7)	41.4 (24.8-60.2)
30-40 years	**34**	0	66.7 (9.3-97.5)	33.3 (12.4-63.7)	47.4 (26.2-69.5)	**24**	0	0	33.3 (10.2-68.6)	28.6 (10.7-57.3)	**10**	0	100.0	33.3 (2.5-90.7)	100.0
**Maternal caste***															
Scheduled caste/tribe	**259**	3.7 (0.5-23.2)	22.2 (10.1-42.1)	34.1 (26.4-42.8)	32.0 (23.7-41.7)	**201**	4.0 (0.5-25.0)	24.0 (10.8-45.0)	35.7 (26.8-45.7)	34.6 (24.8-45.9)	**58**	0	0	29.0 (15.6-47.6)	24.0 (10.9-44.9)

For peer review only

Other backward class	**457**	9.4 (4.2-19.6)	31.3 (21.0-43.8)	33.6 (27.8-40.0)	31.7 (24.9-39.3)	**311**	5.1 (1.2-19.0)	33.3 (20.1-49.8)	38.6 (31.3-46.5)	30.7 (22.9-39.8)	**146**	16.0 (5.8-37.2)	28.0 (13.3-49.6)	23.0 (14.7-34.1)	34.0 (21.8-48.9)
General	**85**	0	20.0 (4.6-56.6)	38.5 (24.5-54.6)	33.3 (19.8-50.3)	**37**	0	0	47.1 (24.9-70.4)	6.7 (0.9-37.1)	**48**	0	40.0 (7.7-84.2)	31.8 (15.6-54.1)	52.4 (31.1-72.8)

**Data not available for 13 pregnant women*

For peer review only

Supplementary Table 3: Availability of the staff on the PMSMA day to provide antenatal care services. NA denotes not applicable.

District, Facility	Number of staff							Total pregnant women registered for ANC services	Average number of pregnant women				Coverage of quality ANC services as reported in the exit interviews
	Doctor	Staff nurse	Lab technician	ANM	Counsellor	Pharmacist	Total		Per staff	Per doctor	Per ANM	Per lab technician	
**Gaya, CHC 1**													
Round 1	1	0	1	3	0	1	6	70	12	70	23.3	70	74.5
Round 2	2	0	0	4	0	1	7	69	10	34.5	17.3	NA	0
Round 3	2	0	1	5	0	1	9	74	8	37	14.8	74	28.3
**Gaya, CHC 2**													
Round 1	1	0	2	4	0	1	8	65	8	65	16.3	32.5	50.0
Round 2	1	0	2	4	0	1	8	41	5	41	10.3	20.5	90.3
Round 3	1	0	2	2	0	1	6	79	13	79	39.5	39.5	71.4
**Gaya, SDH**													
Round 1	2	2	2	2	1	1	10	89	9	44.5	44.5	44.5	4.4

For peer review only

District, Facility	Number of staff							Total pregnant women registered for ANC services	Average number of pregnant women				Coverage of quality ANC services as reported in the exit interviews
	Doctor	Staff nurse	Lab technician	ANM	Counsellor	Pharmacist	Total		Per staff	Per doctor	Per ANM	Per lab technician	
Round 2	2	0	1	2	1	1	7	71	10	35.5	35.5	71	0
Round 3	4	0	1	2	0	1	8	102	12	25.5	51	102	4.3
**Supaul, CHC 1**													
Round 1	3	0	1	12	0	0	16	122	8	40.7	10.2	122	88.9
**Supaul, CHC 2**													
Round 1	1	0	1	5	0	0	7	118	16	118	23.6	118	1.5
**Supaul, SDH**													
Round 1	2	1	2	5	1	1	12	124	10	62	24.8	62	5.1

For peer review only

Supplementary Figure 1: Coverage of ANC service components as reported by the pregnant women in exit survey on the PMSMA day. Bars denote 95% confidence interval, CHC Community Health Centre and SDH Sub-district hospital.

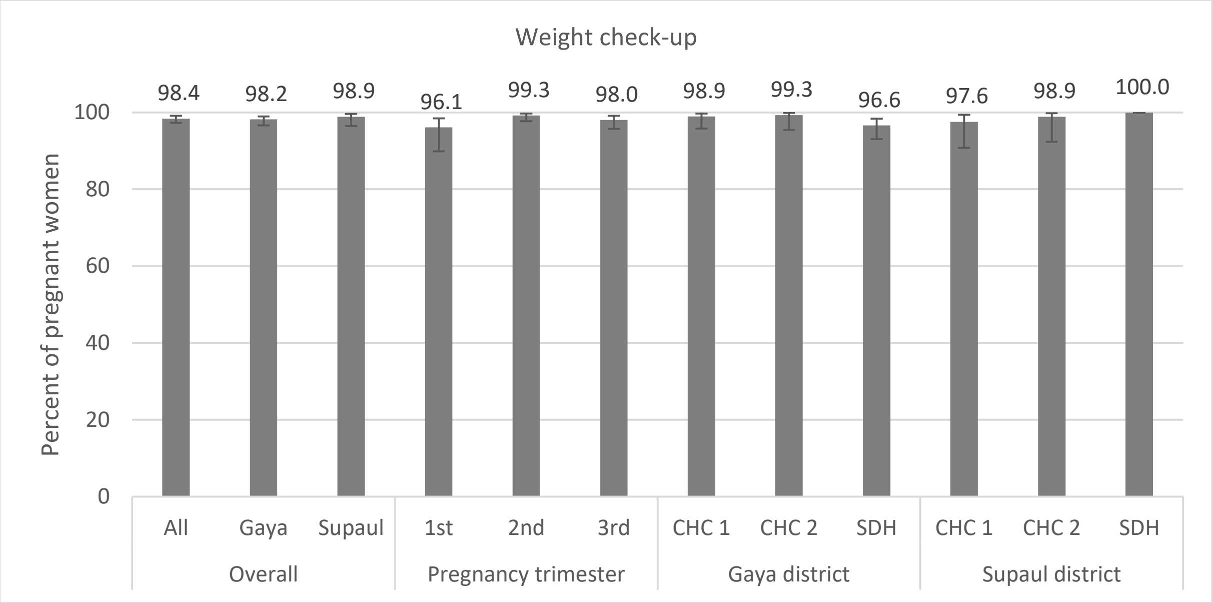

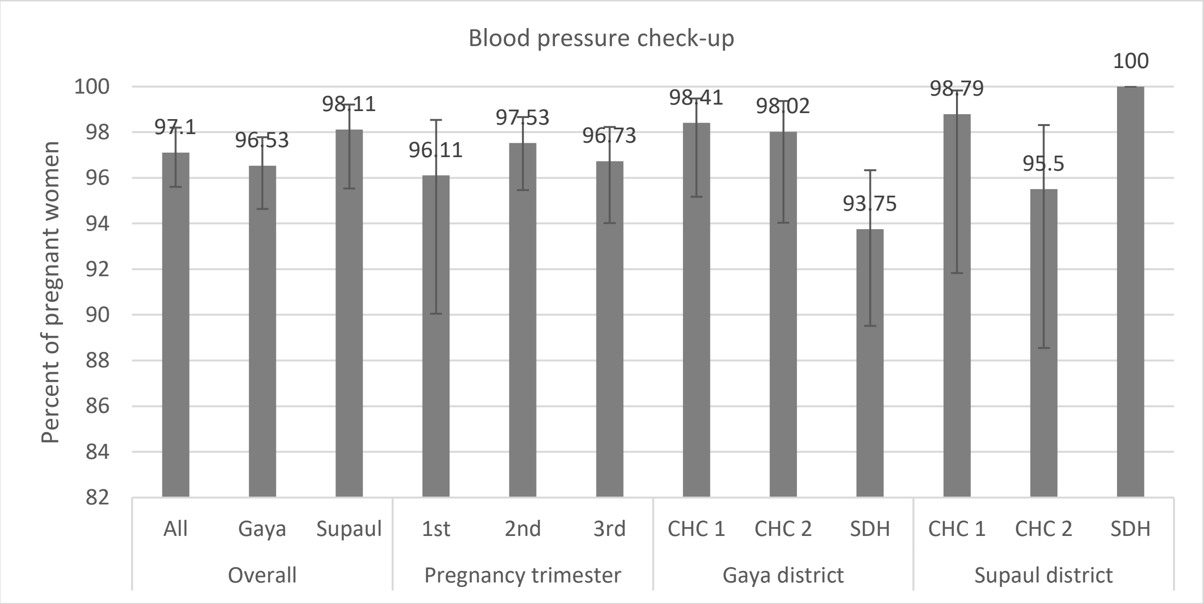

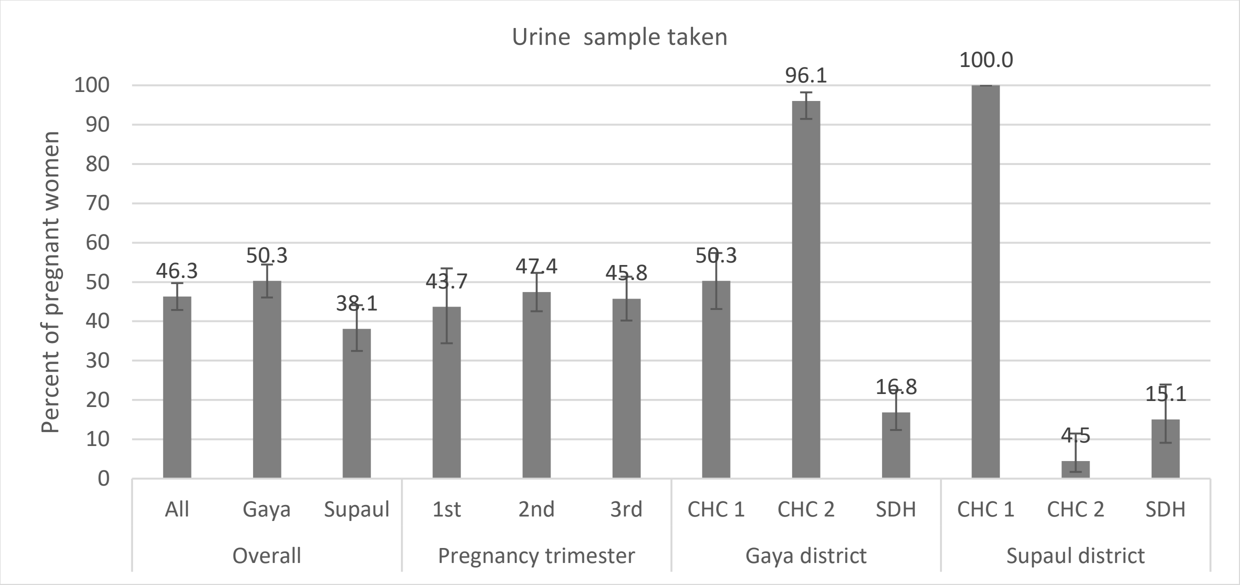
Urine sample taken
Percent of pregnant women
100
90
80
70
60
50
40
30
20
10
0
46.3
50.3
38.1
43.7
47.4
45.8
50.3
96.1
16.8
100.0
4.5
15.1
All
Gaya
Supaul
1st
2nd
3rd
CHC 1
CHC 2
SDH
CHC 1
CHC 2
SDH
Overall
Pregnancy trimester
Gaya district
Supaul district

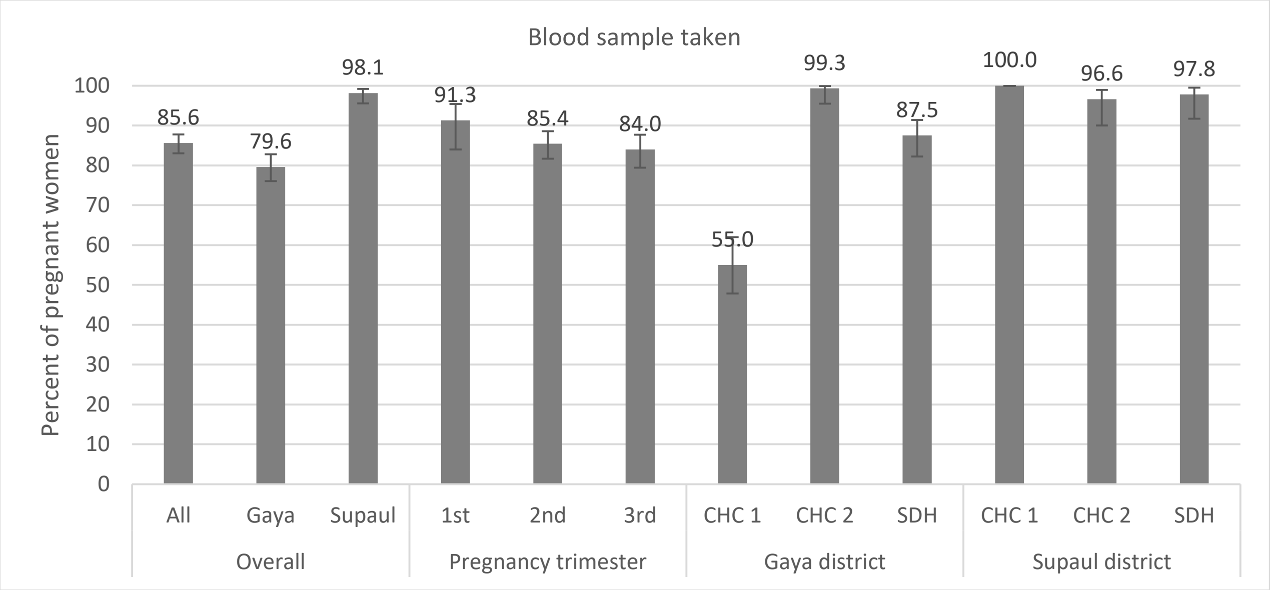
Blood sample taken
Percent of pregnant women
100
90
80
70
60
50
40
30
20
10
0
85.6
79.6
98.1
91.3
85.4
84.0
55.0
99.3
87.5
100.0
96.6
97.8
All
Gaya
Supaul
1st
2nd
3rd
CHC 1
CHC 2
SDH
CHC 1
CHC 2
SDH
Overall
Pregnancy trimester
Gaya district
Supaul district

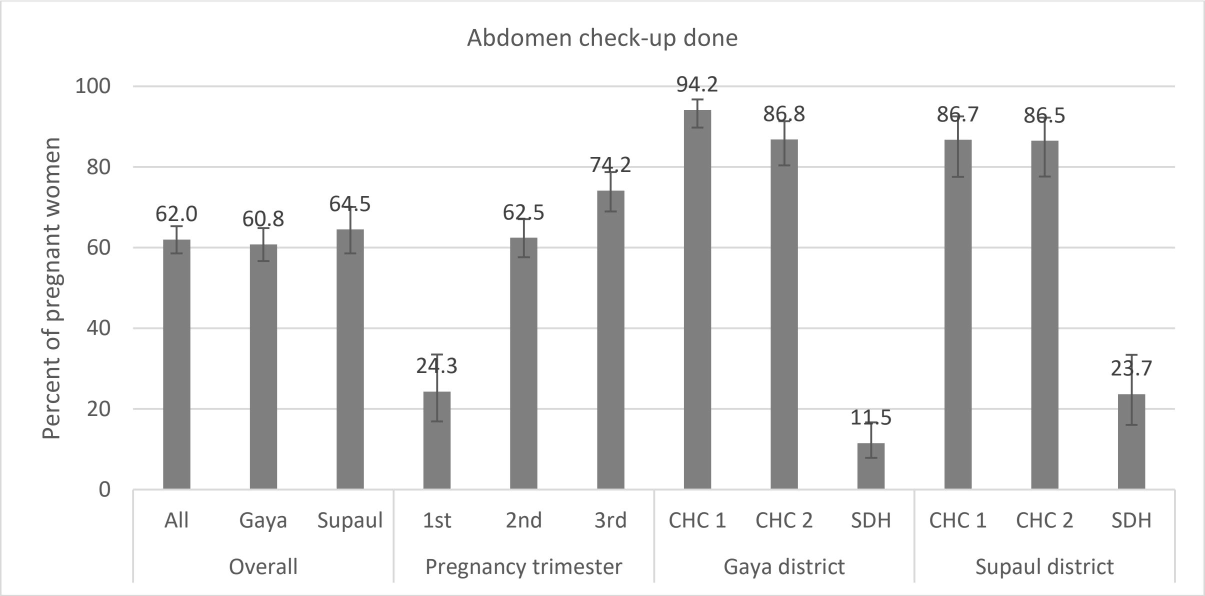

Abdomen check-up done
Percent of pregnant women
100
80
60
40
20
0
62.0
60.8
64.5
24.3
62.5
74.2
94.2
86.8
11.5
86.7
86.5
23.7
All
Gaya
Supaul
1st
2nd
3rd
CHC 1
CHC 2
SDH
CHC 1
CHC 2
SDH
Overall
Pregnancy trimester
Gaya district
Supaul district

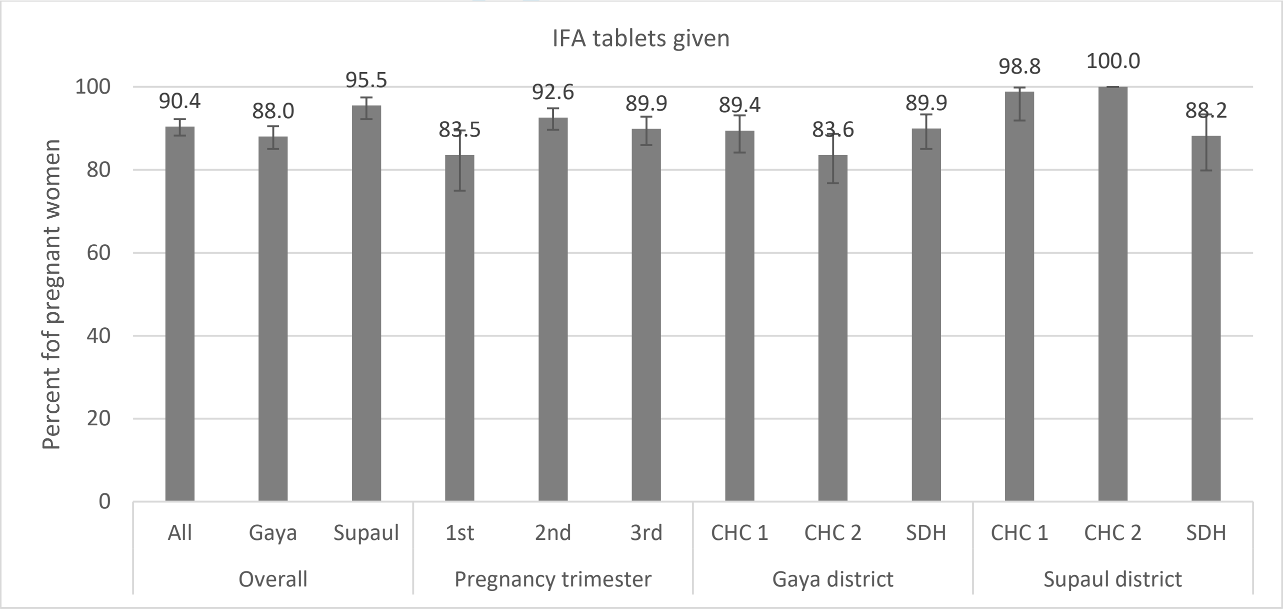

IFA tablets given
Percent fof pregnant women
100
80
60
40
20
0
90.4
88.0
95.5
83.5
92.6
89.9
89.4
83.6
89.9
98.8
100.0
88.2
All
Gaya
Supaul
1st
2nd
3rd
CHC 1
CHC 2
SDH
CHC 1
CHC 2
SDH
Overall
Pregnancy trimester
Gaya district
Supaul district

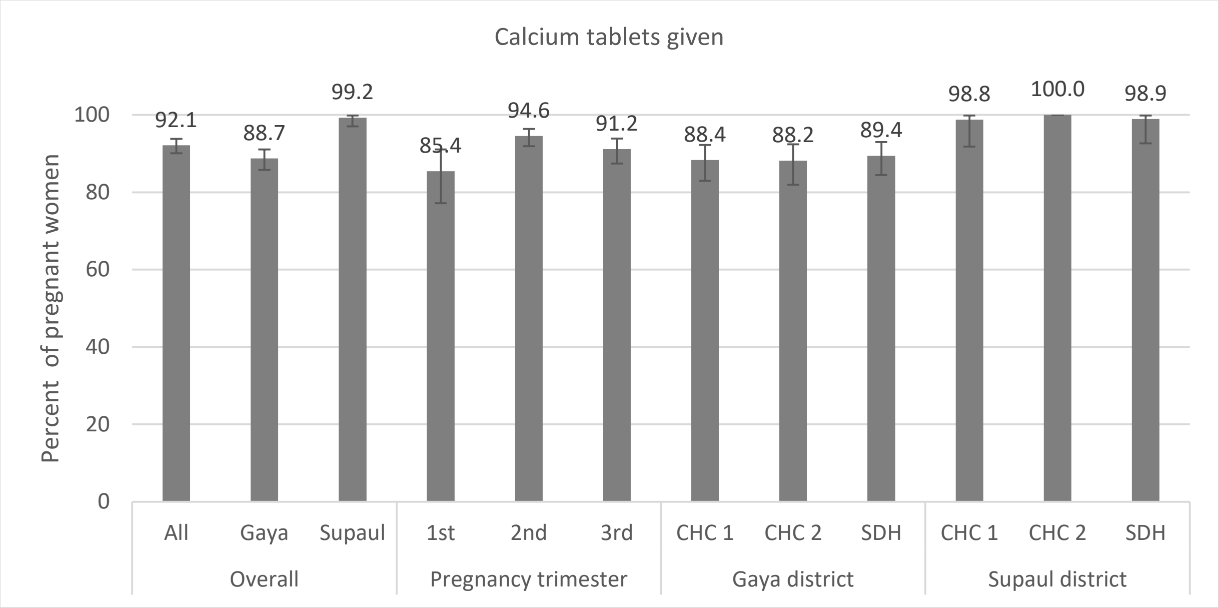

For peer review only

Supplementary Figure 2. Coverage of each component of antenatal care (ANC) and quality of ANC services among the 306 pregnant women in their 3rd trimester by the number of their ANC visit.

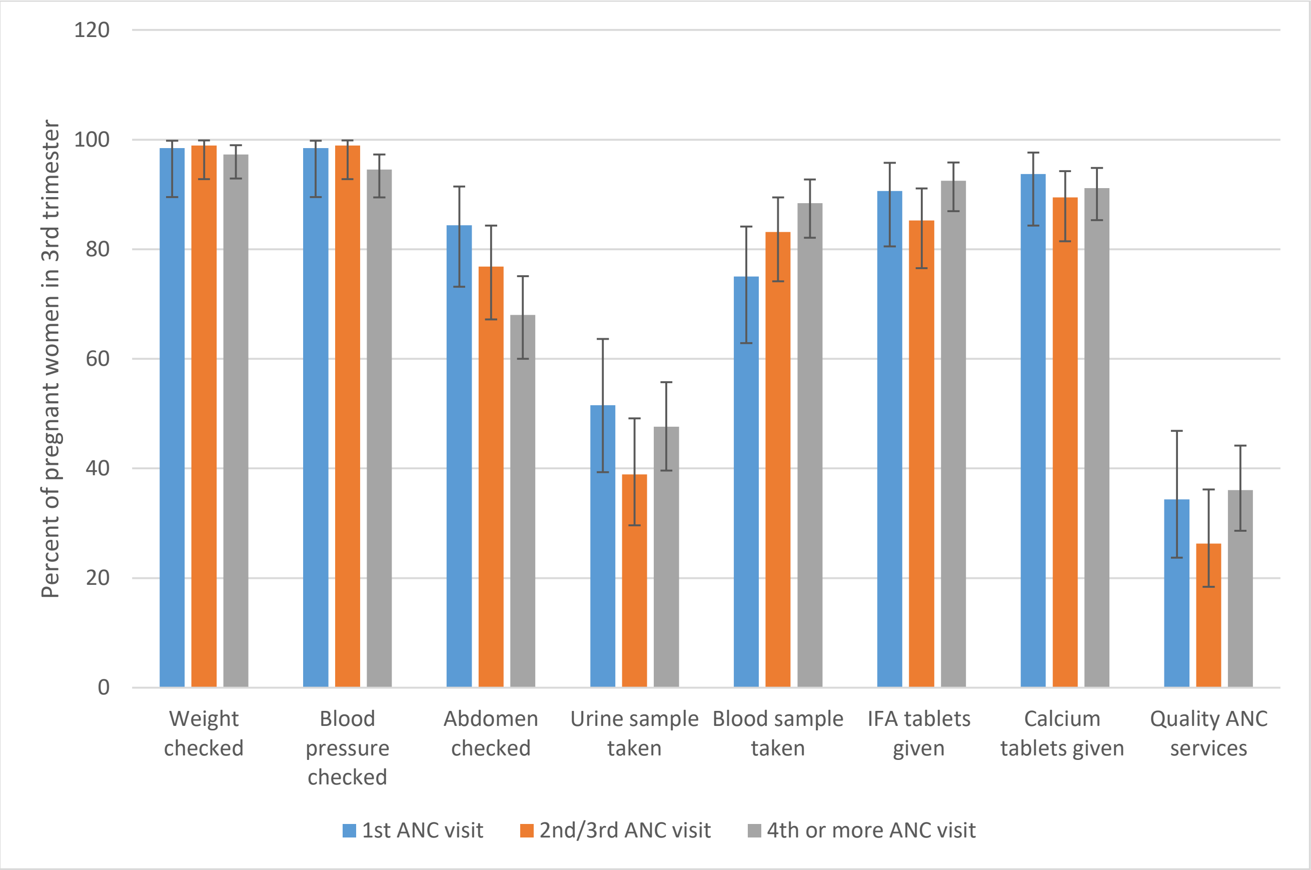

Supplmentary Figure 3: Make-shift toilet facility in one of the public sector health facilities for pregnant women to provide urine sample on PMSMA day.

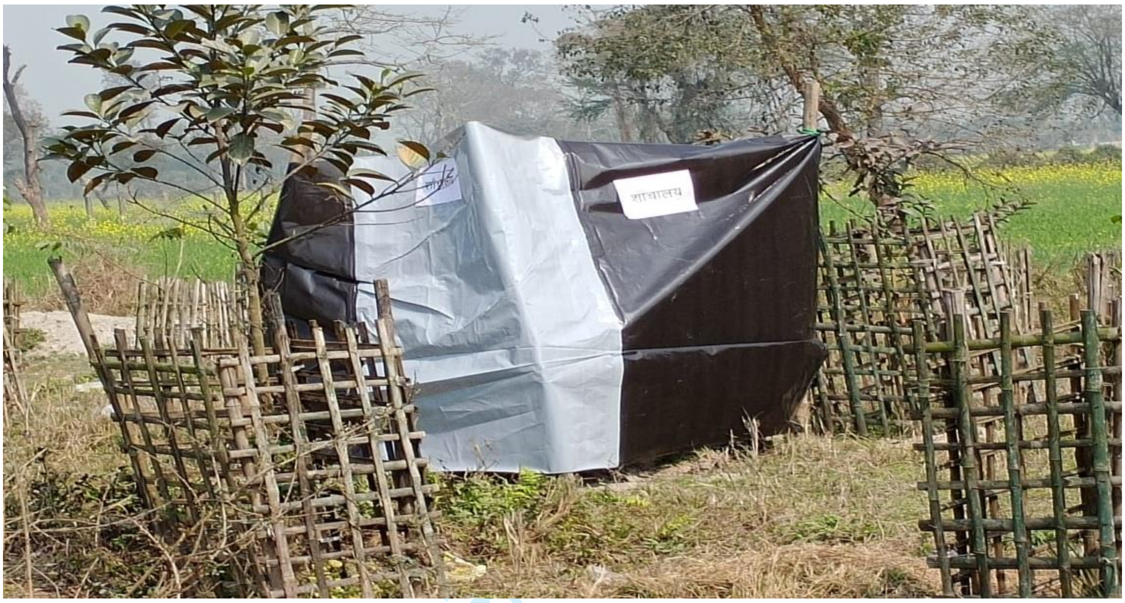

er review only

STROBE Statement—Checklist of items that should be included in reports of ***cross-sectional studies***

	Item No	Recommendation	MANUSCRIPT
**Title and abstract**	1	(*a*) Indicate the study's design with a commonly used term in the title or the abstract	Prospective term used
		(*b*) Provide in the abstract an informative and balanced summary of what was done and what was found	Provided
**Introduction**			
Background/rationale	2	Explain the scientific background and rationale for the investigation being reported	Lines 55-78
Objectives	3	State specific objectives, including any pre-specified hypotheses	Lines 75-78
**Methods**			
Study design	4	Present key elements of study design early in the paper	Done
Setting	5	Describe the setting, locations, and relevant dates, including periods of recruitment, exposure, follow-up, and data collection	Lines 80-107
Participants	6	(*a*) Give the eligibility criteria, and the sources and methods of selection of participants	Lines 86-100
Variables	7	Clearly define all outcomes, exposures, predictors, potential confounders, and effect modifiers. Give diagnostic criteria, if applicable	Lines 101-154
Data sources/ measurement	8*	For each variable of interest, give sources of data and details of methods of assessment (measurement). Describe comparability of assessment methods if there is more than one group	
Bias	9	Describe any efforts to address potential sources of bias	
Study size	10	Explain how the study size was arrived at	
Quantitative variables	11	Explain how quantitative variables were handled in the analyses. If applicable, describe which groupings were chosen and why	
Statistical methods	12	(*a*) Describe all statistical methods, including those used to control for confounding	
		(*b*) Describe any methods used to examine subgroups and interactions	
		(*c*) Explain how missing data were addressed	
		(*d*) If applicable, describe analytical methods taking account of sampling strategy	
		(*e*) Describe any sensitivity analyses	
**Results**			
Participants	13*	(a) Report numbers of individuals at each stage of study—eg numbers potentially eligible, examined for eligibility, confirmed eligible, included in the study, completing follow-up, and analysed	Lines 160-166
		(b) Give reasons for non-participation at each stage	
		(c) Consider use of a flow diagram	

For peer review only - http://bmjopen.bmj.com/site/about/guidelines.xhtml

	Item No	Recommendation	MANUSCRIPT
Descriptive data	14*	(a) Give characteristics of study participants (eg demographic, clinical, social) and information on exposures and potential confounders	
		(b) Indicate number of participants with missing data for each variable of interest	
Outcome data	15*	Report numbers of outcome events or summary measures	Table 1, Figure 1
Main results	16	(*a*) Give unadjusted estimates and, if applicable, confounder-adjusted estimates and their precision (eg, 95% confidence interval). Make clear which confounders were adjusted for and why they were included	Lines 159-275
		(*b*) Report category boundaries when continuous variables were categorized	
		(*c*) If relevant, consider translating estimates of relative risk into absolute risk for a meaningful time period	
Other analyses	17	Report other analyses done—eg analyses of subgroups and interactions, and sensitivity analyses	
**Discussion**			
Key results	18	Summarise key results with reference to study objectives	Lines 270-276
Limitations	19	Discuss limitations of the study, taking into account sources of potential bias or imprecision. Discuss both direction and magnitude of any potential bias	Lines 337-345
Interpretation	20	Give a cautious overall interpretation of results considering objectives, limitations, multiplicity of analyses, results from similar studies, and other relevant evidence	Lines 270-336
Generalisability	21	Discuss the generalisability (external validity) of the study results	
**Other information**			
Funding	22	Give the source of funding and the role of the funders for the present study and, if applicable, for the original study on which the present article is based	Line 371

*Give information separately for exposed and unexposed groups.

Note: An Explanation and Elaboration article discusses each checklist item and gives methodological background and published examples of transparent reporting. The STROBE checklist is best used in conjunction with this article (freely available on the Web sites of PLoS Medicine at http://www.plosmedicine.org/, Annals of Internal Medicine at http://www.annals.org/, and Epidemiology at http://www.epidem.com/). Information on the STROBE Initiative is available at www.strobe-statement.org.

For peer review only

BMJ Open

Assessment of quality of antenatal care services in public-sector facilities in India

Journal:	*BMJ Open*
Manuscript ID	bmjopen-2022-065200.R2
Article Type:	Original research
Date Submitted by the Author:	31-Oct-2022
Complete List of Authors:	Dandona, Rakhi; Public Health Foundation of India; University of Washington<br>Majumder, Moutushi; Public Health Foundation of India<br>Akbar, Md.; Public Health Foundation of India<br>Bhattacharya, Debarshi; Bill & Melinda Gates Foundation India<br>Nanda, Priya; Bill and Melinda Gates Foundation India<br>KUMAR, ANIL; Public Health Foundation of India<br>Dandona, Lalit; Public Health Foundation of India
**Primary Subject Heading**:	Public health
Secondary Subject Heading:	Health services research, Patient-centred medicine
Keywords:	Maternal medicine < OBSTETRICS, Community child health < PAEDIATRICS, PUBLIC HEALTH

SCHOLARONE™
Manuscripts

BMJ

I, the Submitting Author has the right to grant and does grant on behalf of all authors of the Work (as defined in the below author licence), an exclusive licence and/or a non-exclusive licence for contributions from authors who are: i) UK Crown employees; ii) where BMJ has agreed a CC-BY licence shall apply, and/or iii) in accordance with the terms applicable for US Federal Government officers or employees acting as part of their official duties; on a worldwide, perpetual, irrevocable, royalty-free basis to BMJ Publishing Group Ltd ("BMJ") its licensees and where the relevant Journal is co-owned by BMJ to the co-owners of the Journal, to publish the Work in this journal and any other BMJ products and to exploit all rights, as set out in our licence.

The Submitting Author accepts and understands that any supply made under these terms is made by BMJ to the Submitting Author unless you are acting as an employee on behalf of your employer or a postgraduate student of an affiliated institution which is paying any applicable article publishing charge ("APC") for Open Access articles. Where the Submitting Author wishes to make the Work available on an Open Access basis (and intends to pay the relevant APC), the terms of reuse of such Open Access shall be governed by a Creative Commons licence – details of these licences and which Creative Commons licence will apply to this Work are set out in our licence referred to above.

Other than as permitted in any relevant BMJ Author's Self Archiving Policies, I confirm this Work has not been accepted for publication elsewhere, is not being considered for publication elsewhere and does not duplicate material already published. I confirm all authors consent to publication of this Work and authorise the granting of this licence.

For peer review only

31 October 2022

Assessment of quality of antenatal care services in public-sector facilities in India

Rakhi Dandona,[1,2] Moutushi Majumder,[1] Md. Akbar,[1] Debarshi Bhattacharya,[3]

Priya Nanda,[3] G. Anil Kumar,[1] Lalit Dandona[1,2]

[1]Public Health Foundation of India, Gurugram, National Capital Region, India

[2]Institute for Health Metrics and Evaluation, University of Washington, Seattle, USA

[3]Bill & Melinda Gates Foundation, India Country Office, New Delhi, India

Corresponding author: Prof. Rakhi Dandona, Public Health Foundation of India, Plot 47, Sector 44, Gurugram, Haryana – 122 002, India; rakhi.dandona@phfi.org

Word count: 4,068

For peer review only

For peer review only - http://bmjopen.bmj.com/site/about/guidelines.xhtml

ABSTRACT

Objectives: We undertook assessment of quality of ANC services in public sector facilities in the Indian state of Bihar state delivered under the national ANC program (PMSMA).

Setting: Three community health centres and one sub-district hospital each in two randomly selected districts of Bihar.

Participants: Pregnant women who sought ANC services under PMSMA irrespective of the pregnancy trimester.

Primary and secondary measures: Quality ANC services was considered if a woman received all of these services in that visit - weight, blood pressure and abdomen check, urine and blood sample taken, and were given iron and folic acid and calcium tablets. The process of ANC service provision was documented.

Results: Eight hundred fourteen (94.5% participation) women participated. Coverage of quality ANC services was 30.4% (95% CI 27.3-33.7) irrespective of pregnancy trimester, and was similar in both districts and ranged 3% to 83.1% across the facilities. Quality ANC service coverage was significantly lower for women in the 1st trimester of pregnancy (6.8%, 95% CI 3.3-13.6) as compared with those in the 2nd (34.4%, 95% CI 29.9-39.1) and 3rd (32.9%, 95% CI 27.9-38.3) trimester of pregnancy. Individually, the coverage of weight and blood pressure check-up, receipt of IFA and calcium tablets, and blood sample collection was >85%. The coverage of urine sample collection was 46.3% (95% CI 42.9-49.7) and of abdomen check-up was 62% (95% CI 58.6-65.3). Poor information sharing post check-up was done with the pregnant women. Varied implementation of ANC service provision was seen in the facilities as compared with the PMSMA guidelines, in particular with laboratory diagnostics and doctor consultation. Task shifting from doctors to ANMs was observed in all facilities.

Conclusions: Grossly inadequate quality ANC services under the PMSMA needs urgent attention to improve maternal and neonatal health outcomes.

For peer review only

For peer review only - http://bmjopen.bmj.com/site/about/guidelines.xhtml

Keywords: Antenatal care, coverage, pregnancy, quality, Bihar, India

STRENGTHS AND LIMITATIONS OF THE STUDY

- Data on the quality of antenatal care services received by pregnant women documented in public sector health facilities using exit survey
- Information provided to the pregnant women after the antenatal care check-up documented in exit survey
- Direct observations made for the process of antenatal care services offered in the public sector health facilities
- Study was limited to six public sector health facilities with no private sector assessment, which could limit generalisation of the findings
- Exclusion of 10% of women due to non-availability of a phone for contact may not have any significant impact on the findings

For peer review only

For peer review only - http://bmjopen.bmj.com/site/about/guidelines.xhtml

BACKGOUND

The inadequacy of and inequity in quality of antenatal care (ANC) services is increasingly receiving attention.[1-7] The key elements of ANC services captured under the facility surveys also indicate inadequate quality of care provision.[8] Concerns about the quality of ANC services have also been reported from India, primarily based on the retrospective data collected under the National Family Health Survey.[1 9-11] The ANC quality in these reports is captured retrospectively and does not offer nuanced understanding of what happens in a particular ANC visit with a health provider , thereby, limiting the actions needed to address the coverage-quality gap in ANC services.

To increase the coverage and quality of ANC services, the Government of India started the *Pradhan Mantri Surakshit Matritva Abhiyan* (PMSMA; Prime Minister's Safe Motherhood Program).[12] PMSMA is based on the premise that if every pregnant woman in India is examined by a physician and appropriately investigated at least once during the PMSMA and then appropriately followed up, the process can result in reduction in the number of maternal and neonatal deaths in the country. With over 18,000 facilities providing PMSMA services, nearly 28.1 million pregnant women have been examined in India under this program since its inception in 2016.[12] However, no comprehensive assessment of ANC services under the PMSMA is available in the public domain. In this context , we undertook a health facility based assessment of ANC services offered on the PMSMA day in the Indian state of Bihar, one of the most populous Indian states.[13] The coverage of at least one ANC visit in the state is at 82% and that for 4 ANC visits at 25.2%,[14] with the neonatal mortality rate in 2017 at 23.4 per 1,000 livebirths and maternal mortality ratio of 230.[15 16] We report on the quality of ANC services, information sharing with the pregnant women based on the ANC check-up, and deviations from the prescribed process of ANC service provision under PMSMA with the aim to identify the areas that needed attention for the PMSMA to achieve its stated goal.

For peer review only - http://bmjopen.bmj.com/site/about/guidelines.xhtml

METHODS

Study design

A facility based cross-sectional study in two districts of Bihar state was undertaken which was approved by the Institutional review board of Public Health Foundation of India. All participants provided written informed consent, and for those who could not read or write, the participant information sheet and consent form were explained by the trained interviewer and a thumb impression obtained.

Selection of health facilities

A multi-stage sampling process was followed to select the public sector health facilities for this study. First, two districts from the state were sampled based on the latest available 3+ visits ANC coverage for Bihar for 2016 at the time of planning of this study.[17] All the 38 districts of the state were grouped into two strata as same/above and below the state median coverage for 3+ visits ANC visits (29.5%). We then randomly selected one district each from these two groups for this study – Gaya and Supaul. In each of the two sampled districts, three block-level community health centres (CHC) and one sub-district hospital (SDH) were purposively sampled from the public sector health facilities where detailed assessments were already being undertaken.

Selection of pregnant women

All pregnant women who reported for ANC services to the sampled health facilities, irrespective of the trimester of pregnancy, on the 9th day of the study month under the PMSMA scheme were considered. The only exclusion criterion was non-availability of a phone number for contact later for follow-up assessment. We aimed to recruit 200 pregnant women in each of the sampled facilities giving a total sample of 1,200 women across the six facilities.

Data collection

Data collection began in September 2019 in Gaya district but was then withheld in October and November 2019 due to floods and socio-political issues in the state, and was next two rounds were completed in December 2019 and January 2020. Data collection in Supaul district was started in

February 2020 and could not be continued due to the Covid-19 pandemic lockdown. Hence, the data available for analysis was for three rounds in Gaya district and for one round of data collection in Supaul district. The permission to conduct the study was sought from the health facility-in-charge.

Exit interviews

In each data collection round, exit interviews were conducted with the pregnant women. Interviewers trained in the study procedures contacted pregnant women after they registered at the out-patient department (OPD) registration for ANC services. Those who were able to provide a phone number were explained in brief about the purpose of the study and requested to wait after they had availed ANC services on that day. The team posted at the health facility kept track of these women through the various steps of ANC services, and re-contacted them after they had availed the services. They were provided refreshments and explained the purpose of the study in detail. Those who consented were interviewed in a semi-private place in the facility premises. The exit interviews documented socio-demographic information of the pregnant woman, details of the ANC services provided on the given PMSMA day, women's knowledge of the services received and what they were informed post the check-up.

The exit interview tool was developed in English and then translated into Hindi (local language), after which back translated into English to ensure accurate and relevant meaning and intent of the questions. Pilot testing of the tool was carried out and modifications made as necessary. The interviews were captured using the CAPI software in handheld tablets. Data entered was scrutinised using the internal checks, and some portion of the 30% of all interviews were also attended by a supervisor for quality control purpose with prior permission taken from the respondent to do so.

Observation of the flow of ANC services

The interviewers placed in each facility for the exit interviews also observed the step-wise flow of ANC service provision followed for the pregnant women. The entire process followed on a given day was documented in a format specifically developed to track the process from the beginning till the

For peer review only - http://bmjopen.bmj.com/site/about/guidelines.xhtml

end. The number and type of staff providing ANC services and the number of pregnant women registered for ANC services were documented.

Data analysis

We present the coverage of each ANC component by the pregnancy trimester, facility and district from the exit interviews. Whether pregnant women have received some or all components of a set of interventions as part of ANC at least once during pregnancy has been used to indicate quality of care.[18-20] For this analysis, we defined quality ANC service when a pregnant woman reported receiving all of the following ANC service components in the exit interview - weight measurement, blood pressure check-up, abdomen check-up, urine sample taken, blood sample taken, iron and folic acid and calcium tablets given - on that PMSMA day. We report coverage of quality ANC services by select socio-demographic and pregnancy trimester, district and type of health facility. We did not include tetanus toxoid in this assessment as it's administration in pregnant women is dependent on certain factors.[21] The tetanus toxoid coverage for last pregnancy in Bihar was reported at 89.5% in the most recent state-wide assessment.[14] Though the PMSMA is primarily designed for women in 2nd and 3rd trimester of pregnancy, in reality, women in the 1st trimester also avail ANC services on PMSMA day. Therefore, we include these women in our analysis and present findings separately by pregnancy trimester; and report coverage of quality ANC services for the women in 1st trimester of pregnancy with and without considering abdomen check-up. We also report on whether the pregnant women were informed about the check-up provided to them and of the clinical findings by the health providers.

Using the facility observations of the ANC services flow, we compared the steps followed in the ANC service provision in each facility against what is recommended in the PMSMA guidelines to highlight the issues in process that could have implications on the quality of service provision.[22] STATA 13.1 version was used for all analysis.

For peer review only

For peer review only - http://bmjopen.bmj.com/site/about/guidelines.xhtml

160 **Patient and Public Involvement statement:** Patients were not involved in planning of this research
161 study.

For peer review only

For peer review only - http://bmjopen.bmj.com/site/about/guidelines.xhtml

RESULTS

A total of 961 pregnant women were identified for exit interviews of whom 861 (89.6%) were eligible for the study, and of whom 814 (94.5%) participated. Supplementary Table 1 in Supplementary file documents the distribution of pregnant women based on the pregnancy trimester. Many of the pregnant women were in 20-24 years' age group (59.5%), 711 (87.3%) women were in their 2nd or 3rd trimester of pregnancy, and women belonging to other than forward caste (88%) accounted for most of the sample across the facilities (Supplementary Table 1 in Supplementary file).

Coverage of each ANC component

The component-wise coverage of ANC services as reported in the exit interviews by the pregnant women is shown in Supplementary Figure 1 and Supplementary Table 2 in Supplementary file. Overall, weight check-up was reported by 98.4% (95% CI 97.3-99.1) and blood pressure check-up by 97.1% (95% CI 95.7-98.2) of the pregnant women (Supplementary Figure 1 in Supplementary file). There was no significant difference in provision of these two components either by the pregnancy trimester or by the health facility (Supplementary Figure 1 in Supplementary file). Though 80% of the women for whom weight was checked were also informed of the weight reading, less than one-third of them were informed about appropriateness of the weight gain as per the pregnancy trimester (Table 1). Of the 790 women for whom blood pressure was checked, only 463 (58.7%) were informed the blood pressure reading and of them only 223 (28.2%) were informed of the appropriateness of the reading (Table 1). There was considerable variation in women being informed about blood pressure by the pregnancy trimester (Table 1).

Table 1: Distribution of pregnant women who participated in the exit interviews based on the component-wise antenatal care service provided on the given day and information provided to pregnant women regarding each of those components. CI denotes confidence interval and NA denotes not applicable.

Service provided and women informed	All women N =814 (%)	Women in 1st trimester of pregnancy N=103 % (95% CI)	Women in 2nd trimester of pregnancy N=405 % (95% CI)	Women in 3rd trimester of pregnancy N=306 % (95% CI)

For peer review only - http://bmjopen.bmj.com/site/about/guidelines.xhtml

Weight checked	801 (98.4)	99<br>96.1 (89.9-98.5)	402<br>99.3 (97.7-99.7)	300<br>98.0 (95.7-99.1)
Informed the weight reading for whom weight was checked	708 (88.4)	82<br>82. 8 (74.0-89.1)	363<br>90.3 (86.9-92.8)	263<br>87.7 (83.4-90.9)
Informed about appropriateness of weight gain by the stage of pregnancy for whom weight was checked	246 (30.7)	32<br>32.3 (23.8-42.2)	125<br>31.1 (26.7-35.8)	89<br>29.7 (24.7-35.1)
Blood pressure checked	790 (97.0)	99<br>96.1 (90.1-98.5)	395<br>97.5 (95.5-98.7)	296<br>96.7 (94.0-98.2)
Informed the blood pressure reading for whom blood pressure was checked	463 (58.7)	56<br>56.6 (46.6-66.0)	237<br>60.1 (55.2-64.9)	170<br>57.6 (51.9-63.2)
Informed about the appropriateness of blood pressure reading for whom blood pressure was checked	223 (28.2)	28<br>28.3 (20.2-38.0)	114<br>28.9 (24.6-33.5)	81<br>27.4 (22.6-32.7)
Blood sample taken	697 (85.6)	94<br>91.3 (83.9-95.4)	346<br>85.4 (81.6-88.5)	257<br>83.9 (79.4-87.7)
Informed that anaemia test will be done for whom blood sample was taken	87 (12.5)	13<br>13.8 (8.2-22.4)	43<br>12.4 (9.3-16.3)	31<br>12.1 (8.6-16.7)
Informed about the haemoglobin level for whom blood sample was taken	235 (33.7)	41<br>43.6 (33.9-53.8)	123<br>35.5 (30.7-40.7)	71<br>27.6 (22.5-33.4)
Informed about blood sugar level for whom blood sample was taken	19 (2.7)	1<br>1.1 (0.1-7.3)	12<br>3. 5 (2.0-6.0)	6<br>2.3 (1.0-5.1)
Abdomen checked	505 (62.0)	25<br>24.3 (16.9-33.5)	253<br>62.5 (57.6-67.1)	227<br>74.2 (69.0-78.8)
Explained the process to monitor baby movements for whom abdomen was checked	33 (7.7)	NA	19<br>9.4 (6.0-14.2)	15<br>6.6 (4.0-10.7)
IFA tablets given	736 (90.4)	86<br>83.5 (74.9-89.5)	375<br>92.6 (89.6-94.8)	275<br>89.8 (85.9-92.8)
Informed about the benefits and side-effects to whom IFA tablets were given	193 (26.2)	19<br>23.5 (15.4-34.0)	91<br>26.2 (21.8-31.1)	83<br>31.6 (26.2-37.4)
Calcium tablets given	750 (92.1)	88<br>85.4 (77.2-91.1)	383<br>94.6 (91.9-96.4)	279<br>91.2 (87.8-94.1)
Informed about benefits to whom calcium tablets were given	114 (15.6)	8<br>9.3 (4.7-17.6)	61<br>16.4 (13.0-20.6)	45<br>16.6 (12.6-21.5)

For peer review only

The urine sample collection coverage was 46.3% (95% CI 42.9-49.7) with significant variations seen by district, pregnancy trimester and facility (Supplementary Figure 1 in Supplementary file). The blood sample collection coverage was 85.6% (95% CI 83.0-87.8), with no significant variation by pregnancy trimester (Supplementary Figure 1 in Supplementary file). Of the 697 women for whom blood sample was taken, only 235 (33.7%) were informed about the haemoglobin value, and almost none were informed about the blood sugar value (Table 1).

The coverage of abdomen check-up was 62% (95% CI 58.6-65.3) and significant variations were seen by the pregnancy trimester and health facility (Supplementary Figure 1 in Supplementary file). Only 7.7% women of the women in 2nd or 3rd trimester were explained how to monitor baby's movements (Table 1). Overall 90.4% (95% CI 88.2-92.2) of the pregnant women reported receiving IFA tablets and 92.1% (95% CI 90.1-93.8) receiving calcium tablets, with no significant variations by pregnancy trimester or health facility (Supplementary Figure 1 in Supplementary file). Of the 736 women who had received IFA tablets, only 193 (26.2%) were informed about the benefits and side effects of it. Only 114 (15.6%) women of the 750 women who had received calcium tablets were informed about the benefits of it (Table 1).

Quality ANC services

The coverage of quality ANC services irrespective of the pregnancy trimester was 30.4% (95% CI 27.3-33.7), and was similar in both the districts (Figure 1A) and by maternal age and caste (Supplementary table 2 in Supplementary file). This coverage was significantly lower for women in the 1st trimester of pregnancy (6.8%, 95% CI 3.3-13.6) as compared with those in the 2nd or 3rd trimester of pregnancy (Figure 1A). This coverage varied significantly between the facilities, and ranged from 3-66% in district 1 and 4.5-83.1% in district 2 (Figure 1A).

On not considering the abdomen check-up for women in the 1st trimester of pregnancy (Figure 1B), quality ANC service coverage increased for them to 28.1% (95% CI 20.3- 37.6), and the pattern distribution by maternal age and caste was similar to that with inclusion of abdomen check-up (Supplementary table 2 in Supplementary file).

For peer review only - http://bmjopen.bmj.com/site/about/guidelines.xhtml

Among the 306 pregnant women in their 3rd trimester of pregnancy (Supplementary Table 1 in Supplementary file), 64 (20.9%) women had come for their 1st ANC visit, 95 (31.0%) for their 2nd or 3rd ANC visit, and 147 (48%) for their 4th ANC visit or more. Not much variation was seen in the coverage of individual ANC components based on the number of ANC visit that they were in for (Supplementary Figure 2 in Supplementary file). The coverage of quality ANC services was 34.4%, 26.3%, and 36.1% for women who had come for their 1st, 2nd or 3rd, 4th visit or more ANC visit (Supplementary Figure 2 in Supplementary file).

Process of ANC services

The number and cadre of staff available for ANC services on the PMSMA day ranged from 6 to 13 across the various rounds of data collection in the two districts (Supplementary Table 3 in Supplementary file). The average number of pregnant women examined per doctor (range 25.5 to 118), ANM (range 10.3 to 44.5) and lab technician (range 20.5 to 122) varied significantly between and within the facilities. The proportion of coverage of quality ANC services varied from 1.5% to 90.3%; a decrease in the proportion of quality ANC services was seen with increasing number of pregnant women seen per staff ($p=0.230$) and per ANM ($p=0.121$) but this was not statistically significant.

The process as per the PMSMA guidelines and what was observed in the facilities is shown in Table 2. In all the facilities, pregnant women registered for ANC check-up at the registration counter and were given “Out-patient department (OPD) slip” with a number. This OPD number was relevant only for that day and no facility had a system of tracking if a woman had visited for ANC services earlier. As per the PMSMA guidelines, step 2 is medical check-up done by ANMs followed by laboratory investigations (step 3), collection of laboratory reports (step 4), and with this the pregnant women are seen by the doctor (step 5). However, such a process was not observed in any facility (Table 2).

For peer review only - http://bmjopen.bmj.com/site/about/guidelines.xhtml

240 **Table 2: Process of antenatal care services as observed in the study facilities. CHC denotes Community Health Centre and SDH denotes Sub-district**
241 **Hospital.**

**Steps suggested in the PMSMA guidelines**		**Steps followed at the sampled facilities**					
**Step**	**Detail**	**Gaya district CHC 1**	**Gaya district CHC 2**	**Gaya district SDH**	**Supaul district CHC 1**	**Supaul district CHC 2**	**Supaul district SDH**
1	Registration	Registration	Registration	Registration	Registration	Registration	Registration
2	ANC OPD by ANM/Nurse	Doctor's consultation	Doctor's consultation	Doctor's consultation	Receipt of medicines	Laboratory investigations	ANC OPD by ANM/Nurse
3	Laboratory investigations	Laboratory investigations	Laboratory investigations	Laboratory investigations	Laboratory investigations	Collection of laboratory reports	Doctor's consultation
4	Collection of laboratory reports	Collection of laboratory reports	Collection of laboratory reports	Collection of laboratory reports	Collection of laboratory reports	ANC OPD by ANM/Nurse	Laboratory investigations
5	Doctor's consultation	ANC OPD by ANM/Nurse	ANC OPD by ANM/Nurse	ANC OPD by ANM/Nurse	Doctor's consultation	Doctor's consultation	Collection of laboratory reports
6	Counselling	Receipt of medicines	Receipt of medicines	Receipt of medicines	ANC OPD by ANM/Nurse	Receipt of medicines	Counselling
7	Receipt of medicines				Counselling		Receipt of medicines
8	Feedback and grievance redressal				Feedback and grievance redressal		Feedback and grievance redressal

242

For peer review only - http://bmjopen.bmj.com/site/about/guidelines.xhtml

Doctor consultation (step 5) was done before the steps 2-4 in 4 of the 6 facilities. Of the 22 doctors who provided ANC services in these facilities across all the rounds, 14 (63.6%) were males; 11 (50%) were MBBS, 2 (9.1 %) were MD, 4 (18.2 %) were dental specialist, 5 (22.7%) were alternate medicine doctors. A total of 349 (65.9%) and 151 (58.9%) women in Gaya and Supaul districts were seen by a doctor, respectively. The doctors were observed mainly handing over prescription indicating blood and urine tests to be done, and the list of medicines to be provided to the pregnant women in all facilities except one where the doctor performed medical check-up.

Urine sample was taken only for 276 (50.3%) and 101 (38.1%) women in Gaya and Supaul districts, respectively. In some facilities, women were given new containers for urine sample collection and in some old containers, and all tests were done only using dipstick. No facility had storage option available for urine samples. The laboratory technician noted name and serial number for each pregnant woman in the laboratory register, and indicated the same number on the container. Women were asked to go to the toilet with the container and dipstick, to dip the dipstick one inch in the urine, and bring it back to the technician. No laboratory technician was seen handling the dipstick; women were asked to hold it up and the technician recorded the reading from a distance. The field team noticed make-shift toilet (Supplementary Figure 3 in Supplementary file) in one facility and the toilet in one facility was on another floor than the ANC OPD. Pregnant women were seen hesitating in using both of these toilets, and some did not take the urine test even after being given the container. In the remaining facilities, pregnant women had to use a toilet elsewhere which was not close to the laboratory. Some were noticed being embarrassed at carrying the container and dipstick as the other people present in the facility could see them. Blood sample was taken only for 437 (79.6%) and 260 (98.1%) women in Gaya and Supaul districts, respectively. Venous blood was drawn for 250 (35.9%) and the rest was finger prick method. The laboratory technicians were not seen wearing gloves every time they took a blood sample. Blood samples were discarded immediately after recording of the haemoglobin value in all facilities.

For peer review only - http://bmjopen.bmj.com/site/about/guidelines.xhtml

268 Almost all the check-up that the pregnant women were offered was done by the ANMs in all
269 the facilities. None of the clinical findings by the ANMs were checked by the doctor as the women
270 were made to see the doctor before any check-up was done. In all facilities, the ANMs entered the
271 clinical data in the PMSMA register next day and not immediately after examining the women. The
272 counselling services (step 6) and feedback redressal (step 7) were seen only in 2 of the 6 facilities.
273 Only 38 (18.3%) and 11 (11.8%) of the women in SDH of Gaya and Supaul districts reported meeting
274 a counsellor, respectively.
275

For peer review only

For peer review only - http://bmjopen.bmj.com/site/about/guidelines.xhtml

DISCUSSION

In our understanding, this is one of the first studies undertaken in health facility in a developing country setting that provides a systematic documentation of quality of ANC services considering all the ANC components through exit survey, along with documentation of the process of ANC service provision. Only one-third of the pregnant women had received quality ANC services under the PMSMA, and even less were informed about the services that they had received. The wide variations in the implementation of PMSMA at the facilities, and the task shifting for service provision will have to be addressed urgently to improve the quality of ANC service provision.

Two components that resulted most in poor quality of ANC services were urine examination and abdomen check-up. This study has highlighted that good clinical laboratory practice guidelines were not followed for urine examination in any of the facilities.[23] Notably, though the coverage of blood sample examination was relatively higher than that for urine, however, only blood sugar and anaemia levels were recorded and none of the other tests recommended in pregnancy were performed. These findings suggest improving the laboratory readiness is imperative to improve quality of ANC services.[24] The laboratory infrastructure and related processes, and the laboratory technicians as human resources for health have not received the necessary attention in provision of health services until recently in India.[25-27] Laboratory services have recently received unprecedented attention in India due to Covid-19, and it would be important to sustain this and expand it to routine pregnancy laboratory tests as well.[28 29]

The Indian ANC guidelines recommend abdomen check-up from 2nd pregnancy trimester onwards to monitor pregnancy progression, foetal well-being and its position.[24] We have previously reported from this population that breech position is a significant risk factor for early neonatal deaths and stillbirths, however, breech position of the baby is not known to most women before delivery as abdomen check-up is either not done or they are not informed.[30 31] Abnormal foetal movements can be used to predict adverse neonatal outcomes,[32-34] however, only 1 in 12

For peer review only - http://bmjopen.bmj.com/site/about/guidelines.xhtml

women who had received abdomen check-up was informed about monitoring of baby movements in this study.

Weight and blood pressure measurements, and provision of IFA and calcium tablets showed consistently high coverage across all the facilities irrespective of the pregnancy trimester. However, less than one-third of the pregnant women were informed about the findings or implications of these for pregnancy. This coupled with almost non-existence of counselling is a major concern. Low birthweight is reported to be the largest contributor to child malnutrition disability-adjusted-life years in India,[35] and birthweight is an intergenerational issue dependent on an interplay of various factors, including maternal undernutrition and intrauterine growth. Also, anaemia increases the risk of adverse birth outcomes, and the high burden of anaemia in Indian women has not declined since 1990.[35] With very poor communication with the women and almost no counselling, pregnant women are not empowered to take correct steps for a positive pregnancy experience and to prevent adverse birth outcomes.[36]

Substantial inequities in coverage of quality ANC services by socio-economic status have been highlighted in some previous assessments.[1 37] As this study was carried out only in the public sector facilities, we are unable to comment on inequities in general. However, it is important to note that the coverage of quality ANC services in our study was similar irrespective of the caste of women, which is a surrogate measure of socio-economic status in rural India. The new World Health Organization ANC model recommends that women attend a minimum of eight ANC visits.[36] However, with the significantly poor levels of quality ANC services as documented in this study and other assessments, focussing only on increasing the number of visits is unlikely to produce the desired maternal and neonatal health outcomes.[38] With coverage of quality ANC services at 30% on PMSMA day, the effort to improve quality of ANC can go only thus far unless the quality of ANC is explicitly tracked and monitored through standard indicators both at the health system and community levels.[38-40] Thought the India Newborn Action Plan recommends monitoring of

For peer review only

For peer review only - http://bmjopen.bmj.com/site/about/guidelines.xhtml

percentage of pregnant women who received full ANC but the definition of full ANC is not provided.[41]

The varied availability of staff on PMSMA day between and within the facilities over the various rounds of data collection in this study, and the substantial variations found in the steps of PMSMA implementation at the facilities are of concern with regards to the provision of quality services. Despite the premise of PMSMA day being that every pregnant woman in India is examined by a physician and appropriately investigated at least once during the PMSMA,[12] almost all the check-up in our study was provided by ANMs in all the facilities. Notably, the doctor's consultation did not involve any examination of the pregnant women in all but one facility even though the availability of doctors was not an issue in any facility. These findings indicate task shifting which is neither in line with the PMSMA guidelines[12] nor with the task shifting recommendations to maintain the quality of services.[42] Importantly, the program implementation needs to address and account for such task shifting to address the poor quality of ANC services. The wide variation in coverage of quality also indicates the scope for improvement. Raising the performance of all health facilities to the level of best performance should be feasible with more in-depth understanding of implementation issues at the facility level, which would lead to significant overall gains in quality and ensure that pregnant women receive quality services irrespective of the facility they access for these services.[7]

There are some limitations of the study. It was conducted in six facilities only, which could be considered as a limitation to generalise these findings. It is important to note that concerns have also been raised with the quality of maternal services in the private sector as well, and it would be important to assess quality of ANC services for these facilities.[43-46] Furthermore, we were able to conduct data collection for only one round instead of three in one district. However, it is important to note that the study findings corroborate with the previous findings of poor coverage of quality ANC services in household surveys in the state. We excluded 10% of women because of non-

For peer review only - http://bmjopen.bmj.com/site/about/guidelines.xhtml

For peer review only

availability of a phone for contacting her later for follow-up assessment. However, this proportion is small to have any significant impact on the study findings.

There are several strengths of this study. There is almost no recall bias in the information reported by the respondents on the ANC services received as this was an exit survey. The nature of this study which includes patient exit surveys immediately after ANC check-up, documentation of ANC services components beyond what is previously reported, documentation of what women are informed, and the process of ANC services are strengths of this undertaking.

For peer review only

For peer review only - http://bmjopen.bmj.com/site/about/guidelines.xhtml

CONCLUSION

This study highlights grossly inadequate quality of ANC services in public sector health facilities. The findings suggest that in order to provide ANC services as envisaged under the PMSMA, there is an urgent need to cultivate quality in ANC service provision at the public health facilities; to train doctors, ANMs and laboratory technicians to communicate with the pregnant women for a positive pregnancy experience and to address complications; and to build clinical and technical capacity and supervision for health providers to follow the standard guidelines for provision of quality ANC services.

For peer review only

For peer review only - http://bmjopen.bmj.com/site/about/guidelines.xhtml

Ethics approval: This study was approved by the Ethics Committee of the Public Health Foundation of India (TRC-IEC 410.1/19).

Authors' contributions: RD and GAK had full access to data in the study, take full responsibility for the integrity of data and accuracy of the data analysis, and had final responsibility for the decision to submit for publication; RD, GAK and LD conceptualized the study; RD guided the data analysis and drafted the manuscript; MM and GAK performed data analysis; MA guided data collection; MA, DB, and PN contributed to data analysis and interpretation; LD contributed to drafting of the manuscript and interpretation; all authors approved the final manuscript.

Funding: Bill and Melinda Gates Foundation; grant number INV-007989.

Declaration of interests: DB and PN are employees of the Bill & Melinda Gates Foundation, India Country Office, New Delhi. The other authors have no conflict of interest.

Data availability statement sharing: All data generated or analysed during this study are included in this published article and its supplementary information files.

Acknowledgements: The authors acknowledge the contributions of Sibin George and S Siva Prasad from Public Health Foundation of India, and Asif Iqbal and Vipul Singhal from the Oxford Policy Management, India for data collection and data management.

For peer review only - http://bmjopen.bmj.com/site/about/guidelines.xhtml

REFERENCES

1. Arsenault C, Jordan K, Lee D, et al. Equity in antenatal care quality: an analysis of 91 national household surveys. Lancet Glob Health 2018;**6**(11):e1186-e95 doi: 10.1016/s2214-109x(18)30389-9[published Online First: Epub Date]|.
2. Chukwuma A, Wosu AC, Mbachu C, et al. Quality of antenatal care predicts retention in skilled birth attendance: a multilevel analysis of 28 African countries. BMC Pregnancy Childbirth 2017;**17**(1):152 doi: 10.1186/s12884-017-1337-1[published Online First: Epub Date]|.
3. Yeoh PL, Hornetz K, Shauki NIA, et al. Evaluating the quality of antenatal care and pregnancy outcomes using content and utilization assessment. Int J Qual Health Care 2018;**30**(6):466-71 doi: 10.1093/intqhc/mzy041[published Online First: Epub Date]|.
4. Sommer Albert J, Younas A, Victor G. Quality of Antenatal Care Services in a Developing Country: A Cross-Sectional Survey. Creat Nurs 2020;**26**(1):e25-e34 doi: 10.1891/1078-4535.26.1.e25[published Online First: Epub Date]|.
5. Wynne SJ, Duarte R, de Wildt G, et al. The timing and quality of antenatal care received by women attending a primary care centre in Iquitos, Peru: A facility exit survey. PLoS One 2020;**15**(3):e0229852 doi: 10.1371/journal.pone.0229852[published Online First: Epub Date]|.
6. Gwako GN, Were F, Obimbo MM, et al. Association between utilization and quality of antenatal care with stillbirths in four tertiary hospitals in a low-income urban setting. Acta Obstet Gynecol Scand 2021;**100**(4):676-83 doi: 10.1111/aogs.13956[published Online First: Epub Date]|.
7. Phommachanh S, Essink DR, Jansen M, et al. Improvement of Quality of Antenatal Care (ANC) Service Provision at the Public Health Facilities in Lao PDR: Perspective and Experiences of Supply and Demand Sides. BMC Pregnancy Childbirth 2019;**19**(1):255 doi: 10.1186/s12884-019-2345-0[published Online First: Epub Date]|.
8. Macarayan EK, Gage AD, Doubova SV, et al. Assessment of quality of primary care with facility surveys: a descriptive analysis in ten low-income and middle-income countries. Lancet Glob Health 2018;**6**(11):e1176-e85 doi: 10.1016/s2214-109x(18)30440-6[published Online First: Epub Date]|.
9. Singh L, Dubey R, Singh S, et al. Measuring quality of antenatal care: a secondary analysis of national survey data from India. BJOG 2019;**126 Suppl 4**:7-13 doi: 10.1111/1471-0528.15825[published Online First: Epub Date]|.
10. Munuswamy S, Nakamura K, Seino K, et al. Inequalities in Use of Antenatal Care and Its ServiceComponents in India. J Rural Med 2014;**9**(1):10-9 doi: 10.2185/jrm.2877[published Online First: Epub Date]|.
11. Bucher S, Marete I, Tenge C, et al. A prospective observational description of frequency and timing of antenatal care attendance and coverage of selected interventions from sites in Argentina, Guatemala, India, Kenya, Pakistan and Zambia. Reprod Health 2015;**12 Suppl 2**(Suppl 2):S12 doi: 10.1186/1742-4755-12-s2-s12[published Online First: Epub Date]|.
12. Ministry of Health and Family Welfare GoI. Pradhan Mantri Surakshit Matritva Abhiyan. Secondary Pradhan Mantri Surakshit Matritva Abhiyan 15 March 2021. https://pmsma.nhp.gov.in/about-scheme/#about.
13. Registrar General of India. C.D. Blockwise Primary Census Abstract Data (PCA) - Bihar. Secondary C.D. Blockwise Primary Census Abstract Data (PCA) - Bihar 2011. http://censusindia.gov.in/pca/cdb_pca_census/Houselisting-housing-BR.html.
14. International Institute for Population Sciences. National Family Healty Survey (NFHS-5): Bihar. Mumbai: IIPS, 2020.
15. India State-Level Disease Burden Initiative Child Mortality Collaborators. Subnational mapping of under-5 and neonatal mortality trends in India: the Global Burden of Disease Study 2000-17. Lancet 2020;**395**(10237):1640-58 doi: 10.1016/s0140-6736(20)30471-2[published Online First: Epub Date]|.

For peer review only - http://bmjopen.bmj.com/site/about/guidelines.xhtml

16. Institute for Health Metrics and Evaluation (IHME). Maternal Health Atlas. Secondary Maternal Health Atlas 2018. https://maternalhealthatlas.org/.
17. International Institute for Population Sciences. National Family Healty Survey (NFHS-4) India 2015-16: Bihar. Secondary National Family Healty Survey (NFHS-4) India 2015-16: Bihar 2017. http://rchiips.org/nfhs/NFHS-4Reports/Bihar.pdf.
18. Abou Zahr CWTMWHOU. *Antenatal care in developing countries : promises, achievements, and missed opportunities : an analysis of trends, levels, and differentials, 1990-2001*. Geneva; New York: World Health Organization ; UNICEF, 2003.
19. Carvajal-Aguirre L, Amouzou A, Mehra V, et al. Gap between contact and content in maternal and newborn care: An analysis of data from 20 countries in sub-Saharan Africa. Journal of global health 2017;**7**(2):020501 doi: 10.7189/jogh.07.020501[published Online First: Epub Date]|.
20. Kyei NNA, Chansa C, Gabrysch S. Quality of antenatal care in Zambia: a national assessment. BMC Pregnancy Childbirth 2012;**12**:151-51 doi: 10.1186/1471-2393-12-151[published Online First: Epub Date]|.
21. World Health Organization. Standards for Maternal and Neonatal Care : Maternal immunization against tetanus. In: Safe DoMP, ed. Geneva: WHO.
22. Ministry of Health and Family Welfare GoI, . Pradhan Mantri Surakshit Matritva Abhiyan New Delhi, 2016.
23. Indian Council of Medical Research. ICMR guidelines for good clinical laboratory practices (GCLP) 2021. New Delhi: ICMR, 2021.
24. Ministry of Health and Family Welfare GoI, . Guidelines for antenatal care and skilled attendance at birth by ANMs/LHVs/SNs. In: Division MH, ed. New Delhi: GoI, 2010.
25. Sayed S, Cherniak W, Lawler M, et al. Improving pathology and laboratory medicine in low-income and middle-income countries: roadmap to solutions. Lancet 2018;**391**(10133):1939-52 doi: 10.1016/s0140-6736(18)30459-8[published Online First: Epub Date]|.
26. Horton S, Sullivan R, Flanigan J, et al. Delivering modern, high-quality, affordable pathology and laboratory medicine to low-income and middle-income countries: a call to action. Lancet 2018;**391**(10133):1953-64 doi: 10.1016/s0140-6736(18)30460-4[published Online First: Epub Date]|.
27. Karan A, Negandhi H, Hussain S, et al. Size, composition and distribution of health workforce in India: why, and where to invest? Hum Resour Health 2021;**19**(1):39 doi: 10.1186/s12960-021-00575-2[published Online First: Epub Date]|.
28. Indian Council of Medical Research. Information for testing laboratories. Secondary Information for testing laboratories 2021. https://www.icmr.gov.in/ctestlab.html.
29. The Federation of Obstetric and Gynaecological Societies of India. FOGSI Good Clinical Practice recommendation on pregnancy with Covid-19. In: Gandhi A GA, Tank P., ed.: FOGSI, 2020.
30. Dandona R, Kumar GA, Akbar M, et al. Deferred and referred deliveries contribute to stillbirths in the Indian state of Bihar: results from a population-based survey of all births. BMC Med 2019;**17**(1):28 doi: 10.1186/s12916-019-1265-1[published Online First: Epub Date]|.
31. Dandona R, Kumar GA, Bhattacharya D, et al. Distinct mortality patterns at 0-2 days versus the remaining neonatal period: results from population-based assessment in the Indian state of Bihar. BMC Med 2019;**17**(1):140 doi: 10.1186/s12916-019-1372-z[published Online First: Epub Date]|.
32. Scala C, Bhide A, Familiari A, et al. Number of episodes of reduced fetal movement at term: association with adverse perinatal outcome. Am J Obstet Gynecol 2015;**213**(5):678 e1-6 doi: 10.1016/j.ajog.2015.07.015[published Online First: Epub Date]|.
33. Dutton PJ, Warrander LK, Roberts SA, et al. Predictors of poor perinatal outcome following maternal perception of reduced fetal movements--a prospective cohort study. PLoS One 2012;**7**(7):e39784 doi: 10.1371/journal.pone.0039784[published Online First: Epub Date]|.

For peer review only - http://bmjopen.bmj.com/site/about/guidelines.xhtml

34. Huang C, Han W, Fan Y. Correlation study between increased fetal movement during the third trimester and neonatal outcome. BMC Pregnancy Childbirth 2019;**19**(1):467 doi: 10.1186/s12884-019-2637-4[published Online First: Epub Date]|.

35. India State-Level Disease Burden Initiative Malnutrition Collaborators. The burden of child and maternal malnutrition and trends in its indicators in the states of India: the Global Burden of Disease Study 1990-2017. Lancet Child Adolesc Health 2019;**3**(12):855-70 doi: 10.1016/s2352-4642(19)30273-1[published Online First: Epub Date]|.

36. World Health Organization. WHO recommendations on antenatal care for a positive pregnancy experience. Geneva: WHO, 2017.

37. Sharma J, Leslie HH, Kundu F, et al. Poor Quality for Poor Women? Inequities in the Quality of Antenatal and Delivery Care in Kenya. PLoS One 2017;**12**(1):e0171236 doi: 10.1371/journal.pone.0171236[published Online First: Epub Date]|.

38. Hodgins S, D'Agostino A. The quality-coverage gap in antenatal care: toward better measurement of effective coverage. Glob Health Sci Pract 2014;**2**(2):173-81 doi: 10.9745/ghsp-d-13-00176[published Online First: Epub Date]|.

39. Rios-Zertuche D, Zúñiga-Brenes P, Palmisano E, et al. Methods to measure quality of care and quality indicators through health facility surveys in low- and middle-income countries. Int J Qual Health Care 2019;**31**(3):183-90 doi: 10.1093/intqhc/mzy136[published Online First: Epub Date]|.

40. Morón-Duarte LS, Ramirez Varela A, Segura O, et al. Quality assessment indicators in antenatal care worldwide: a systematic review. Int J Qual Health Care 2019;**31**(7):497-505 doi: 10.1093/intqhc/mzy206[published Online First: Epub Date]|.

41. Ministry of Health and Family Welfare, Government of India. INAP: India Newborn Action Plan. New Delhi: Government of India, 2014.

42. World Health Organization P, and UNAIDS.,. Task shifting : rational redistribution of tasks among health workforce teams : global recommendations and guidelines. Geneva: WHO, PEPFAR, and UNAIDS, 2008.

43. Gautham M, Bruxvoort K, Iles R, et al. Investigating the nature of competition facing private healthcare facilities: the case of maternity care in Uttar Pradesh, India. Health Policy Plan 2019;**34**(6):450-60 doi: 10.1093/heapol/czz056[published Online First: Epub Date]|.

44. Karvande S, Sonawane D, Chavan S, et al. What does quality of care mean for maternal health providers from two vulnerable states of India? Case study of Bihar and Jharkhand. J Health Popul Nutr 2016;**35**:6 doi: 10.1186/s41043-016-0043-3[published Online First: Epub Date]|.

45. Tripathi S, Srivastava A, Memon P, et al. Quality of maternity care provided by private sector healthcare facilities in three states of India: a situational analysis. BMC Health Serv Res 2019;**19**(1):971 doi: 10.1186/s12913-019-4782-x[published Online First: Epub Date]|.

46. Zamboni K, Singh S, Tyagi M, et al. Effect of collaborative quality improvement on stillbirths, neonatal mortality and newborn care practices in hospitals of Telangana and Andhra Pradesh, India: evidence from a quasi-experimental mixed-methods study. Implement Sci 2021;**16**(1):4 doi: 10.1186/s13012-020-01058-z[published Online First: Epub Date]|.

For peer review only - http://bmjopen.bmj.com/site/about/guidelines.xhtml

531 **FIGURE LEGENDS**

Figure 1. Coverage of quality ANC services as reported by the pregnant women in exit interviews on the PMSMA day. Bars denote 95% confidence interval, CHC Community Health Centre and SDH Sub-district hospital.

For peer review only

For peer review only - http://bmjopen.bmj.com/site/about/guidelines.xhtml

Figure 1: Coverage of quality ANC services as reported by the pregnant women in exit interviews on the PMSMA day. Bars denote 95% confidence interval, CHC Community Health Centre and SDH Sub-district hospital.

A. Considering abdomen check-up for women in 1st trimester of pregnancy

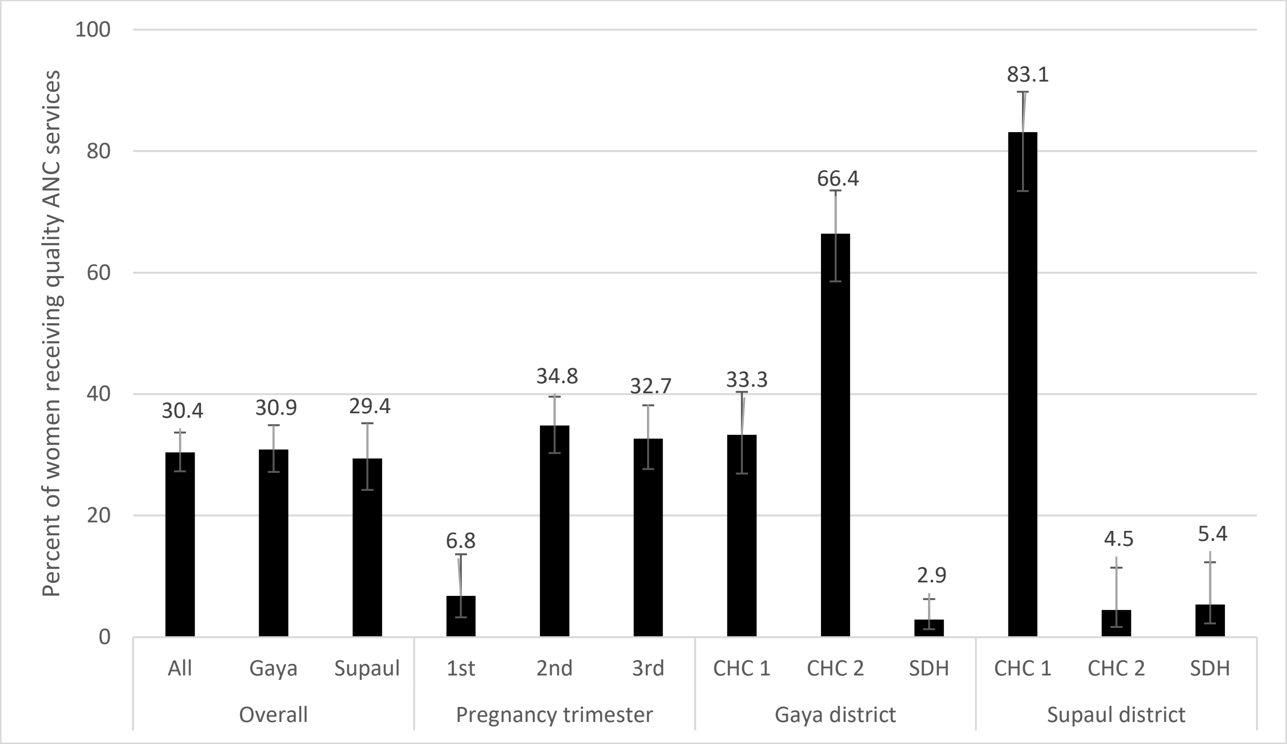

B. Not considering abdomen check-up for women in 1st trimester of pregnancy

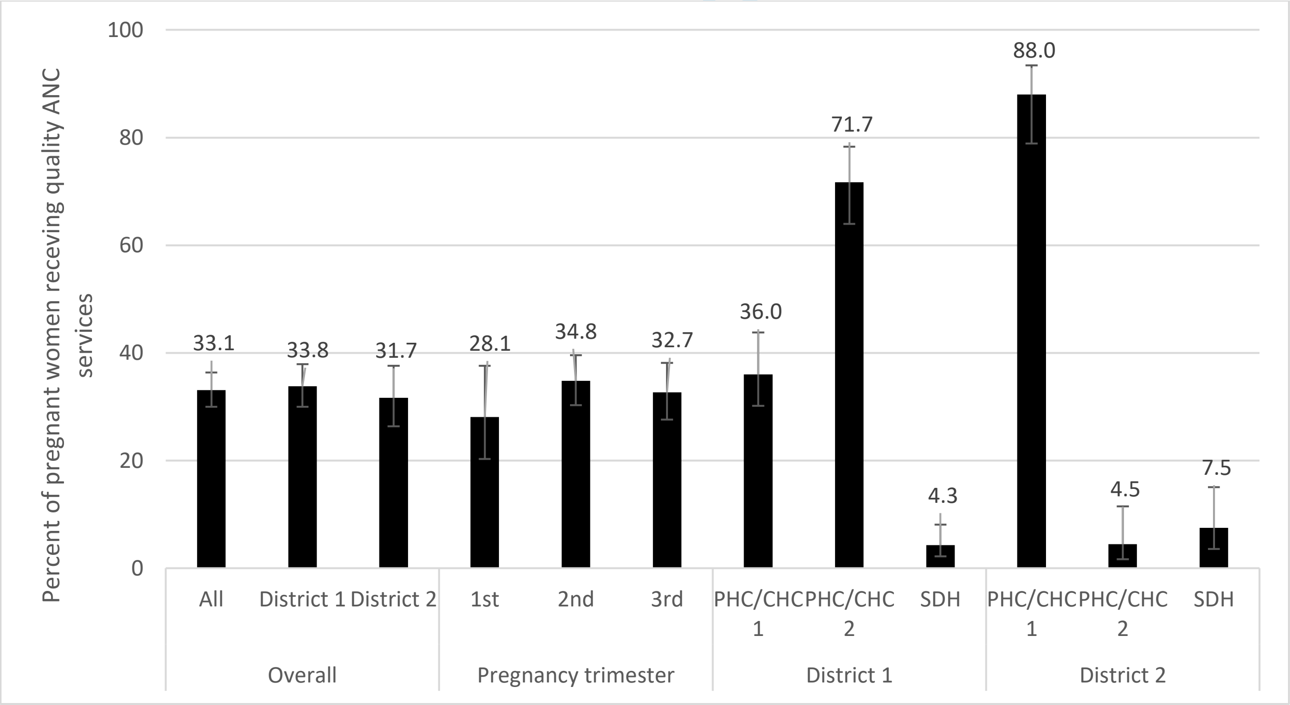

For peer review only - http://bmjopen.bmj.com/site/about/guidelines.xhtml

Supplementary Table 1: Select characteristics of the pregnant women who participated in the exit interviews.

Background characteristics	All women				Women in Gaya district				Women in Supaul district			
	Total N=814	In 1st trimester N =103 (%)	In 2nd trimester N=405 (%)	In 3rd trimester N=306 (%)	Total N=549	In 1st trimester of pregnancy N=69 (%)	In 2nd trimester of pregnancy N=273 (%)	In 3rd trimester of pregnancy N=207 (%)	Total N=265	In 1st trimester N=34 (%)	In 2nd trimester N=132 (%)	In 3rd trimester N=99 (%)
**Maternal age**												
15-19 years	**118**	13 (12.6)	68 (16.8)	37 (12.1)	**74**	9 (13.0)	43 (15.7)	22 (10.7)	**44**	4 (11.8)	25 (18.9)	15 (15.1)
20-24 years	**488**	69 (67.0)	242 (59.7)	176 (57.7)	**332**	46 (66.7)	159 (58.2)	126 (61.2)	**156**	23 (67.6)	83 (62.9)	50 (50.5)
25-29 years	**174**	18 (17.5)	83 (20.5)	73 (23.9)	**119**	13 (18.8)	62 (22.7)	44 (21.4)	**55**	5 (14.7)	21 (15.9)	29 (29.3)
30-40 years	**34**	3 (2.9)	12 (3.0)	19 (6. 2)	**24**	1 (1.4)	9 (3.3)	14 (6.8)	**10**	2 (5.9)	3 (2.3)	5 (5.1)
**Maternal caste***												
Scheduled caste/tribe	**259**	27 (26.7)	129 (32.3)	103 (34.3)	**201**	25 (36.2)	98 (35.9)	78 (37.7)	**58**	2 (6.3)	31 (24.4)	25 (26.9)
Other backward class	**457**	64 (63.4)	232 (58.0)	161 (53.7)	**311**	39 (56.5)	158(57.9)	114(55.1)	**146**	25 (78.1)	74 (58.3)	47 (50.5)
General	**85**	10 (9.9)	39 (9.7)	36 (12.0)	**37**	5 (7.3)	17 (6.2)	15 (7.3)	**48**	5 (15.6)	22 (17.3)	21 (22.6)
**Number of ANC visit on the day of interview**												
1st visit	**259**	61 (59.2)	134(33.1)	64 (20.9)	**189**	44 (63.7)	101 (37.0)	44 (21.3)	**70**	17 (50.0)	33 (25.0)	20 (20.2)
2nd or 3rd visit	**273**	33 (32.0)	145(35.8)	95 (31.0)	**177**	19 (27.5)	90 (32.9)	68 (32.8)	**96**	14 (41.2)	55 (41.6)	27 (27.3)
4th visit or more	**282**	9 (8.7)	126 31.1)	147 (48.0)	**183**	6 (8.7)	82 (30.0)	95 (45.9)	**99**	3 (8.8)	44 (33.3)	52 (52.5)

**Data not available for 13 pregnant women*

For peer review only

Supplementary Table 2. Coverage of quality ANC services as reported in the exit interviews on the PMSMA day with maternal age and caste of women. CI denotes confidence interval.

Background	All women					Women in Gaya district					Women in Supaul district				
	Total N=814	In 1st trimester with abdomen check-up N (%; (95% CI)	In 1st trimester without abdomen check-up N (%; (95% CI)	In 2nd trimester N (%; (95% CI)	In 3rd trimester N (%; (95% CI)	Total N=549	In 1st trimester with abdomen check-up N (%; (95% CI)	In 1st trimester without abdomen check-up N (%; (95% CI))	In 2nd trimester N (%; (95% CI)	In 3rd trimester N (%; (95% CI)	Total N=265	In 1st trimester with abdomen check-up N (%; (95% CI)	In 1st trimester without abdomen check-up N (%; (95% CI)	In 2nd trimester N (%; (95% CI)	In 3rd trimester N (%; (95% CI)
**Maternal age**															
15-19 years	**118**	15.4 (3.6-47.1)	38.5 (16.1-67.0)	32.3 (22.2-44.4)	40.5 (25.9-57.1)	**74**	0	33.3 (10.1-69.1)	34.9 (22.1-50.3)	50.0 (29.7-70.3)	**44**	50.0 (8.7-91.3)	50.0 (8.7-91.3)	28.0 (13.7-48.9)	26.7 (9.9-54.7)
20-24 years	**488**	4.3 (1.4-12.9)	24.6 (15.7-36.3)	33.5 (27.8-39.7)	29.5 (23.2-36.7)	**332**	2.2 (0.3-14.6)	28.3 (16.9-43.3)	38.4 (31.1-46.2)	28.6 (21.3-37.1)	**156**	8.7 (2.0-30.7)	17.4 (6.3-39.8)	24.1 (16.0-34.6)	32.0 (20.4-46.3)
25-29 years	**174**	11.1 (2.6-36.6)	27.8 (11.6-53.0)	40.9 (30.8-51.9)	31.5 (21.8-43.1)	**119**	15.4 (3.6-47.3)	23.1 (7.1-54.1)	40.3 (28.8-53.0)	25.0 (14.3-40.0)	**55**	0	40.0 (7.7-84.2)	42.8 (23.5-64.7)	41.4 (24.8-60.2)
30-40 years	**34**	0	66.7 (9.3-97.5)	33.3 (12.4-63.7)	47.4 (26.2-69.5)	**24**	0	0	33.3 (10.2-68.6)	28.6 (10.7-57.3)	**10**	0	100.0	33.3 (2.5-90.7)	100.0
**Maternal caste***															
Scheduled caste/tribe	**259**	3.7 (0.5-23.2)	22.2 (10.1-42.1)	34.1 (26.4-42.8)	32.0 (23.7-41.7)	**201**	4.0 (0.5-25.0)	24.0 (10.8-45.0)	35.7 (26.8-45.7)	34.6 (24.8-45.9)	**58**	0	0	29.0 (15.6-47.6)	24.0 (10.9-44.9)

For peer review only

Other backward class	**457**	9.4 (4.2-19.6)	31.3 (21.0-43.8)	33.6 (27.8-40.0)	31.7 (24.9-39.3)	**311**	5.1 (1.2-19.0)	33.3 (20.1-49.8)	38.6 (31.3-46.5)	30.7 (22.9-39.8)	**146**	16.0 (5.8-37.2)	28.0 (13.3-49.6)	23.0 (14.7-34.1)	34.0 (21.8-48.9)
General	**85**	0	20.0 (4.6-56.6)	38.5 (24.5-54.6)	33.3 (19.8-50.3)	**37**	0	0	47.1 (24.9-70.4)	6.7 (0.9-37.1)	**48**	0	40.0 (7.7-84.2)	31.8 (15.6-54.1)	52.4 (31.1-72.8)

**Data not available for 13 pregnant women*

For peer review only

Supplementary Table 3: Availability of the staff on the PMSMA day to provide antenatal care services. NA denotes not applicable.

District, Facility	Number of staff							Total pregnant women registered for ANC services	Average number of pregnant women				Coverage of quality ANC services as reported in the exit interviews
	Doctor	Staff nurse	Lab technician	ANM	Counsellor	Pharmacist	Total		Per staff	Per doctor	Per ANM	Per lab technician	
**Gaya, CHC 1**													
Round 1	1	0	1	3	0	1	6	70	12	70	23.3	70	74.5
Round 2	2	0	0	4	0	1	7	69	10	34.5	17.3	NA	0
Round 3	2	0	1	5	0	1	9	74	8	37	14.8	74	28.3
**Gaya, CHC 2**													
Round 1	1	0	2	4	0	1	8	65	8	65	16.3	32.5	50.0
Round 2	1	0	2	4	0	1	8	41	5	41	10.3	20.5	90.3
Round 3	1	0	2	2	0	1	6	79	13	79	39.5	39.5	71.4
**Gaya, SDH**													
Round 1	2	2	2	2	1	1	10	89	9	44.5	44.5	44.5	4.4

For peer review only

District, Facility	Number of staff							Total pregnant women registered for ANC services	Average number of pregnant women				Coverage of quality ANC services as reported in the exit interviews
	Doctor	Staff nurse	Lab technician	ANM	Counsellor	Pharmacist	Total		Per staff	Per doctor	Per ANM	Per lab technician	
Round 2	2	0	1	2	1	1	7	71	10	35.5	35.5	71	0
Round 3	4	0	1	2	0	1	8	102	12	25.5	51	102	4.3
**Supaul, CHC 1**													
Round 1	3	0	1	12	0	0	16	122	8	40.7	10.2	122	88.9
**Supaul, CHC 2**													
Round 1	1	0	1	5	0	0	7	118	16	118	23.6	118	1.5
**Supaul, SDH**													
Round 1	2	1	2	5	1	1	12	124	10	62	24.8	62	5.1

For peer review only

Supplementary Figure 1: Coverage of ANC service components as reported by the pregnant women in exit survey on the PMSMA day. Bars denote 95% confidence interval, CHC Community Health Centre and SDH Sub-district hospital.

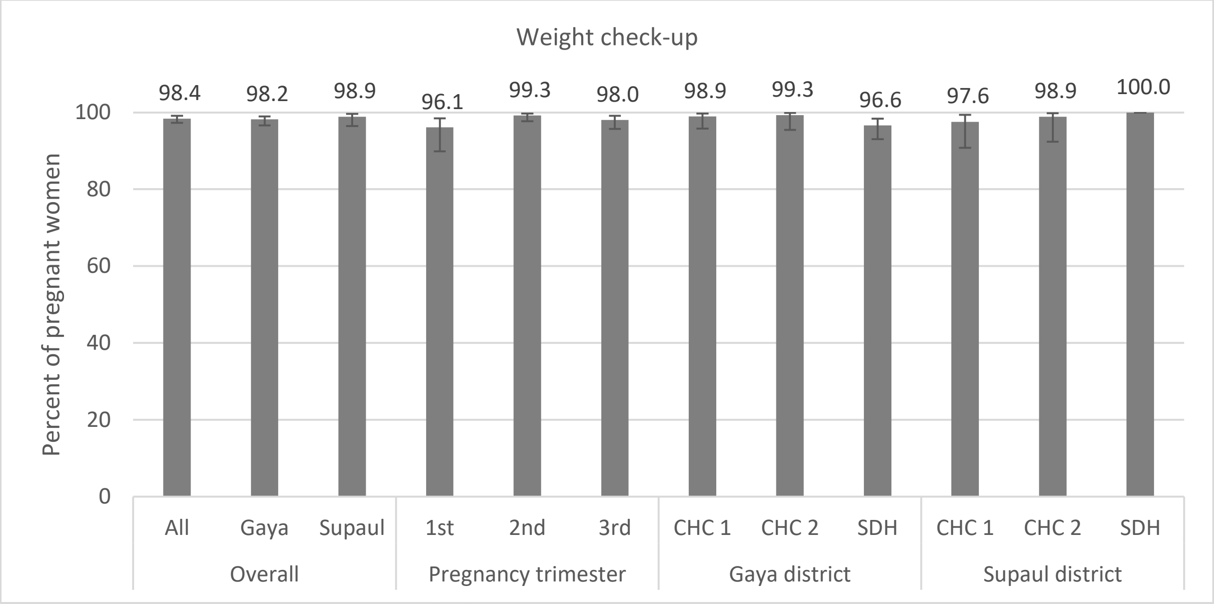

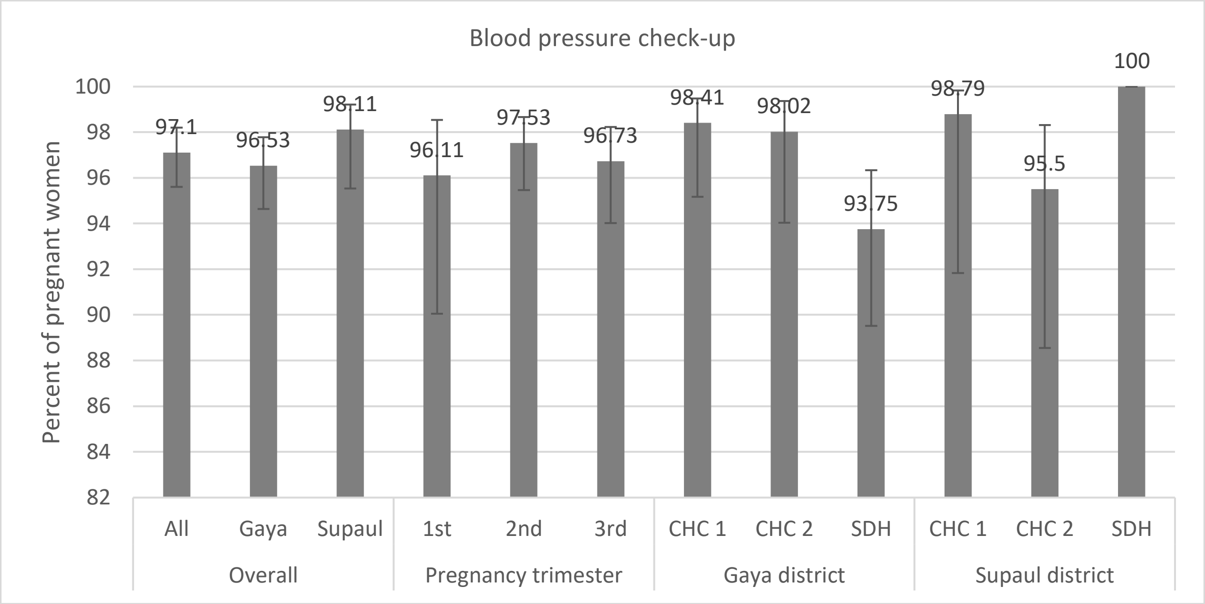

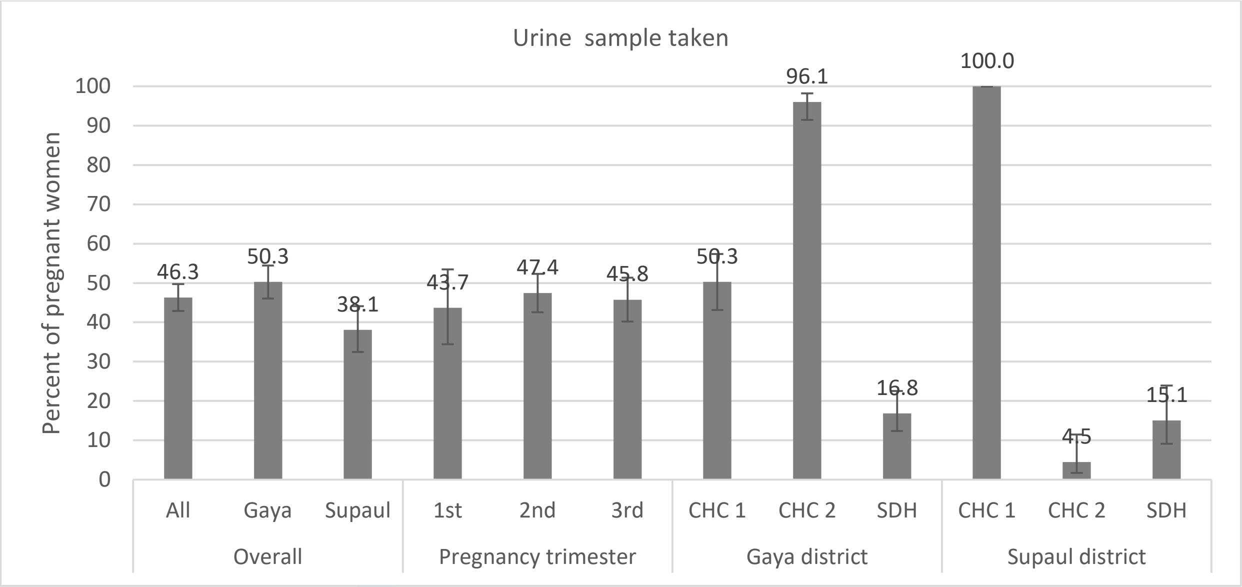
Urine sample taken
Percent of pregnant women
100
90
80
70
60
50
40
30
20
10
0
46.3
50.3
38.1
43.7
47.4
45.8
50.3
96.1
16.8
100.0
4.5
15.1
All
Gaya
Supaul
1st
2nd
3rd
CHC 1
CHC 2
SDH
CHC 1
CHC 2
SDH
Overall
Pregnancy trimester
Gaya district
Supaul district

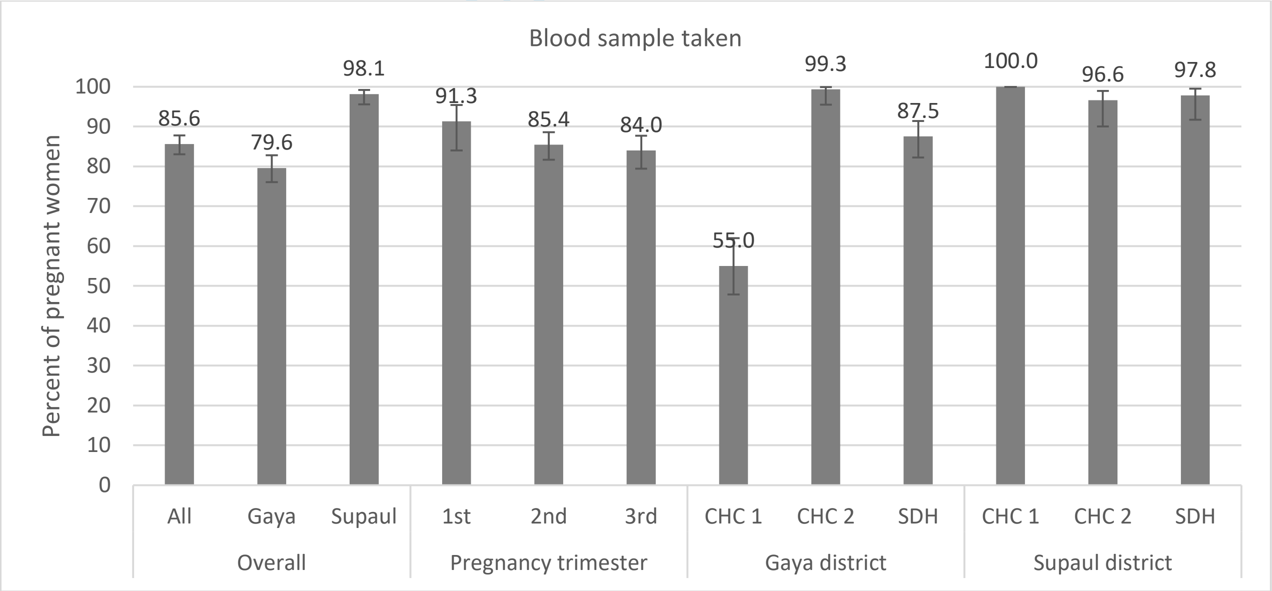
Blood sample taken
Percent of pregnant women
100
90
80
70
60
50
40
30
20
10
0
85.6
79.6
98.1
91.3
85.4
84.0
55.0
99.3
87.5
100.0
96.6
97.8
All
Gaya
Supaul
1st
2nd
3rd
CHC 1
CHC 2
SDH
CHC 1
CHC 2
SDH
Overall
Pregnancy trimester
Gaya district
Supaul district

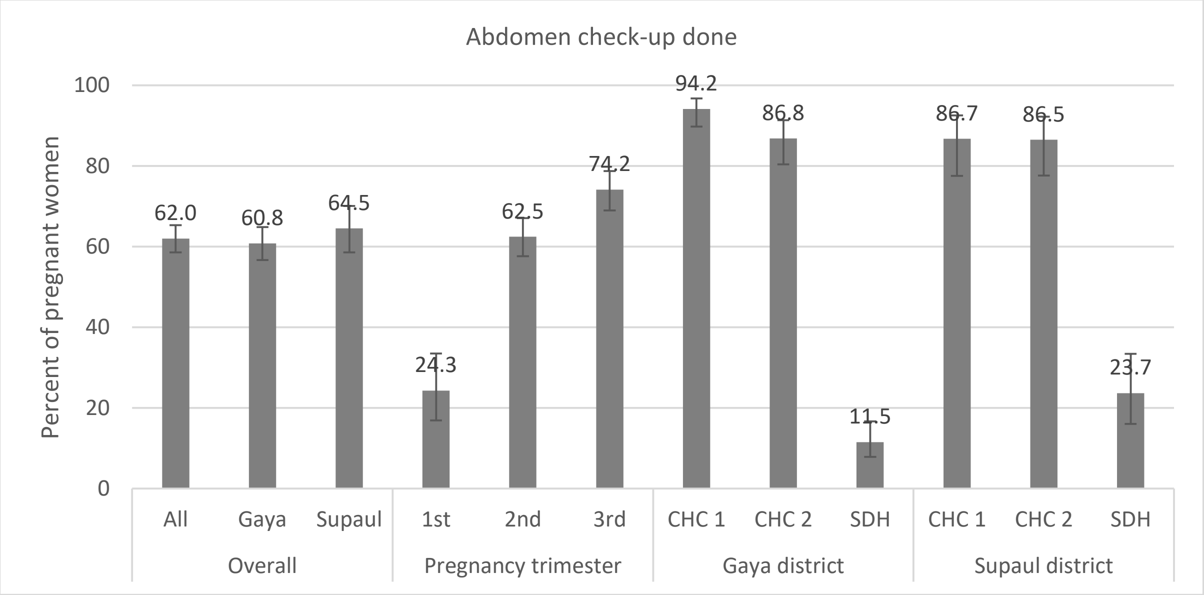
Abdomen check-up done
Percent of pregnant women
0
20
40
60
80
100
62.0
60.8
64.5
24.3
62.5
74.2
94.2
86.8
11.5
86.7
86.5
23.7
All
Gaya
Supaul
1st
2nd
3rd
CHC 1
CHC 2
SDH
CHC 1
CHC 2
SDH
Overall
Pregnancy trimester
Gaya district
Supaul district

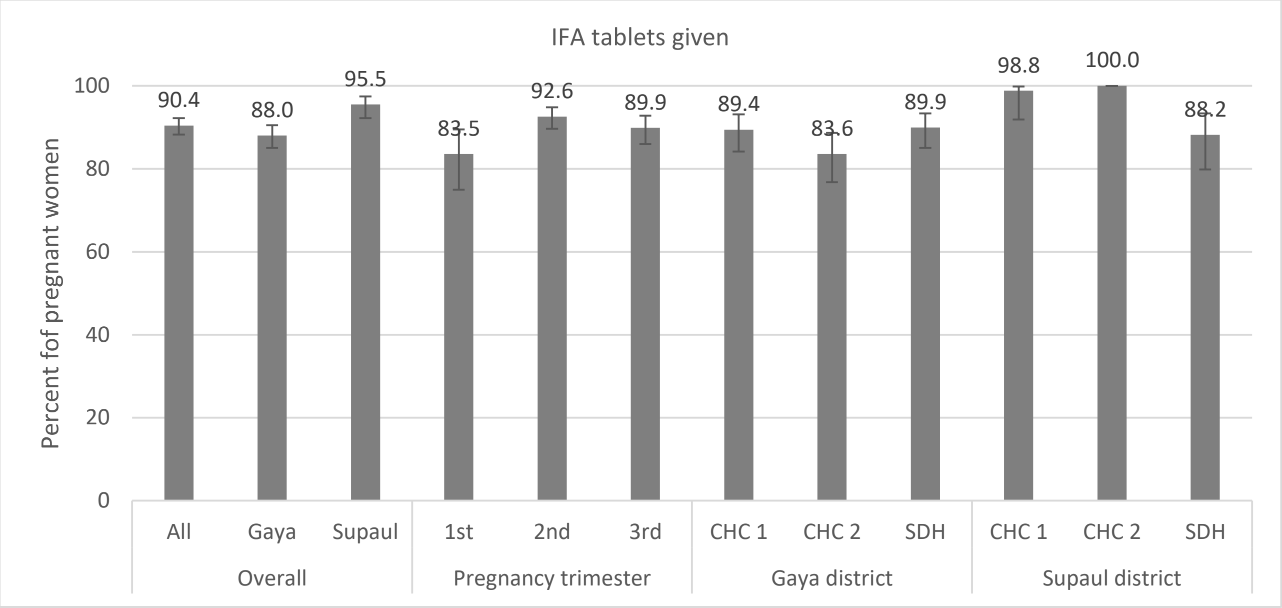
IFA tablets given
Percent fof pregnant women
0
20
40
60
80
100
90.4
88.0
95.5
83.5
92.6
89.9
89.4
83.6
89.9
98.8
100.0
88.2
All
Gaya
Supaul
1st
2nd
3rd
CHC 1
CHC 2
SDH
CHC 1
CHC 2
SDH
Overall
Pregnancy trimester
Gaya district
Supaul district

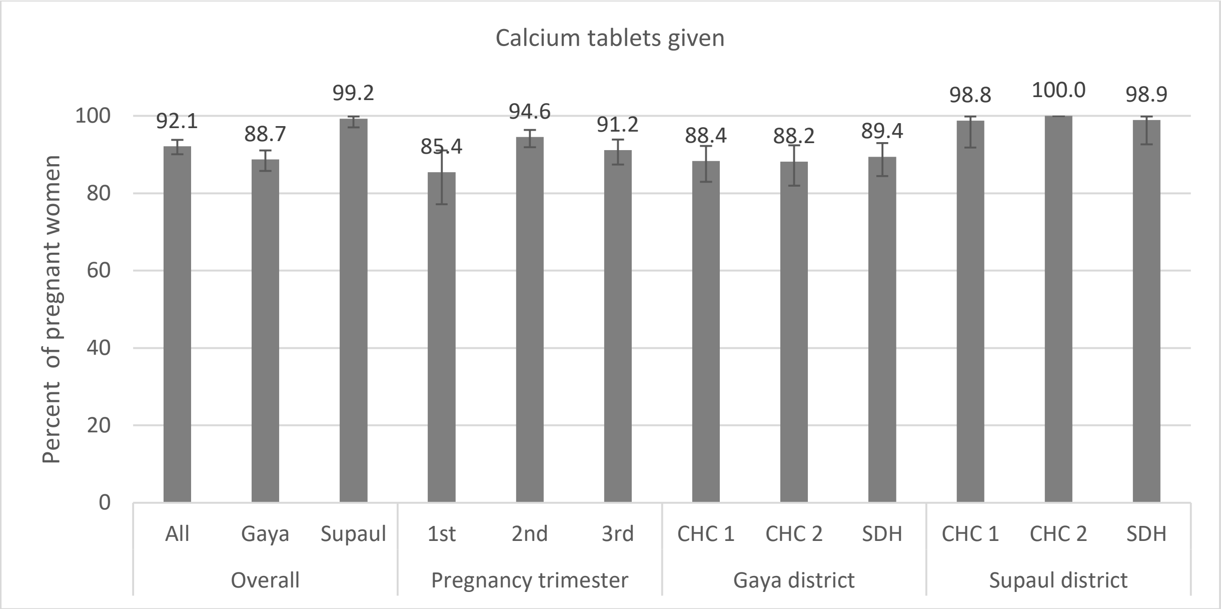

For peer review only

Supplementary Figure 2. Coverage of each component of antenatal care (ANC) and quality of ANC services among the 306 pregnant women in their 3rd trimester by the number of their ANC visit.

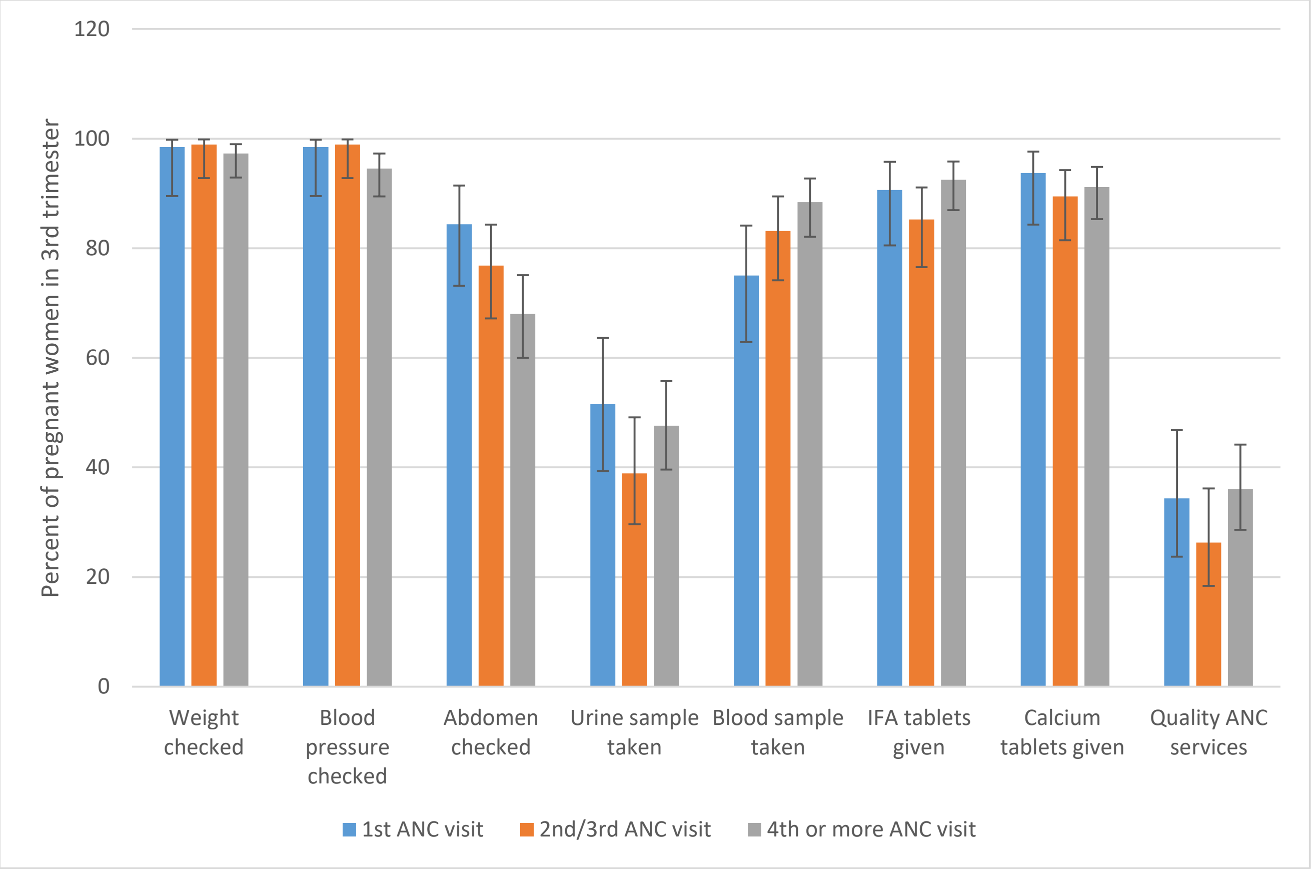

Supplmentary Figure 3: Make-shift toilet facility in one of the public sector health facilities for pregnant women to provide urine sample on PMSMA day.

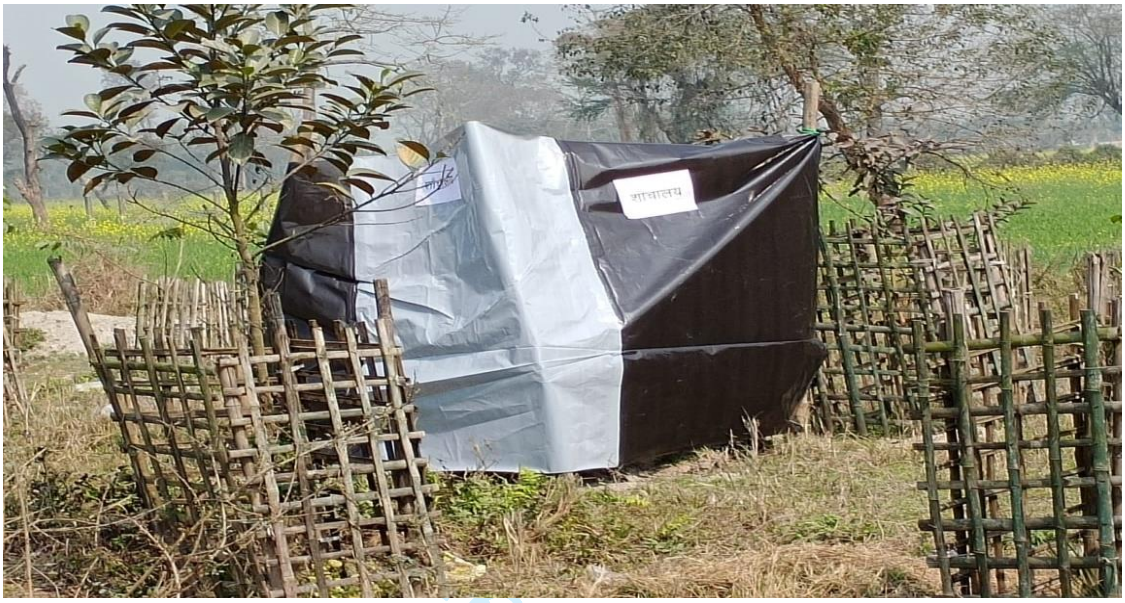

For peer review only

STROBE Statement—Checklist of items that should be included in reports of ***cross-sectional studies***

	Item No	Recommendation	MANUSCRIPT
**Title and abstract**	1	(*a*) Indicate the study's design with a commonly used term in the title or the abstract	Prospective term used
		(*b*) Provide in the abstract an informative and balanced summary of what was done and what was found	Provided
**Introduction**			
Background/rationale	2	Explain the scientific background and rationale for the investigation being reported	Lines 55-78
Objectives	3	State specific objectives, including any pre-specified hypotheses	Lines 75-78
**Methods**			
Study design	4	Present key elements of study design early in the paper	Done
Setting	5	Describe the setting, locations, and relevant dates, including periods of recruitment, exposure, follow-up, and data collection	Lines 80-107
Participants	6	(*a*) Give the eligibility criteria, and the sources and methods of selection of participants	Lines 86-100
Variables	7	Clearly define all outcomes, exposures, predictors, potential confounders, and effect modifiers. Give diagnostic criteria, if applicable	Lines 101-154
Data sources/ measurement	8*	For each variable of interest, give sources of data and details of methods of assessment (measurement). Describe comparability of assessment methods if there is more than one group	
Bias	9	Describe any efforts to address potential sources of bias	
Study size	10	Explain how the study size was arrived at	
Quantitative variables	11	Explain how quantitative variables were handled in the analyses. If applicable, describe which groupings were chosen and why	
Statistical methods	12	(*a*) Describe all statistical methods, including those used to control for confounding	
		(*b*) Describe any methods used to examine subgroups and interactions	
		(*c*) Explain how missing data were addressed	
		(*d*) If applicable, describe analytical methods taking account of sampling strategy	
		(*e*) Describe any sensitivity analyses	
**Results**			
Participants	13*	(a) Report numbers of individuals at each stage of study—eg numbers potentially eligible, examined for eligibility, confirmed eligible, included in the study, completing follow-up, and analysed	Lines 160-166
		(b) Give reasons for non-participation at each stage	
		(c) Consider use of a flow diagram	

For peer review only - http://bmjopen.bmj.com/site/about/guidelines.xhtml

	Item No	Recommendation	MANUSCRIPT
Descriptive data	14*	(a) Give characteristics of study participants (eg demographic, clinical, social) and information on exposures and potential confounders	
		(b) Indicate number of participants with missing data for each variable of interest	
Outcome data	15*	Report numbers of outcome events or summary measures	Table 1, Figure 1
Main results	16	(*a*) Give unadjusted estimates and, if applicable, confounder-adjusted estimates and their precision (eg, 95% confidence interval). Make clear which confounders were adjusted for and why they were included	Lines 159-275
		(*b*) Report category boundaries when continuous variables were categorized	
		(*c*) If relevant, consider translating estimates of relative risk into absolute risk for a meaningful time period	
Other analyses	17	Report other analyses done—eg analyses of subgroups and interactions, and sensitivity analyses	
**Discussion**			
Key results	18	Summarise key results with reference to study objectives	Lines 270-276
Limitations	19	Discuss limitations of the study, taking into account sources of potential bias or imprecision. Discuss both direction and magnitude of any potential bias	Lines 337-345
Interpretation	20	Give a cautious overall interpretation of results considering objectives, limitations, multiplicity of analyses, results from similar studies, and other relevant evidence	Lines 270-336
Generalisability	21	Discuss the generalisability (external validity) of the study results	
**Other information**			
Funding	22	Give the source of funding and the role of the funders for the present study and, if applicable, for the original study on which the present article is based	Line 371

*Give information separately for exposed and unexposed groups.

Note: An Explanation and Elaboration article discusses each checklist item and gives methodological background and published examples of transparent reporting. The STROBE checklist is best used in conjunction with this article (freely available on the Web sites of PLoS Medicine at http://www.plosmedicine.org/, Annals of Internal Medicine at http://www.annals.org/, and Epidemiology at http://www.epidem.com/). Information on the STROBE Initiative is available at www.strobe-statement.org.

For peer review only
